# In silico analyses of neuropeptide-like protein (NLP) profiles in parasitic nematodes

**DOI:** 10.1016/j.ijpara.2021.07.002

**Published:** 2022-01

**Authors:** Fiona M. McKay, Ciaran J. McCoy, Bethany Crooks, Nikki J. Marks, Aaron G. Maule, Louise E. Atkinson, Angela Mousley

**Affiliations:** Microbes & Pathogen Biology, The Institute for Global Food Security, School of Biological Sciences, Queen's University Belfast, 19 Chlorine Gardens, Belfast BT9 5DL, United Kingdom

**Keywords:** Neuropeptide, Neuropeptide-like protein, NLP, Nematode, Parasite, *Ascaris*

## Abstract

Nematode parasite infections cause disease in humans and animals and threaten global food security by reducing productivity in livestock and crop farming. The escalation of anthelmintic resistance in economically important nematode parasites underscores the need for the identification of novel drug targets in these worms. Nematode neuropeptide signalling is an attractive system for chemotherapeutic exploitation, with neuropeptide G-protein coupled receptors (NP-GPCRs) representing the lead targets. In order to successfully validate NP-GPCRs for parasite control it is necessary to characterise their function and importance to nematode biology. This can be aided through identification of receptor activating ligand(s) via deorphanisation. Such efforts require the identification of all neuropeptide ligands within parasites. Here we mined the genomes of nine therapeutically relevant pathogenic nematodes to characterise the neuropeptide-like protein complements and demonstrate that: (i) parasitic nematodes possess a reduced complement of neuropeptide-like protein-encoding genes relative to *Caenorhabditis elegans*; (ii) parasite neuropeptide-like protein profiles are broadly conserved between nematode clades; (iii) five *Ce-nlps* are completely conserved across the nematode species examined; (iv) the extent and position of neuropeptide-like protein-motif conservation is variable; (v) novel RPamide-encoding genes are present in parasitic nematodes; (vi) novel Allatostatin-C-like peptide encoding genes are present in both *C. elegans* and parasitic nematodes; (vii) novel neuropeptide-like protein families are absent in *C. elegans*; and (viii) highly conserved nematode neuropeptide-like proteins are bioactive. These data highlight the complexity of nematode neuropeptide-like proteins and reveal the need for nomenclature revision in this diverse neuropeptide family. The identification of neuropeptide-like protein ligands, and characterisation of those with functional relevance, advance our understanding of neuropeptide signalling to support exploitation of the neuropeptidergic system as an anthelmintic target.

## Highlights

• Parasites possess a reduced complement of *Caenorhabditis elegans* neuropeptide-like protein (NLP)-encoding genes.
• Parasite NLP profiles are broadly conserved between nematode clades.
• Five NLP-encoding genes are completely conserved in the nine parasitic nematodes examined.
• Fourteen novel nematode NLP encoding genes are identified.
• Several highly conserved NLPs are bioactive.

## Graphical abstract

**Figure:**
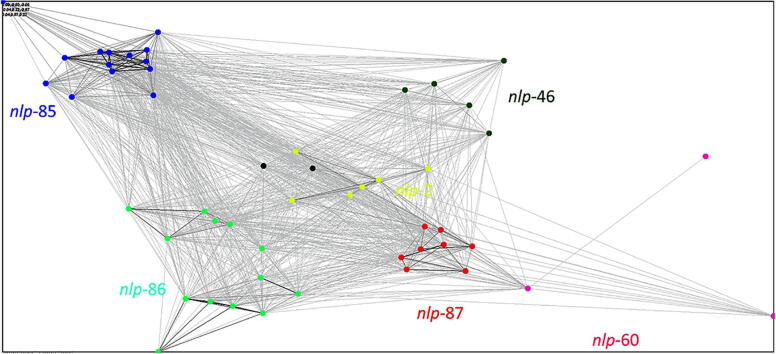

## 1 Introduction

Nematode parasites continue to threaten human health and global food security (Hotez et al., 2008, Payne and Fitchett, 2010, Elling, 2013, Roeber et al., 2013, Charlier et al., 2014, Mavrot et al., 2015, Coyne et al., 2018, Lopes-Caitar et al., 2019, Hotez and Lo, 2020). The impact of nematode parasites is exacerbated by the limited number of available anthelmintic drugs and the escalation of anthelmintic resistance in key nematode pathogens (Kaplan and Vidyashankar, 2012, Sangster et al., 2018). The disruption of nematode neuromuscular signalling is a well-established route to parasite control (McVeigh et al., 2006a, McVeigh et al., 2012, Li and Kim, 2008), where the neuropeptide signalling component represents an appealing unexploited source of novel anthelmintic targets.

Nematode neuropeptide families encompass the FMRF-amide like peptides (FLPs), neuropeptide-like proteins (NLPs) and the insulin-like peptides (INSs). Whilst INSs peptides appear to signal through receptor tyrosine kinases (Kimura et al., 1997), the majority of FLPs and NLPs are believed to act as ligands for G-protein coupled receptors (GPCRs) (Li and Kim, 2008). Within the neuropeptide system, neuropeptide GPCRs (NP-GPCRs) emerge as the most promising novel anthelmintic targets, with a proven history of ‘druggability’ (for review see McVeigh et al., 2012). Ultimately, validation of NP-GPCRs as therapeutic targets requires functional characterisation to enable the selection of the most appealing candidates.

While heterologous approaches to NP-GPCR deorphanisation in *Caenorhabditis elegans* have been successful, only a limited number of *C. elegans* NP-GPCRs have been functionally deorphanised, and only two reports of successful NP-GPCR deorphanisation have been described in parasites (Atkinson et al., 2013, Anderson et al., 2014). Whilst a major challenge of receptor deorphanisation is functional expression (McCoy et al., 2017), to aid deorphanisation efforts in therapeutically relevant nematode parasites we also require a complete profile of all potential interacting neuropeptide ligands and receptors. Recent studies have highlighted comprehensive analyses of FLP and NP-GPCR profiles in parasitic nematodes (McCoy et al., 2014), however our current understanding of NLP complements is significantly more limited (McVeigh et al., 2008, Buzy et al., 2021).

Here, we utilise an established neuropeptide Basic Local Alignment Search Tool (BLAST) approach to provide a comprehensive analyses of NLP-encoding gene profiles in nine key nematode parasites, and examine the bioactivity of the most highly conserved NLPs. These data advance understanding of nematode neuropeptide signalling and are critical to future deorphanisation efforts in parasitic nematodes that drive novel parasite control strategies.

## 2 Materials and methods

### 2.1 Species selection

*Caenorhabditis elegans nlp* gene sequelogues were identified in the genomic datasets of nine parasitic nematode species (Supplementary Table S1). The species employed in this study were selected based on a number of criteria including: (i) quality and availability of genomic and transcriptomic data, (ii) species employed in previously published neuropeptide analyses (for comparative purposes; (McCoy et al., 2014)), (iii) species appeal from a parasite control perspective (key parasites of humans, livestock and plants), (iv) representatives across clades and lifestyles, (v) species that are experimentally tractable and/or have been studied with respect to nematode neurobiology, and (iv) species for which we have amassed data on the localisation and function of neuropeptides.

### 2.2 BLAST searches

A reciprocal BLAST method was used to identify *nlp* gene sequelogues using the ‘prepropeptide search string’ approach (McCoy et al., 2014). Protein sequences for NLPs identified previously in *C. elegans* (Nathoo et al., 2001, Husson et al., 2005, Husson et al., 2007, Husson et al., 2014, McVeigh et al., 2008, Yamada et al., 2010, Howe et al., 2017, Van Bael et al., 2018; Supplementary Table S2) were obtained from WormBase (https://www.wormbase.org; WS276) and used as queries in translated nucleotide (tBLASTn) and predicted protein (BLASTp) searches of the genome datasets. tBLASTn searches were carried out on the WormBase ParaSite server (https://parasite.wormbase.org/index.html; Howe et al., 2017) ; BLASTp searches were carried out on the WormBase ParaSite Server or via a local Windows command line-based NCBI-BLAST. All BLAST searches were conducted between April 2017 and August 2018. Where there were multiple genomes available for a single species, all were mined (Supplementary Table S1). BLAST searches were also carried out in four additional *Caenorhabditis* spp. (*Caenorhabditis brenneri, Caenorhabditis briggsae, Caenorhabditis remanei* and *Caenorhabditis japonica*; as previously described (McCoy et al., 2014)). *nlp* genes (*nlp-*24–34), thought to have antimicrobial activity and distinguished by their glycine-rich sequences, were not included in this study due to their unsuitability for the BLAST search approach (McVeigh et al., 2008, Pujol et al., 2008, Dierking et al., 2016). Where *nlp* genes encoded multiple isoforms, only the longest were used as query sequences unless isoforms were significantly different. Expect values were set to ≥1000 to avoid false negative returns. All returned sequences were examined manually for NLP motifs flanked by putative mono/dibasic cleavage sites, signal peptide cleavage sites, or C-terminal signatures to eliminate false positive hits (McVeigh et al., 2008, McCoy et al., 2014) and peptides were predicted accordingly (see Supplementary Table S7) .

### 2.3 Post-BLAST analysis

Nematode parasite NLP-encoding gene sequelogues were assigned primarily based on sequence homology to the predicted peptide region of *Ce-nlps* (Li and Kim, 2008, Van Bael et al., 2018). Sequences were aligned using Vector NTI Advance 11.5 AlignX® multiple sequence alignment tool (Lu and Moriyama, 2004). Signal peptide predictions were generated using SignalP4.1 (Nielsen, 2017), however lack of a predicted signal peptide did not exclude NLP designation.

### 2.4 Clustered analysis of sequences (CLANS) analysis

The Clustered Analysis of Sequences (CLANS) algorithm was used to perform all-against-all BLASTp comparisons between the identified nematode RPamide and Allatostatin-C like prepropeptide sequences, and generate a three-dimensional (3D) similarity matrix (Frickey and Lupas, 2004). Prepropeptide sequences were analysed using the CLANS tool (https://toolkit.tuebingen.mpg.de/#/tools/clans) and the most appropriate *E*-value limit was determined to facilitate sufficient cluster separation. All other parameters were set as default. The CLANS file output was examined and coloured after 10,000 clustering rounds using the Java-based desktop software.

### 2.5 RNAseq analysis

Publicly available life-stage-specific transcriptomic data were downloaded from the WormBase ParaSite Gene Expression server (Howe et al., 2017). Raw sequence reads for *Haemonchus contortus* and *Trichuris muris* were downloaded using the NCBI SRA Toolkit (Leinonen et al., 2011). Forward and reverse fastq files were trimmed to remove adapter, leading, tailing and low quality sequences using Trimmomatic (v0.36; parameter: LEADING:5 TRAILING:5 SLIDINGWINDOW:3:15 MINLEN:34 (Bolger et al., 2014)). Appropriate genome assemblies were accessed via WormBase ParaSite FTP server for previous versions of WormBase ParaSite (Howe et al., 2017) and HISAT2 (v2.1.0 (Kim et al., 2015)) was employed to map trimmed reads to genomic data. Raw gene counts were assigned to mapped reads using SubRead v 2.0.1 featureCounts (Liao et al., 2014). Fragments per Kilobase of Transcript per Million (FPKM) data were generated via transformation of raw counts of orthologous genes using countToFPKM in R Studio and median FPKMs calculated.

### 2.6 *Ascaris suum* ovijector physiology assays

Selected *Ascaris suum* NLP peptides were tested for activity on the *A. suum* ovijector (reproductive muscle) using an established physiology assay (Fellowes et al., 1998, Fellowes et al., 2000, Moffett et al., 2003). Adult female *A. suum* (>20 cm) were collected from the intestines of pigs at a local abattoir (Karro, Northern Ireland) and transported back to the laboratory in mammalian saline (0.9% NaCl, 37 °C). Worms were maintained in *Ascaris* Ringers Solution (ARS; 13.14 mM NaCl, 9.47 mM CaCl_2_, 7.83 mM MgCl_2_, 12.09 mM C_4_H_11_NO_3_(Tris), 99.96 mM NaC_2_H_3_O_2_, 19.64 mM KCl, pH 7.8) at 37 °C and 5 % CO_2_ for up to 4 days with media changes twice daily. The ovijector was dissected from healthy, turgid worms and transferred to Hank’s balanced salt solution (HBSS; Life technologies) at 37 °C in a 4 ml recording chamber, where the tissue was attached between a flexible and an inflexible pipette. Tissue activity was amplified and recorded via a photo-optic transducer system (Fetterer et al., 1977, Marks et al., 1996). Ovijectors which displayed regular, spontaneous contractility were equilibrated for at least 5 min before peptide addition. Inactive or erratically active ovijectors were discarded. Peptides were synthesised (Genosphere Biotechnologies Inc.) and 10 mM stock solutions prepared in double distilled (dd)H_2_O, Dimethyl sulfoxide (DMSO), or Dimethyl formaldehyde (DMF) and stored in aliquots at −20 °C until use. Addition of 4 µl of ddH_2_O, DMSO or DMF had no effect on ovijector activity. Peptide was added to the 4 ml water bath such that addition of 4 µl gave a final peptide concentration of 10 µM. Peptide effects were recorded for 10 min before media were replaced with fresh HBSS. The ovijector was subsequently recorded for a further 10 min to observe recovery. Contraction amplitude and frequency were measured 2 min prior to: time 0 (peptide addition), 2, 5, 10 and 20 min post-addition. In some cases, it was necessary to analyse additional time points to capture transient effects. The change in tension was also measured at time 0, 2, 5, 10 and 20 min post-addition, where time 0 = 0 mg/mm. Note that muscle relaxation caused an increase in tension (+mg/mm; shortening of the tissue) whereas muscle contraction caused a decrease in tension (-mg/mm; lengthening of tissue). Data were statistically analysed (Graphpad Prism 8) with repeated measures ANOVA followed by Dunnett’s post-test to compare each time point with time 0.

## 3 Results and discussion

### 3.1 Parasitic nematodes possess a reduced complement of *C. elegans* NLP-encoding genes

*nlp* gene sequelogues (326) were identified in nine key nematode parasites (Table 1; Supplementary Fig. S1; Supplementary Table S3). These included 73 previously reported NLP*-*encoding genes identified through *in silico* and peptidomics studies (Nathoo et al., 2001, McVeigh et al., 2008, Jarecki et al., 2011, Koziol et al., 2016, Warnock et al., 2017, Van Bael et al., 2018, Buzy et al., 2021), and 253 novel sequences identified in this study. These data provide an insight into *nlp* conservation and diversity in nematode parasites that include multiple clades and lifestyles. As noted with parasite FLP and NP-GPCR datasets (McCoy et al., 2014) the parasitic nematode species investigated here displayed reduced complements of the *Ce*-NLP profile (Table 1). The human hookworm *Necator americanus* exhibited the largest share (67%) of *Ce-nlps*; this is unsurprising considering they are both members of clade 9 (Holterman et al., 2006). In contrast, the clade 2 species *Trichinella spiralis* and *T. muris* displayed a significantly reduced *nlp* complement of 15% and 14% of the *Ce-nlp* profile, respectively. This is likely a result of both specific gene loss in the clade 2 species, as well as *nlp* gene duplication events within the lineages that led to the extant nematodes comprising the ‘crown’ clades (Holterman et al., 2006) that exhibit broader *nlp* complements. In addition, RNASeq data show that almost all of the predicted *nlps* reported here are expressed in at least one of the parasitic nematodes examined and appear to be differentially expressed across lifecycle stages (see Supplementary Fig. S2). These data provide confidence that the *nlps* identified by our in silico approach have roles in nematode biology.

**Table 1:** *nlp*-gene sequelogues in nine nematode parasite species. A black box indicates the presence of a sequelogue identified via BLAST search.

^a^Novel gene identified in this study.

### 3.2 Parasite nlp profiles are more conserved in closely related species within the same clade

There appeared to be less intra-clade than inter-clade *nlp* profile variation, mirroring trends observed for *flps* and NP-GPCRs (Holterman et al., 2006, McCoy et al., 2014). The clade 2 nematodes (*T. spiralis, T. muris*) possess a near identical *nlp* profile, however notable differences in *nlp* profiles were observed within clade 8 species. Indeed, although the clade 8 filarids displayed near identical *nlp* profiles, they were different from that observed for *A. suum* which is also within clade 8. This is not surprising given that the filarid species are more closely related to each other than to *A. suum*, and likely reflects key differences in the lifestyles of the filarial species relative to *A. suum*.

### 3.3 Five nlps are completely conserved across key nematode species

Five *Ce-nlps* were completely conserved across all nine species interrogated in this study: *nlp*-12, -36, -47, -49 and *pdf*-1(*nlp*-74) (Table 1; Supplementary Fig. S1), highlighting their likely functional importance to nematode biology/behaviour, and supporting their presence in the last common ancestor of all nematodes. By contrast, 13 of the already annotated *Ce-nlps* were absent from all parasites examined (*nlp-*4.1, -4.2, -16, -22, -23, -39, -41, -45, -53, -65, -78, -80, and *rgba-1 (nlp*-84*)*) and so have either, been lost independently in multiple parasitic nematode lineages or, have evolved relatively recently in the specific lineage that led to *C. elegans*. Indeed, although all other *Caenorhabditis* spp. examined here possess a similar NLP profile to that of *C. elegans* (Supplementary Table S4), the other *Caenorhabditids* examined lacked *nlp-*39 and *rgba*-1/(*nlp*-84), suggesting that these genes arose following the split of the *C. elegans* lineage from *C. briggsae*, *C. brenneri* and *C. remanei* (~5–30 million years ago (MYA); Frézal and Félix, 2015). Similarly, the absence of *nlp-*4.1, -4.2, and -65 in *C. japonica* suggest that these genes likely arose following the separation of the ‘Japonica’ and *‘*Elegans’ groups (~125–190 MYA; Lemos, 2007, Frézal and Félix, 2015). Please note that we designate the *C. elegans* genes F59C6.6 and F59C6.18 as *nlp-*4.1 and *nlp*-4.2, respectively, as both genes appear to have been independently annotated as ‘*nlp-*4′ previously (see both WormBase (WS276) and Nathoo et al., 2001).

### 3.4 The extent and position of NLP motif conservation is variable

The extent of the amino acid residue conservation within the NLPs encoded on each gene varied (Supplementary Fig. S1). For example, in some instances primary sequence conservation was observed across the entire length of the encoded peptide (e.g. NLP-3, -43), however in other cases conservation was biased toward either the C- (e.g. NLP-17) or N-termini (e.g. NLP-9, -21). Some NLPs (e.g. NLP-6) displayed relatively low levels of sequence conservation across the entire length of the peptide, however this did not preclude their identification using the motif-based BLAST approach employed here. These observations suggest different levels of functional constraint between peptides, and are likely indicative of the motif that is important for receptor recognition or binding.

### 3.5 Pan-phylum in silico analyses aid NLP prediction

**Fig. 1:**
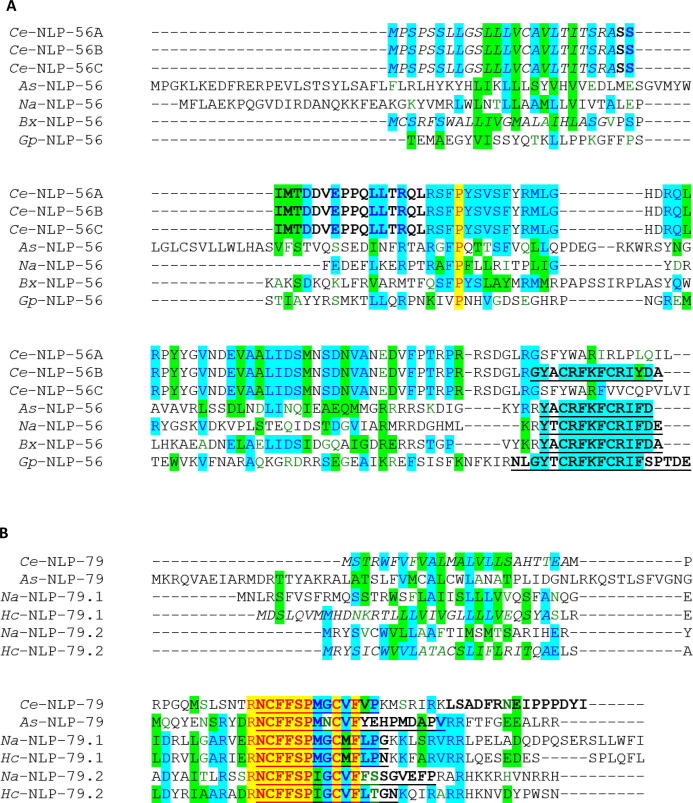
Novel predicted *Caenorhabditis elegans* neuropeptide-like proteins (*Ce*-NLPs) are conserved in parasitic nematodes. Multiple sequence alignments were performed using Vector NTI Advance 11.5 AlignX® (Lu and Moriyama, 2004). Text in italics highlights signal peptides as identified using Signal P 4.1 (Nielsen, 2017). Bold text indicates peptides predicted previously (see Van Bael et al., 2018). Bold underlined text indicates novel peptides predicted in this study based on their conservation in additional nematode species. Yellow and blue highlighted regions specify completely and partially conserved amino acid residues, respectively; green specifies similar residues. For all other novel NLP predictions made here (derived from *nlp*-8, -40, -48, -48, -52, -56, -58, -66, -67, -79 and -81 prepropeptide alignments) see Supplementary Table S5 and Supplementary Fig. S1. *Ce*, *C. elegans*; *As, Ascaris suum*; *Na, Necator americanus*; *Hc, Haemonchus contortus*; *Bx, Bursaphelenchus xylophilius*; *Gp, Globodera pallida*.

For the most part the conserved NLP motifs, identified through sequence alignments of putative prepropeptide nematode parasite NLP sequelogues, map to the previously predicted peptide regions identified via *C. elegans* in silico and mass spectrometry analyses (Husson et al., 2005, Van Bael et al., 2018). However, approximately one-third of the previously predicted NLP peptides, including multiple peptides identified by *C. elegans* mass spectrometry (Husson et al., 2005, Van Bael et al., 2018), are not conserved in any of the parasite species examined in this study (Supplementary Fig. S1; Supplementary Table S5). These peptides likely represent *C. elegans*-specific functionally important peptides or by-products of neuropeptide processing events. Indeed, sequence alignments of some parasite- and *Ce-*NLP prepropeptides (e.g. NLP-56, NLP-79; Fig. 1) reveal alternative highly conserved motifs, that were not previously considered as putative peptides (Van Bael et al., 2018), but may represent functional NLPs. These data are fundamental to the construction of the peptide libraries that seed deorphanisation efforts and underscore the value of pan-phylum in silico analyses for peptide prediction.

### 3.6 Parasitic nematode in silico analyses reveal novel NLP-encoding genes and highlight the need for nomenclature revision

Phylum-spanning in silico analyses of NLP-encoding genes drives novel peptide discovery in parasites and provides an opportunity for identification of additional *nlps* in *C. elegans*. This study discovered 14 novel *nlps* (Table 1), which have been designated *nlp*-85 to *nlp*-98. For continuity with previously identified *Ce*-*nlps* (Nathoo et al., 2001, McVeigh et al., 2008, Van Bael et al., 2018), and before naming any further novel *nlp*-genes identified here, we have reassigned *snet*-1 and *rgba*-1 as *nlp*-83 and -84, respectively. Although not classed as an *nlp* gene by the current version of WormBase (WS276), *snet*-1 (*nlp*-83) was identified as a *C. elegans* neuropeptide gene in an olfactory plasticity study and previously considered as a member of the NLP family (Yamada et al., 2010, Hobert, 2013); *rgba*-1 was identified as a novel *C. elegans* neuropeptide-encoding gene (Yin et al., 2017). The novel *nlps* discovered in this study include additional members of RPamides and Allatostatins, and a number of entirely novel *nlps* that had not previously been reported in *C. elegans*.

#### 3.6.1 Novel RPamide-encoding genes were identified in parasitic nematodes

The *C. elegans* RPamide family consists of the *nlp*-2, -22 and -23 gene cluster first described by Nathoo et al. (2001), plus *nlp-*46, identified by McVeigh et al. (2008). The NLP-2, -22 and 23 peptides are highly similar, making sequelogue designation difficult for returned BLAST hits using the *C. elegans* directed BLAST approach. We therefore extended our BLAST searches using all of the RPamide encoding BLAST returns to query all available nematode genomes, and performed multiple sequence alignments to manually identify patterns of sequence conservation. Of the known RPamide encoding genes, *nlp*-22 and -23 sequelogues were only identified in *Caenorhabditis* spp., whereas *nlp*-2 and *nlp*-46 were conserved in a number of parasites (Table 1, Supplementary Table S4).

Three novel RPamide-encoding genes were identified in parasitic nematodes: (i) a novel RPamide sequence A(A/V/T)MISGRGFRPG, initially identified here in *Brugia malayi* and *Dirofilaria immitis,* and sharing a common motif with RPamides encoded in 14 other filarial species, was classified as a novel filarid-specific RPamide-encoding gene (*nlp*-85); (ii) a second novel RPamide peptide-encoding gene (designated here as *nlp*-86; GRW(G/Q)LRPG), identified initially here in *A. suum*, *Bursaphelenchus xylophilius* and *Globodera pallida* was also present in 11 additional nematode species representing both free-living and parasitic lifestyles; finally (iii) a third novel *nlp (nlp-*87; S(I/L)ALGR(F/L)(S/N)LRPG), identified initially here in *A. suum*, and subsequently in seven additional nematode species appears distinct from peptides encoded on *nlp*-2 and *nlp*-86.

**Fig. 2:**
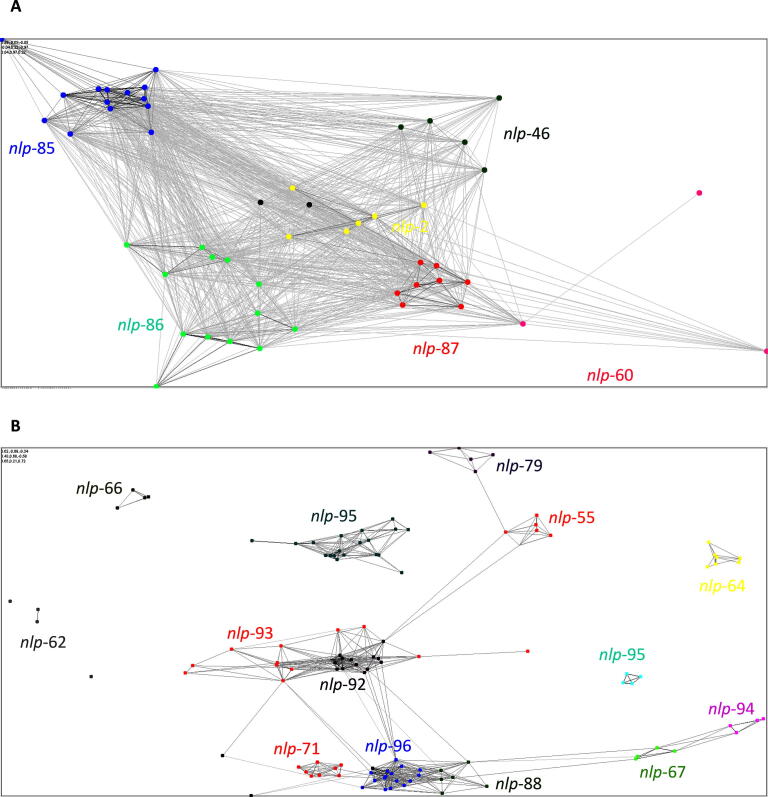
Clustered Analysis of Sequences (CLANS) clustering supports RPamide and Allatostatin-C like peptide encoding gene nomenclature. (A) Similarity matrix derived from all-against-all BLASTp comparisons between all identified nematode RPamide encoding prepropeptide sequences (*E*-value limit = 1). (B) Similarity matrix derived from all-against-all BLASTp comparisons between all identified nematode Allatostatin-C like prepropeptide sequences (*E*-value limit = 1E−5). *nlp*, neuropeptide-like protein.

The presence of *nlp*-2, -86, and -87, that encode distinct RPamide peptide motifs, in the *A. suum* genome and subsequent CLANS analysis provides evidence to support the novel *nlp* designations (Fig. 2A). However, several previous studies have been unable to accurately delineate the RPamide-encoding *nlps* such that there are conflicting designations in the literature to what we describe here (McVeigh et al., 2008, Jarecki et al., 2011, Nelson et al., 2013, Koziol et al., 2016). This underscores the value of phylum-spanning in silico analyses to unravel the complexity of the NLP family and associated nomenclature. In addition, our approach offers a route to interrogate neuropeptide evolution; indeed, both motif and CLANS analyses reveal that *nlp*-60 (a non-RPamide encoding gene) likely represents an RPamide family member that is closely related to *nlp*-87 but has lost all RPamide peptides (Fig. 2A).

#### 3.6.2 Novel Allatostatin-C-like peptide encoding genes were identified in *C. elegans* and parasitic nematodes

Our pan-phylum motif-based and reciprocal BLAST approach enabled us to identify nine novel Allatostatin-C-like neuropeptide encoding genes (*nlp*-88, -89, -90, 91, -92, -93, -94, -95 and -96), in addition to those previously reported (*nlp*-55, -62, -64, -66, -67, -71 and -79; (Mirabeau and Joly, 2013, Van Bael et al., 2018)).

Four of the novel Allatostatin-C-like neuropeptide encoding genes (*nlp*-88, -89, -90 and -91) were found in the *C. elegans* genome, share a common RNCFF(S/T)P(V/A)QC motif, possess a signal peptide, and appear to be enriched in neurons (see WormBase (WS276): WBGene00010848 (M04B2.6); WBGene00016436 (C35B1.7); WBGene00019292 (K02A6.1); WBGene00019293 (K02A6.1)). *nlp*-89, -90 and -91 appear to be restricted to Caenorhabditids, whereas *nlp*-88 is conserved across parasitic nematodes (Table 1; Supplementary Table S4). The additional five novel Allatostatin-C-like neuropeptide encoding genes identified here (*nlp*-92, -93, -94, -95 and -96) share a common (K/R)NC(F/Y)F motif, are conserved across key parasite species, but are absent from *C. elegans*. Again, CLANS analysis broadly supports the peptide motif based novel gene designations highlighted here (Fig. 2B).

#### 3.6.3 Novel NLP families are absent from *Caenorhabditis elegans*

Two additional novel putative NLP-encoding genes, that display distinct peptide motifs (NPYSW, and S(L/V)AP(T/S)TSAX_3-4_VS), were identified in this study and have been designated *nlp*-97 and -98, respectively; *nlp*-97 and -98 are absent from *C. elegans*. *nlp*-97 sequelogues were identified in a broad range of parasites, spanning clades 8, 9, 10 and 12 (Table 1), whilst *nlp-*98 appears to be *Globodera* spp. specific (see Supplementary Fig. S1). Note that *G. pallida* and *Globodera rostochiensis* each possess two highly similar *nlp-*98 sequelogues (*nlp-*98.1, -98.2); we also note recent *nlp* duplications in specific parasitic nematodes for *nlp*-1, -8, -10, -13, -15, -19, -21, -36, -40, -47, -61, -69, -71, -76 and -79 and have designated these paralogues as *nlp*-X.1, *nlp*-X.2 etc (see Supplementary Fig. S1).

### 3.7 Highly conserved NLPs are bioactive on the *Ascaris suum* ovijector

To determine the potential role of the most highly conserved NLPs in regulating muscle function, we examined the effects of the following predicted *A. suum* NLPs: *As*-PDF-1 (A and B), *As*-PDF-2 (C), *As*-NLP-12 (A1, B, C, D), NLP-36 (A1, A2, B1, B2, C1, C2), *As*-NLP-47 (A) and *As*-NLP-49 (B) (see Supplementary Table S6; Supplementary Table S7) on the reproductive musculature (ovijector) of *A. suum*, using established methods (Fellowes et al., 1998, Fellowes et al., 2000, Moffett et al., 2003). Note that NLP-12 (A1, B, C and D), and NLP-36 (A1, A2, C1 and C2) peptides were inactive (see Supplementary Table S6 ; Supplementary Table S7) .

**Fig. 3:**
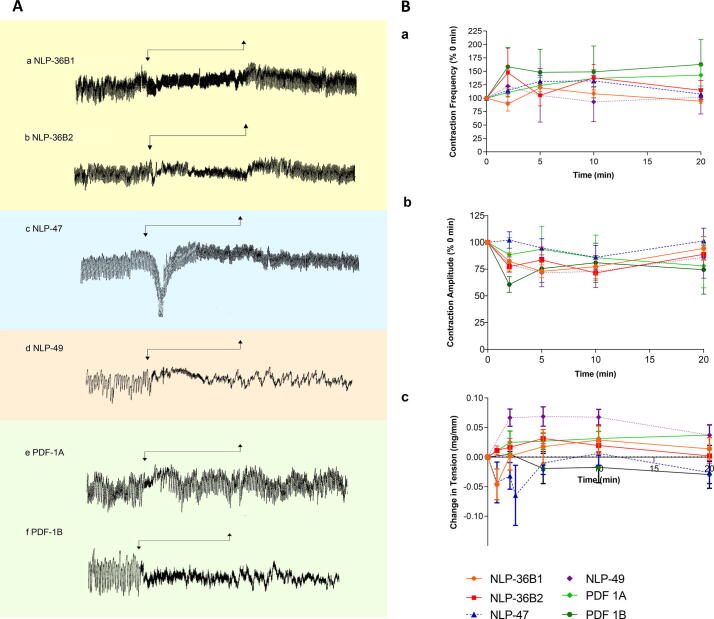
Selected neuropeptide-like protein predicted peptides (NLPs) are bioactive on the *Ascaris suum* ovijector muscle. (A) Representative muscle tension recordings showing the effect of *As*-NLP-36, *As*-NLP-47, *As*-NLP-49, and *As*-PDF-1 peptides. Arrows indicate peptide exposure. (B) The effects of NLPs on (i) *A. suum* ovijector contraction frequency, (ii) contraction amplitude, and (iii) baseline tension. Peptide exposure: 0–10 min. Scale: horizontal bar represents 1 min, vertical bar represents 1 mg.

NLP-36B1, -36B2, -47, PDF-1A, -1B peptides exhibited distinct excitatory effects on ovijector muscle (Fig. 3, Supplementary Table S6 ;Supplementary Table S7) as follows; (i) *As*-NLP-36B1 and *As*-NLP-36B2 induced an increase in contraction amplitude and frequency that aligned with the previously described ovijector response type (RT) 5 (Moffett et al., 2003); (ii) *As*-NLP-47 induced a transient lengthening of the ovijector with an increase in contraction frequency, similar to RT2 type responses, whilst (iii) *As*-PDF-1A and *As*-PDF-1B caused a decrease in contraction amplitude alongside an increase in contraction frequency that aligned to RT5. By contrast, *As*-NLP-49 was the only peptide tested shown to induce relaxation of the ovijector muscle (RT1; Fig. 3, Supplementary Table S6; Supplementary Table S7).

The muscle physiology described here represents the first known reports of NLP activity on the *A. suum* ovijector (Moffett et al., 2003, Mousley et al., 2004, Mousley et al., 2005, McVeigh et al., 2006b, McVeigh et al., 2008). Intriguingly, *pdf*-1, *nlp*-47 and *nlp*-49 are all linked to the control of egg-laying in *C. elegans* (Lindemans et al., 2009, Meelkop et al., 2012, Chew et al., 2018), a role which may be conserved in parasitic nematodes.

## 4 Conclusions

This study provides a comprehensive library of NLPs in nine key parasitic nematodes, and highlights that parasites possess a reduced and variable complement of the *C. elegans* NLP profile. We identify 14 novel *nlps*, 10 of which are not found in *C. elegans*, bringing the total number of *nlps* in nematodes to 99. Five *nlps* display complete conservation across the nine phylogenetically dispersed parasitic nematodes examined here; four of these encode peptides that modulate nematode muscle function.

Whilst this study has been successful in the identification of >300 *nlp* genes across key parasitic nematode species, there are caveats associated with BLAST-based identification of neuropeptide sequelogues. Indeed, the use of *C. elegans* NLP sequences as BLAST queries may not uncover all parasite sequelogues, particularly in the case of those NLPs which display divergent sequences. Enhancements in genome quality for any of these species may reveal additional neuropeptide sequelogues. Note that the non-identification of a specific gene using this BLAST-based approach does not definitively prove its absence from any given parasite. The value of peptidomics approaches, particularly de novo sequencing, to neuropeptide discovery is clear when we consider that many of the more recently identified *C. elegans* neuropeptides are not traditionally ‘*nlp-/flp*-like’ (Nathoo et al., 2001, Van Bael et al., 2018). *Caenorhabditis elegans* data show that highly conserved neuropeptides tend to be discovered first by in silico studies, however de novo peptidomics approaches have obvious advantages when sequences are divergent from search string motifs. Nevertheless, in silico studies are highly valuable as they support exploitation of the huge amount of available in silico data to expand our knowledge of invertebrate neuropeptide signalling.

The data presented here advance our understanding of neuropeptide signalling in parasitic nematodes, support the possibility for conservation of neuropeptide function across multiple nematode species, and inform neuropeptide-receptor deorphanisation studies in therapeutically relevant parasite species.

## Appendix A Supplementary data

Supplementary Fig. S1 Nematode neuropeptide-like protein (NLP) sequelogue alignments from Caenorhabditis elegans and parasitic nematodes. Multiple sequence alignments were performed using Vector NTI Advance 11.5 AlignX® (Lu and Moriyama, 2004). Italic text indicates the presence of a signal peptide as identified using Signal P 4.1 (Nielsen, 2017). Ce-NLP-56A, B and C represent isoforms of the C. elegans nlp-56 gene. Parasite sequelogues designated NLP-X.1, -X.2 etc. represent paralogues (likely recent gene duplications) of C. elegans NLPs in these species. Note: pan-phylum alignments are not an exhaustive list of all NLP-sequelogues across all nematodes. Ce, C. elegans; Ts, Trichinella spiralis; Tm, Trichuris muris; As, Ascaris suum; Bm, Brugia malayi; Di, Dirofilaria immitis; Na, Necator americanus; Hc, Haemonchus contortus; Bx, Bursaphelenchus xylophilius; Gp, Globodera pallida. Yellow and blue highlighted regions indicate completely and partially conserved amino acid residues, respectively. Green indicates similar residues. References: Lu, G., Moriyama, E.N., 2004. Vector NTI, a balanced all-in-one sequence analysis suite. Brief Bioinform, 5, 378-388. Nielsen H., 2017. Predicting Secretory Proteins with SignalP. Methods Mol Biol, 1611:59-73.

Supplementary Fig. S2 Expression Heatmaps for nematode neuropeptide-like protein (NLP) genes expressed in lifecycle stages of (A) Trichuris muris, (B) Ascaris suum, (C) Brugia malayi, (D) Dirofilaria immitis, (E) Haemonchus contortus, (F) Bursaphelenchus xylophilus, (G) Globodera pallida. (A) Expression heatmap generated from log2FPKM values of seven genes in T. muris identified from developmentally staged RNA-seq libraries and https://parasite.wormbase.org utilising the Expression program in Heatmapper (http://heatmapper.ca/expression; Babicki et al. (2016)). Average Clustering Method and Pearson’s Distance Measurement Method were used. Tissues and Life stages are represented in columns (L2, L3, Mixed Adults, Adult Female, Adult Male, Anterior, Female Rear, Male Rear). Rows indicate individual genes, denoted by the gene ID. nlp-6, nlp-12, nlp-47, nlp-58, pdf-1/nlp-74, snet-1/nlp-83 have been omitted as these genes are unannotated. (B) Expression heatmap generated from log2RPKM values of 43 genes in A. suum identified from developmentally staged RNA-seq libraries and https://parasite.wormbase.org utilising the Expression program in Heatmapper (http://heatmapper.ca/expression; Babicki et al. (2016)). Average Clustering Method and Pearson’s Distance Measurement Method were used. Samples are represented in columns (L3 and Egg, L3 Liver, L3 Lung, L4, Male Muscle, Female Muscle, Male Rear, Female Rear). Rows indicate individual genes, denoted by the gene ID. nlp-9, nlp-19, nlp-35, nlp-37, nlp-42, nlp-46, nlp-49, nlp-51, nlp-57, nlp-69, nlp-71, ntc-1/nlp-75, nlp-76, nlp-87, nlp-92, nlp-93, nlp-95 have been omitted as these genes are unannotated. (C) Expression heatmap generated from log2TPM values of 29 genes in B. malayi identified from developmentally staged RNA-seq libraries and https://parasite.wormbase.org utilising the Expression program in Heatmapper (http://heatmapper.ca/expression; Babicki et al. (2016)). Average Clustering Method and Pearson’s Distance Measurement Method were used. Life stages are represented in columns (Eggs and Embryos, Microfilariae, L3, L4, Adult Female, Adult Male). Rows indicate individual genes, denoted by the gene ID. nlp-64 and nlp-71 have been omitted as these genes are unannotated. (D) Expression heatmap generated from log2FPKM values of 18 genes in D. immitis identified from developmentally staged RNA-seq libraries and https://parasite.wormbase.org utilising the Expression program in Heatmapper [http://heatmapper.ca/expression; Babicki et al. (2016)]. Average Clustering Method and Pearson’s Distance Measurement Method were used. Life stages and tissues are represented in columns (Microfilariae, L3, L4, Adult Male, Adult Female, Female Body Wall, Uterus, Female Intestine, Female Head, Male Intestine, Male Body Wall, Testes). Rows indicate individual genes, denoted by the gene ID. nlp-12, nlp-37, pdf-1/nlp-74 and nlp-81 have been omitted as these genes are unannotated. (E) Expression heatmap generated from log2FPKM values of 57 genes in H. contortus identified from developmentally staged RNA-seq libraries and https://parasite.wormbase.org utilising the Expression program in Heatmapper (http://heatmapper.ca/expression; Babicki et al. (2016)). Average Clustering Method and Pearson’s Distance Measurement Method were used. Samples are represented in columns (Egg, L1, L3, L4, Adult Female, Adult Male, Adult Male Gut). Rows indicate individual genes, denoted by the gene ID. nlp-9, nlp-11, nlp-47, nlp-50, nlp-57, nlp-59, nlp-60, nlp-65, nlp-71, pdf-1/nlp-74, ntc-1/nlp-75, nlp-81 and nlp-92 have been omitted as these genes are unannotated. nlp-18 has been omitted as no expression data is available. (F) Expression heatmap generated from log2FPKM values of 48 genes in B. xylophilus identified from developmentally staged RNA-seq libraries and https://parasite.wormbase.org utilising the Expression program in Heatmapper (http://heatmapper.ca/expression; Babicki et al. (2016)). Average Clustering Method and Pearson’s Distance Measurement Method were used. Life stages are represented in columns (L2+3h, L2+6h, L2 mixed, Stage 3 Dispersal Juvenile, L3 mixed, L4 mixed, Adult Female, Adult Male, Dauer, Mixed Embryo, Mixed Propagative). Rows indicate individual genes, denoted by the gene ID. nlp-69 has been omitted as this gene is unannotated. (G) Expression heatmap generated from log2TPM values of 38 genes in G. pallida identified from developmentally staged RNA-seq libraries and https://parasite.wormbase.org utilising the Expression program in Heatmapper (http://heatmapper.ca/expression; Babicki et al. (2016)). Average Clustering Method and Pearson’s Distance Measurement Method were used. Life stages are represented in columns (Egg, J2, 7 days p.i. (d.p.i.), 14 days p.i., 21 days p.i., 28 days p.i., 35 days p.i., Adult Male). Rows indicate individual genes, denoted by the gene ID. nlp-15, nlp-19, nlp-56, nlp-69, nlp-72 have been omitted as these genes are unannotated.
