# Supplementary material for: In silico analyses of neuropeptide-like protein (NLP) profiles in parasitic nematodes
Source: Int J Parasitol. 2022 Jan;52(1):77–85. doi: 10.1016/j.ijpara.2021.07.002 (PMC8764417; doi:10.1016/j.ijpara.2021.07.002)
Supplement: Supplementary Fig. S1 — Nematode neuropeptide-like protein (NLP) sequelogue alignments from Caenorhabditis elegans and parasitic nematodes. Multiple sequence alignments were performed using Vector NTI Advance 11.5 AlignX® (Lu and Moriyama, 2004). Italic text indicates the presence of a signal peptide as identified using Signal P 4.1 (Nielsen, 2017). Ce-NLP-56A, B and C represent isoforms of the C. elegans nlp-56 gene. Parasite sequelogues designated NLP-X.1, -X.2 etc. represent paralogues (likely recent gene duplications) of C. elegans NLPs in these species. Note: pan-phylum alignments are not an exhaustive list of all NLP-sequelogues across all nematodes. Ce, C. elegans; Ts, Trichinella spiralis; Tm, Trichuris muris; As, Ascaris suum; Bm, Brugia malayi; Di, Dirofilaria immitis; Na, Necator americanus; Hc, Haemonchus contortus; Bx, Bursaphelenchus xylophilius; Gp, Globodera pallida. Yellow and blue highlighted regions indicate completely and partially conserved amino acid residues, respectively. Green indicates similar residues. References: Lu, G., Moriyama, E.N., 2004. Vector NTI, a balanced all-in-one sequence analysis suite. Brief Bioinform, 5, 378-388. Nielsen H., 2017. Predicting Secretory Proteins with SignalP. Methods Mol Biol, 1611:59-73. [file mmc1.docx]

NLP-1

1 60

Ce-NLP-1 (1) *MKATFVLACLLVIAAVSHA*DLLPKRMDANAFRMSFGKRSVSNPAEAKRMDPNAFRMSFGK

As-NLP-1 (1) ---------------MEVKLKKENNRFFFNFIKRYLERVIFHRAFGKRMDASKFYMGFGK

Na-NLP-1 (1) ------------------------------------------------------------

Hc-NLP-1.1 (1) -----------------------------------------------------------*M*

Hc-NLP-1.2 (1) ----------------------------------------------------------*-M*

Gp-NLP-1.1 (1) -----------------------------------------------------------*M*

Gp-NLP-1.2 (1) ---------------------------------------------------------*MAS*

Gp-NLP-1.3 (1) ------------------------------------------------*MSPNLFIVFSLL*

61 120

Ce-NLP-1 (61) RSAEQNEQANKEDKATSDKLYDDTKFEEMKRMDANAFRMSFGKRSDAHQAADDQVEYVND

As-NLP-1 (46) RDGYIRRLPEINRTIIDSEVHLQFSGKAAKEDGCNSEEFSLNAKATS--LAR-------H

Na-NLP-1 (1) ----------MVIVAALSQDYDEFIGGKRAAMDPHAFRMSFG------------------

Hc-NLP-1.1 (2) *NNFRLTVLSFMVVVAVFS*QEFEVFTEGKQAEMYPRALPFRMSRG----------------

Hc-NLP-1.2 (2) *DNLRITILSLMIVVAVFT*QEYEVFTEGKRAVMFPRTFGALFG------------------

Gp-NLP-1.1 (2) *SRKFELFLAITLLFMAQSNK*RSFVALAELQNQDRMAQIRTLP------------------

Gp-NLP-1.2 (4) *FQYFAVILFSLLILRLITLNEVRA*APTGGLQLDRSAFRMSFGKRSP--AAAAESDSSELN

Gp-NLP-1.3 (13) *LLAVPLRLANA*EPEFVMAKKETEVG-PTGFQMDRNAFRMSFGKRDEALVPFRMLR-----

121 180

Ce-NLP-1 (121) DFSLPEQKRMDANAFRMSFGKRVNL-------------------------DPNSFRMSFG

As-NLP-1 (97) IYEPLMEKRMDASRFYLGFGKRADEQAPYG----------N-ILLNLLDSERLETEIAVD

Na-NLP-1 (33) --KRFVGRNIDPNAFR--MSFGKRS-------------------------ASPQLEIVNP

Hc-NLP-1.1 (46) --KRSTGIRFDPNASRTRMSFGKR--------------------------NAYGSNEWNE

Hc-NLP-1.2 (44) --KRLTGNRIGLNTFR--MGSGIQ--------------------------KAYGINDWNE

Gp-NLP-1.1 (44) --SVG--LQMDPNVFQIGFGKREQQ-------------------------QNVQQPMEAF

Gp-NLP-1.2 (62) NANIAIPLMYDMSEAETTMKQQQQP---------------R-HKVFPSGGVGRAPWPSRW

Gp-NLP-1.3 (67) --SNPRQLAMDRNAFRMSFGKRSPAGSSSSNELINANIDANHQQKYRAFWSPPAANGWEA

181 203

Ce-NLP-1 (156) KRSTVGYNLDARNYFVGLGRR--

As-NLP-1 (146) GVVKSPSELEPGVHTNHTLKKSA

Na-NLP-1 (64) YDQELVKRMDMKHYYIGLGKR--

Hc-NLP-1.1 (78) YLKENTKRMDMRHDFVGLGNR--

Hc-NLP-1.2 (74) YIKDNDKRMDMKHYFVGLGRR--

Gp-NLP-1.1 (75) LLDRNVFRMSFGKRELVKN----

Gp-NLP-1.2 (106) KASLFSKRLDRNLYNIGFGR---

Gp-NLP-1.3 (125) AAALPPKRLDRNLFNVRFGRRRR

NLP-2

1 50

Ce-NLP-2 (1) ---------------------------------------*MRATLVLFALL*

As-NLP-2 (1) --------------------------------------------------

Na-NLP-2 (1) MERKSCEVNNIAIFDYVDDDDVLAYKQVEVNSPFELGGYENEVNGLEPYI

Bx-NLP-2 (1) ----------------------------------------MARTIFGLFF

51 100

Ce-NLP-2 (12) *CAVYSEA*VPLQVYRPDESSAVDVVVLENSPELYDSEDEDEWKQEEEFTEG

As-NLP-2 (1) -----------RYDLRFSNFVFKAISESE--FPAELQKRSMALGRLAFR-

Na-NLP-2 (51) YIRYNCDKPSYRSIMFRMTFIASLMLRNKSWRADMLPLVGFLTISAIWVV

Bx-NLP-2 (11) GPNKVAKSTVTACRCADRSVIRQTNIVQLRARAPANPFSKEMWPRIMMTF

101 150

Ce-NLP-2 (62) AMGKRSIALGRSGFRPGKR---SMDNFHTVDVSDLIMKRSMAMGRLGLRP

As-NLP-2 (37) -PGKRSLALGRVDFRPGKRNVDIADKDIEYLMEDSMGKRSAAFGRFHFRP

Na-NLP-2 (101) AWAGLTSPLGASLGNESPREILSVLPLHVVTRRPMDEFSPRVRTRLGRYK

Bx-NLP-2 (61) AFTSMVMLIEAAPHTAGRN---AEDGEVVLMAAENPNARYLYMQPQEQVE

151 200

Ce-NLP-2 (109) GKRSMAYGRQ----GFRPGKRSMAYGRQGFRPGKRSMAYGRQG-------

As-NLP-2 (86) GKRSLALGRS----GFRPGKRSLALGRVGFRPGKRASGEVLIDPCSIEID

Na-NLP-2 (151) PEDDVSNETQRFFTEERMGKRSIALGRSGFRPGKRSDSLESHG-------

Bx-NLP-2 (108) PDWAEVFSQP----DPRMEKRSVALSRLNFRPGKRSVPLATQADLVEQG-

201 229

Ce-NLP-2 (148) ---------FRPGKRSNDMKEVFPQHVPE

As-NLP-2 (132) SLWGDMYDIAQVGMRSNFSREAFYYC---

Na-NLP-2 (194) ---------LRPGRGSFDEM---------

Bx-NLP-2 (153) -----IYRLNALQKRSVAISRKGFRPAKK

NLP-3

1 40

Ce-NLP-3 (1) *MSKIVACLVLLALSVMCVYS*APYEFRAKR-----AINPFL

As-NLP-3 (1) ---MQPLSVTAQHTGLATHAPPTNAPFPL-----LLYFAL

Bm-NLP-3 (1) *MTTAICLLMV---MILLSYRTES*QYVSRQGNYIDYNRGDL

Di-NLP-3 (1) *MTKATCLLMVIWSMILLAYRTES*QYASRRGFYVDHNRGDL

Na-NLP-3 (1) --*MLKAVFLSLLVLVSIVFTAYA*DMRAKR-----AINPFL

Hc-NLP-3 (1) --*MLKGMVFSLLVFVTLLFSAYSDTRA*KR-----AINPFL

Bx-NLP-3 (1) *MSPFRGVSLASVLFAVLFLATTLSA*FLQDEVP---LITEI

Gp-NLP-3 (1) *MFSSSTLPVLLQLVAVIFISNPSEA*YSAG----RLYLRDS

41 80

Ce-NLP-3 (36) DSMGKRAVNPFLDSIGKR-----------SFRPDMITEEK

As-NLP-3 (33) FARFRRAINPFTDSIGKRN----------TGIAAERDLPK

Bm-NLP-3 (38) YMRNFGIINPFSDSDSRR-----------IRFDSSRELPK

Di-NLP-3 (41) YMRNFGVINPFSDADSKR-----------IRFDNSRELPK

Na-NLP-3 (34) DSMGKRALNPFLDSIGKRA----------SQYQPYYGYEK

Hc-NLP-3 (34) DSMGKRAVNPFLDSFGKRS----------SRYQPYYHLDK

Bx-NLP-3 (38) EGVAKRAINPFMDSIGKRSGNVYVPGSRFYRHSLLFQRPS

Gp-NLP-3 (37) YPDSARFFIPNALLADQLLASGPVSSLRPALFDRSPIPAK

81 120

Ce-NLP-3 (65) RYFDSLAGQSLGKRSNNRYEMLENYY--------------

As-NLP-3 (63) RYFDALAGQSLGKRASPIYYLVDE----------------

Bm-NLP-3 (67) RYFDALAGQSLGKRSTTIL---------------------

Di-NLP-3 (70) RYFDALAGQSLGKRSATIL---------------------

Na-NLP-3 (64) RYFDSLAGQSLGKRSIQTYDA-------------------

Hc-NLP-3 (64) RYFDSLAGQALGKRSIQAYDA-------------------

Bx-NLP-3 (78) RYFDSLAGQSLGRR--------------------------

Gp-NLP-3 (77) RYFDSLAGQSLGKRTAPPATEEEMPRGDDARVNSQRAMRL

121

Ce-NLP-3 (91) ----------

As-NLP-3 (87) ----------

Bm-NLP-3 (86) ----------

Di-NLP-3 (89) ----------

Na-NLP-3 (85) ----------

Hc-NLP-3 (85) ----------

Bx-NLP-3 (92) ----------

Gp-NLP-3 (117) DALRPGWPMW

NLP-5

1 50

Ce-NLP-5 (1) --------------------------------------------------

Ts-NLP-5 (1) MVHHNWESHKWTTSFERCDQFLPDEPTVEPELAKIRHQVVSPLTDGIDLF

Tm-NLP-5 (1) --------------------------------------------------

Na-NLP-5 (1) --------------------------------------------------

Hc-NLP-5 (1) --------------------------------------------------

Bx-NLP-5 (1) --------------------------------------------------

51 100

Ce-NLP-5 (1) ------------------------------------------------ML

Ts-NLP-5 (51) TWQLSAFIAPLTSSFSSTYCRQYKRFPPNARLPLEATSLCTVVHFLQSSK

Tm-NLP-5 (1) --------------------------------------------------

Na-NLP-5 (1) -------------------------------------------*MYVVVLL*

Hc-NLP-5 (1) ------------------------------------------*MYIILFSC*

Bx-NLP-5 (1) --------------------------------------------------

101 150

Ce-NLP-5 (3) *MKIMVLMGIANIAAS*FVVSSVRFQSAPMRALIEINRELAKRSVSQLN---

Ts-NLP-5 (101) HTMVLKATLNGLLLFWLMISTQLISTLTTARFIYNIPSDTKSLSDRTFEK

Tm-NLP-5 (1) --MSALRGSPRALLLAIIMSTQLISNVCLGRYLRVYPEELRALRNFDGDS

Na-NLP-5 (8) *FVVAQTACS*TENLDTSEHDKRALSSFDTLAGIGLGKRAQLSSIDSLAGLG

Hc-NLP-5 (9) *LVAAQTVLS*ETAGFDLTGDKRALSSFDTLGGIGLGKRTQLSSIDSLGGLG

Bx-NLP-5 (1) ----*MLGIYVNLLLLTVLASA*EIIPSNVLHKRQAKMEIKKDNLGYSD---

151 200

Ce-NLP-5 (50) ----QYAGFDTLGGMGLGKRSEPDQAGEKRALSTFDSLGGMGLGKRSSSS

Ts-NLP-5 (151) R-LPTQLYFDPLGGMALGKRAG----PSAISGQYFDPLAGMAFGKRAG-L

Tm-NLP-5 (49) HRLVIRQYFDPLGGMALGKRAS-DLDRIGSRLQFFDPLGGMALGKRSP--

Na-NLP-5 (58) LGKRALSSFDTLGGIGLGKRSF---VVVKKAVSSFDTLGGIGLGKRSE--

Hc-NLP-5 (59) LGKRALSSFDTLGGIGLGKRSE---DTAKKALSSFDTLGGIGLGKRDD--

Bx-NLP-5 (44) ----EMFNIDTLAGIGLGKRSS--PTPTRPRYALIVNLEDLVEGLDRS--

201 250

Ce-NLP-5 (96) SRVFVYDKRALQHFSSLDTLGGMGFGRK----------------------

Ts-NLP-5 (195) IRPTWNAGFPEPALNSSHYLDMNVSSVDLSTLNNPL--------------

Tm-NLP-5 (96) -----------PAKFHPDALDGALWNSPFAR-------------------

Na-NLP-5 (103) --LFRGDK---KSLSSFDTLAGIGLGKRSRPLTQQYYIPFRGIMVSANRK

Hc-NLP-5 (104) --MLAGEK---KSVSSFDTLAGIGLGKRSRLFSTYYYLPYRDSLEDMDQN

Bx-NLP-5 (86) ------AQ---KVRARFNRFHEI---------------------------

251

Ce-NLP-5 (124) ---

Ts-NLP-5 (231) ---

Tm-NLP-5 (116) ---

Na-NLP-5 (148) EQD

Hc-NLP-5 (149) VQE

Bx-NLP-5 (100) ---

NLP-6

1 55

Ce-NLP-6 (1) -------------------------------------------------------

Ts-NLP-6 (1) -------------------------------------------------------

Tm-NLP-6 (1) -------------------------------------------------------

Na-NLP-6 (1) MAVSKFFSSFGVQRFQDILLREFVDIEIFTDLAECCTNASNPYDGCIHSCKHRRR

Bx-NLP-6 (1) -------------------------------------------------------

56 110

Ce-NLP-6 (1) -------------------------------*MLSFSRLAFVLLVSACVMAMA*APK

Ts-NLP-6 (1) -----------------------------------*MNIFITLFAFIVVMQQFSVD*

Tm-NLP-6 (1) -----------------------------*MAATVLPKVYSALLCLFVIVHS*FASF

Na-NLP-6 (56) QNPEKFSLQVFPVRFHSLRKSHGCALTLSVKMARITRFLVCLLLAVVGLLNASPK

Bx-NLP-6 (1) -----------------------------*MHSLVITTLLLAAISNSVIG*LGYIYE

111 165

Ce-NLP-6 (25) QMVFGFGKRS------MADISDMPEDVPMKRYKPRSFAMGFGKRAAMRSFNMGFG

Ts-NLP-6 (21) *S*NELSNNGRR--------IHFQNYFQRMQKRLDPQYFQLGFGKRFIVNEENYPTN

Tm-NLP-6 (27) EPMLIMAGAQ----P---LWKAPRSSSAKRPFDPYHFQMGFGKRVASYAAQDQPF

Na-NLP-6 (111) QHVFGFGKRG----GEALGDPEVDWNAMSKRLSNGKFMMGFGKRGLNKQFTMGFG

Bx-NLP-6 (27) DAYYPIPRGSQIVV--EAPSIPDEKVIPQRRFDPAWRHIGLGKRTAEERRTVSGE

166 220

Ce-NLP-6 (74) KRSAEPQIDYEDMSADLEEIPVFIQK----------------RRLIMGLGRR---

Ts-NLP-6 (68) MQCCAQLQLLAAMEDGF--------------------------------------

Tm-NLP-6 (75) SVVKLCADVDNGRTLMKNCRHYVIAE-----------------------------

Na-NLP-6 (162) KRVPADEELLSEDYIDQSGRTSLLTKGLGKRLYGLNDLPGLRTIQYMGLGKRSVL

Bx-NLP-6 (80) RSWAMIGLGR---------------------------------------------

221 238

Ce-NLP-6 (110) ------------------

Ts-NLP-6 (85) ------------------

Tm-NLP-6 (101) ------------------

Na-NLP-6 (217) SREVAANLRRFNLGLGRR

Bx-NLP-6 (90) ------------------

NLP-7

1 50

Ce-NLP-7 (1) ---------*MYIKAALLIVVLFGVASQITS-------------------A*

As-NLP-7 (1) *MSYFSPLSAVAIILYASSVMS*SSMFRSDDS------------------HA

Na-NLP-7 (1) ---------*MIFLYLLLGVVSA*SVLQYDPR---------------YLMSH

Hc-NLP-7 (1) ---------*MIFVYVCLGVVSA*SVLHYEPRTVLHPLPSKRLVIPSYLSSH

Bx-NLP-7 (1) ------*MMRSLILFLFGLLMPVLLA*KSEPS--------------------

51 100

Ce-NLP-7 (23) LYLKQADFDDPRMFTSSFGKRSAIESEPQAYPKSYRAIRIQRRS------

As-NLP-7 (33) YHNSFLTSGMPVELIAAENEGNPARFNHIQGAASTKNKRFADEW------

Na-NLP-7 (27) YDKRTLDFDDPRLFSSAFGKRSGFPARSLS-FQIVKRSPYFGS-------

Hc-NLP-7 (42) YDKRTLDFDDPRLFSTAFGKRNGLVTTTLNRPRFIKRSPFLGTNVGLAYI

Bx-NLP-7 (25) -AG-GVYEFNPRDWKADMSPHNFYRFRRYN--------------------

101 150

Ce-NLP-7 (67) MDDLDDPRLMTMSFGKRMILPSLADLHRYTMYDKRG---SDIDDPRYFLF

As-NLP-7 (77) --D--DPRFLSSAFGKRSGMMEFDDPRFLSSAFGKRNHLIGFDDPRLFSS

Na-NLP-7 (69) -DD--S-----EDTYEKRDGMELMDPRFFTNSFGKR-SDFLFDDPRFSSV

Hc-NLP-7 (92) KRADMDPRFISNSFGKRSTLYDFDDPRFASLSFGKR-SGFDFDDPRFSSM

Bx-NLP-7 (53) -----------VFSNDKRSEMEFDDPRYFSTAFGKRASSFL---------

151 200

Ce-NLP-7 (114) SNRLTCRC------------------------------------------

As-NLP-7 (123) SYGKR----------ALFELQKKYDAAFDDPRFLSLAFGKK---------

Na-NLP-7 (110) SFGKRR--------------------------------------------

Hc-NLP-7 (141) SFGKRSGFDLEDPRFASMSFGKRSGFNFEDPRFASLSFGKRSGFDLDDPR

Bx-NLP-7 (83) --------------------------------------------------

201 228

Ce-NLP-7 (122) ----------------------------

As-NLP-7 (154) ----------------------------

Na-NLP-7 (116) ----------------------------

Hc-NLP-7 (191) FASMSFGKRSGSDLEDPRYWSMSFGKRR

Bx-NLP-7 (83) ----------------------------

NLP-8

1 55

Ce-NLP-8 (1) --------------------------------MSQKLLPISPLQLLFLQCLLIGF

As-NLP-8 (1) MKTVSLPNRLLKACTGGRILRSKRAFDRLDESALGLFRKRSFDRIDGSAFGPHRH

Na-NLP-8 (1) ----------------------------------*MAGHTSTFMRCLLVKFAVLAV*

Hc-NLP-8 (1) -----------------------------------*MTQYSSLLRLHLVQMVFLAL*

Bx-NLP-8 (1) -----------------------------------MTDWAGGERVPKTGGALGEH

Gp-NLP-8.1 (1) -----------------------------------*MTSKGALLSFVLTLLTVDA*Y

Gp-NLP-8.2 (1) --------------------------------MAFRYSEAIFITIQLFNFFLLTN

56 110

Ce-NLP-8 (24) TAAYPYLIFPASPSSGDSRR--------LVKRAFDRFDNSGVFSFGAKRFDR---

As-NLP-8 (56) KKAFDRIDGSDFGLVKRAFDRIEGAGFGLSKRAFDRIEGS-GFGLDKRAFDRLEG

Na-NLP-8 (22) *CSA*YPYLLDPDYELSDVIVS----------KRAFDRLEGN-EFGLLKRALN----

Hc-NLP-8 (21) *CSA*YPYVLNSDYELTDVAERPFG----GLTKRAFDRIESN-DFGLFKRSAA----

Bx-NLP-8 (21) KILGKVMRTIFTVLTVCFLQVLCLS---QGQNTEDRSREQ-------R-------

Gp-NLP-8.1 (21) PAFYMVRNLPQLQHPQNALQ----------SSELSRLRPS---------------

Gp-NLP-8.2 (24) AMNMHDFLWASAVRPAQSSQ---------NSALTDTLSYETNKSPAKLLMTVP--

111 165

Ce-NLP-8 (68) ---------YDDETAYGYGFD-NHIFKRSADP-YRFMSVPTKKAFDRMDN-----

As-NLP-8 (110) SDFGLVKRAFDRLEGSDFGLD-KRAFDRVGESNFGFDKRSFDRVADSTAFRLSRG

Na-NLP-8 (62) ------KRAFDRLDMADFGLRRKRAFDRIARAEFGLTGLRRKRSSDLSTSNEE--

Hc-NLP-8 (67) ------KRAFDRIEMADFGFRRKRAFDRVGRTEFGFEGVLRKRAADRLAD-----

Bx-NLP-8 (59) --YVLHKRAFDRVDLSPFDFG---AYSKRYNNDLRSLYRKKKSFDRLDQGPFG--

Gp-NLP-8.1 (51) ------KRAFDRLDASPFDFD--SISKRFSDDELAAMPLNDLYLSSPYAFG----

Gp-NLP-8.2 (68) -----FKRAFDRLDVSNFDFN-----KRFMEVLDVAPPPRSKLLFNIHRMPSELP

166 220

Ce-NLP-8 (107) --------------------SDFFGAKRKRSFDRMGGTEFGLMKRSAPE-----S

As-NLP-8 (164) AFSRIDDNDFGLFKLNAVNPGRRIHLLQKRPFDRIERSEFGLSKRFDSSALPAVR

Na-NLP-8 (109) -----------------------FGLRRRRALDRIGGSEFGLIKRSVDPHL---S

Hc-NLP-8 (111) -----------------------IGFRNKRGLDQLDGTDLGLMFDPVRP-----A

Bx-NLP-8 (107) ------------------------LVKRKRVFDRVDAGFGFGKRSSEMAVPGRIS

Gp-NLP-8.1 (94) ------------------------PF-RKRSFDRLEESAFFGQKRKRSFGP----

Gp-NLP-8.2 (113) --------------------------NKRKLAEGLKRRRWAMQAKQQLQAAG---

221 262

Ce-NLP-8 (137) REQLINNLAESIITLRRAREAESSPESQRTIITYDD------

As-NLP-8 (219) YVEINPELVDLLPLDDVVYSFVKQDVVPFNSFTIADEIPRKR

Na-NLP-8 (138) REELINDLADSIVSLHEDSALHGPKLVVFPVPAEERESNR--

Hc-NLP-8 (138) REELIDRLAYTIAAMG-----HATPVAISAVPLVDDQK----

Bx-NLP-8 (138) LSDLVSQAQSIEPID---------------------------

Gp-NLP-8.1 (120) -ERILRDN----------------------------------

Gp-NLP-8.2 (139) RGPTVSRVLGFGLSFGGMRRLRRTMLK---------------

NLP-9

1 50

Ce-NLP-9 (1) ------*MDRFATRFIALLLVLLQIGSIFA*TPIAEAQGAPEDVDDRRELEK

As-NLP-9 (1) ---------------------------------------IGFIWRRLIQK

Bm-NLP-9 (1) *MLTMKLFLHCAIFYLLTYA*HDINDDDKERTPILNNPEAEDLERQLFWTRK

Di-NLP-9 (1) ----------------------------------------------MMKK

Na-NLP-9 (1) ----------------MKNSCESVSGSLQNVFTDSGNSVRDARSWTIVAK

Hc-NLP-9 (1) -----------------*MPSSSTYAALLTIVLLVCLSAA*QNSENYPSLAK

51 100

Ce-NLP-9 (45) RGGARAFYGFYN-----AGN-SKRDQAAALPYYLYEKRGGGR-----AFN

As-NLP-9 (12) RAGARSFGPRIYDVESYLYP-TNKRFGSFSPYYFYQQPK-----------

Bm-NLP-9 (51) RAGARAFGSMRGNNFENLFFPSNKRHQTFSPYYYYQQLK-----------

Di-NLP-9 (5) RGGARAFGPMRSNNYEHLFVPNIKRHQTLSPYYYYYQQPK----------

Na-NLP-9 (35) RGGARAFHSYFN-----VPN--AKRLSVAAPYYLYEKRGGGRAFVGGWQP

Hc-NLP-9 (34) RGGARAFHGYFN-----MPS--SKRLSGEYPYYLYEKRGGGRAFFGGWQP

101 150

Ce-NLP-9 (84) HNANLFRFDKRGGGRAFAGSWS-------PYLERFYDYKRSSYPVYFSDN

As-NLP-9 (50) ----------RGGGRTFASYWIPPSGNGRSLLFGNIFFI-----------

Bm-NLP-9 (90) ----------RGGGHPFYTYWSPSNAGNVKFVEGSPFYKKRSVLRDSDDY

Di-NLP-9 (45) ----------RGGGHPFYTYLS-ENGGDGKFIEHLPFYKKRSLMHDSDDY

Na-NLP-9 (78) FDGISGRMERRGGGRAFSGWWG-PNEYFSRFYENPYYKRSSNLWEFLEDR

Hc-NLP-9 (77) YESLGARMDRRGGGRAFSGWLG-PNEYY-RFYENPYYKRSSSLWEFLEDR

151

Ce-NLP-9 (127) SYY

As-NLP-9 (79) ---

Bm-NLP-9 (130) IY-

Di-NLP-9 (84) AY-

Na-NLP-9 (127) NGF

Hc-NLP-9 (125) NAL

NLP-10

1 45

Ce-NLP-10 (1) ---------------------------------------*MWYIAL*

As-NLP-10 (1) ---------------------------------------------

Bm-NLP-10 (1) ---------------------------------------------

Di-NLP-10 (1) MINSHLLPLIIHARAGSNRRPSACKADVITTTPLDRCSVTDHFFN

Na-NLP-10 (1) ---------------------------------------------

Hc-NLP-10.1 (1) ---------------------------------------------

Hc-NLP-10.2 (1) -----------------------------------*MLQDVLVMLE*

Bx-NLP-10 (1) ---------------------------------------------

Gp-NLP-10 (1) ---------------------------------------------

46 90

Ce-NLP-10 (7) *LLAVIATSVTA*QKADDEPIVFLVRVPIDEMDDDSSLLESYYHP--

As-NLP-10 (1) -----------------MKNILDVLPFSGGLYG------------

Bm-NLP-10 (1) ---------------------------------------------

Di-NLP-10 (46) VSKISSKRCPNFIGNINQNIDLFCIKREIRNYYVFCFISTKNSLR

Na-NLP-10 (1) -------------*MLVPSLLFLVAVAYA*NEEEESSSSSDVVLLDL

Hc-NLP-10.1 (1) -*MDCFGKGNPLETMIVPSLLFLVAVAYA*NEDEGMYGKRSEMPDDM

Hc-NLP-10.2 (11) *SIPQLTTGNPLETMIVPSLLFLVAVAYA*NEDEDLASSS-------

Bx-NLP-10 (1) ----------*MRTVILCLILSVGCIGMASA*ASIYIDSN-------

Gp-NLP-10 (1) ----*MSSATISPLLLLPVLLVLLSVFGCHA*TPPSQHSQTEEN---

91 135

Ce-NLP-10 (50) -----------RDILSKRAIPFNGGMYGKR-STMPFSGGMYGKRS

As-NLP-10 (17) ---------------KRSSIPFHGGIYGKRTAALPFSG---G---

Bm-NLP-10 (1) ----------------*MVAILSEFLIYIILIYKIISA*KEVFDGEV

Di-NLP-10 (91) LVIGNTFNAKFNLRTVMVAILSEFLIYAILIHKITSANEAFDKKI

Na-NLP-10 (33) SDYAPGINRGLRFDIKRAAMPFSGGMYGKR-AVMPFSGGMYGKRD

Hc-NLP-10.1 (45) YIERPVLPLSAGWQEKRAVMPFSGGLYGKR-AVMPFSG-------

Hc-NLP-10.2 (49) -------------EAIMIVPDYAPEILGR-----AFNN-------

Bx-NLP-10 (29) ---------------RPDYEENLQKVYDNRLQPYAIEY-------

Gp-NLP-10 (39) -------------VLLEELVREVRKLSNRMDSALGRGGAAEEEEL

136 180

Ce-NLP-10 (83) G------QIFAQRRAAIPFSGGMYGKRS---LVPQSYSNNENQIK

As-NLP-10 (41) --------LYGKRAGWIPFSGGLYGKRA---LTPLQGGIYG---K

Bm-NLP-10 (30) QNVG--GGEAQKNDYPLPLSSNLYGKRVNYVLMPLMGGMYG---K

Di-NLP-10 (136) RNG---FGHVENDEHSPSLSNNLYGKRVNYALMPLMGGMYG---K

Na-NLP-10 (77) GPALGFSEGWAEKRAVMPFSGGVYGKRAP--ALPFSGGVYG---K

Hc-NLP-10.1 (82) --------GWQEKRAVMPFSGGLYGKRA---VMPFSGGLYG---K

Hc-NLP-10.2 (69) ----------GAKRAVMPYSGGMYGKRA---VMPFSGGLYG---K

Bx-NLP-10 (52) ----------LAKRAALPYSGGIYGKRA---ALPYSGGIYG---K

Gp-NLP-10 (71) IVE---GKRSPQGMAPSVLWNGAGDKRA---QLPFSGGIYG---K

181 225

Ce-NLP-10 (119) RGAMPFSGGMYGR--------------------------------

As-NLP-10 (72) RAVIPFSGGLYGKR--------TSQDFTKQNRL----------SL

Bm-NLP-10 (70) RAHSDIYDKSYQPLSLSAADEYNNKRLSLLSKIIHKENDMAQFNP

Di-NLP-10 (175) RTFVPFHGGMYGKRRSLPFNRYYNKQLLSPEITLNED-----LIP

Na-NLP-10 (117) RAAMPFSGGLYGKR--------SERYIRSP---------------

Hc-NLP-10.1 (113) RAAMPFSGGLYGKR--------ADRYIRSP---------------

Hc-NLP-10.2 (98) RSVMPFSGEAVHLI--------MVLNER-----------------

Bx-NLP-10 (81) RASVPFSGGIYGKRS------VDEAPVDHVNGI----------SI

Gp-NLP-10 (107) RSPLPFSGGIYGKRS------SSHQLYRSPLIFPTLD-----RQT

226 270

Ce-NLP-10 (132) ---------------------------------------------

As-NLP-10 (99) RSIPMSGGFFG----------------------------------

Bm-NLP-10 (115) LSHYHINEKVGHSPYQTLRQHYQLEDDNNDYYNQINDNSHLLQLL

Di-NLP-10 (215) SVYYDGNGKFGQFPYKLWRQYQLKEDNSDYYKDNSYPSQPFPELV

Na-NLP-10 (139) --MPISGGFFG----------------------------------

Hc-NLP-10.1 (135) --MPISGGIFG----------------------------------

Hc-NLP-10.2 (118) ---------------------------------------------

Bx-NLP-10 (110) RAMPINGGFYG----------------------------------

Gp-NLP-10 (141) RAMPFNGGMYGR---------------------------------

271 315

Ce-NLP-10 (132) ---------------------------------------------

As-NLP-10 (110) ---------------------------------------------

Bm-NLP-10 (160) LKLRSLLMNDEFNN-------------------------------

Di-NLP-10 (260) KPSNQFDYSFFKHNMQLLILSMFLLLAKFLFAENTLCIPPSIPAK

Na-NLP-10 (148) ---------------------------------------------

Hc-NLP-10.1 (144) ---------------------------------------------

Hc-NLP-10.2 (118) ---------------------------------------------

Bx-NLP-10 (121) ---------------------------------------------

Gp-NLP-10 (153) ---------------------------------------------

316 360

Ce-NLP-10 (132) ---------------------------------------------

As-NLP-10 (110) ---------------------------------------------

Bm-NLP-10 (174) ---------------------------------------------

Di-NLP-10 (305) DSQNVDCCKLPREIFPHKTIYEEFCSVLYFGVMADGPDGRFCRLS

Na-NLP-10 (148) ---------------------------------------------

Hc-NLP-10.1 (144) ---------------------------------------------

Hc-NLP-10.2 (118) ---------------------------------------------

Bx-NLP-10 (121) ---------------------------------------------

Gp-NLP-10 (153) ---------------------------------------------

361 405

Ce-NLP-10 (132) ---------------------------------------------

As-NLP-10 (110) ---------------------------------------------

Bm-NLP-10 (174) ---------------------------------------------

Di-NLP-10 (350) SSQKRQSINCDPGWIAFYEGLNQFCYKAFTINSTDKIVDIMSKKD

Na-NLP-10 (148) ---------------------------------------------

Hc-NLP-10.1 (144) ---------------------------------------------

Hc-NLP-10.2 (118) ---------------------------------------------

Bx-NLP-10 (121) ---------------------------------------------

Gp-NLP-10 (153) ---------------------------------------------

406 450

Ce-NLP-10 (132) ---------------------------------------------

As-NLP-10 (110) ---------------------------------------------

Bm-NLP-10 (174) ---------------------------------------------

Di-NLP-10 (395) GDPCRKKKETARLVRIPNQNINMFLYVRVLNGAQNLLCDSVDRNT

Na-NLP-10 (148) ---------------------------------------------

Hc-NLP-10.1 (144) ---------------------------------------------

Hc-NLP-10.2 (118) ---------------------------------------------

Bx-NLP-10 (121) ---------------------------------------------

Gp-NLP-10 (153) ---------------------------------------------

451 495

Ce-NLP-10 (132) ---------------------------------------------

As-NLP-10 (110) ---------------------------------------------

Bm-NLP-10 (174) ---------------------------------------------

Di-NLP-10 (440) YYYYIGLYRNDFDWRWRSYDEKAYSHLEEQGQQLLDYLNQPRSNH

Na-NLP-10 (148) ---------------------------------------------

Hc-NLP-10.1 (144) ---------------------------------------------

Hc-NLP-10.2 (118) ---------------------------------------------

Bx-NLP-10 (121) ---------------------------------------------

Gp-NLP-10 (153) ---------------------------------------------

496 523

Ce-NLP-10 (132) ----------------------------

As-NLP-10 (110) ----------------------------

Bm-NLP-10 (174) ----------------------------

Di-NLP-10 (485) TWPDGTRIFADGWHDVDDITTNVVMSNC

Na-NLP-10 (148) ----------------------------

Hc-NLP-10.1 (144) ----------------------------

Hc-NLP-10.2 (118) ----------------------------

Bx-NLP-10 (121) ----------------------------

Gp-NLP-10 (153) ----------------------------

NLP-11

1 55

Ce-NLP-11 (1) -------*MMSTLALVSLAIFGIAVVCA*APKPATVPVANEEDYLAALYGFEAPGSQ

As-NLP-11 (1) -----*MSKSILTLFTLLFIFAVAVIA*TREKRQLSPAVQLENELSPLIRLAGLHDG

Bm-NLP-11 (1) MYLIDLATVTAALAICFGDFVCAYGNHRQKKQLSPALQLEDNINSLLRLNELGY-

Di-NLP-11 (1) MYLIDLSTIT-ALAICFGDLMCGYASR-QKKQLSPALQLQDDINSLLRLNELSP-

Na-NLP-11 (1) ------*MMLAQIAVLTIAVCVAGA*NSVRVKRQLSPAVRLESELHPLMMGMYGFG-

Hc-NLP-11 (1) ---*MQVMALTQIVVLAFVVCMVGA*NSIRAKRQLSPAVRLETELHPLVMGMYGFGP

56 110

Ce-NLP-11 (49) FKGAPLQS----KRHISPSYDVEIDAGNMRNLLDIGKR-SAPMASDYGNQFQMYN

As-NLP-11 (51) ---GYYKRSFDYKRHLSPSLNVEDQLGRLIALQSVGKR--HSPAEQYGQQFELTL

Bm-NLP-11 (55) -KRS--------FGEPSHAMEFHDGLDDLFSLQNIGKRPALSPADQFARQMDAMQ

Di-NLP-11 (53) -KRS--------SGDQPYVLPVMN-LDNLLSLQNIGKRPALSPADQFIQQFDIMQ

Na-NLP-11 (49) -SEASKRSPLSLKRHISPSFDVEEDVGNLRTLMDIGKR-QVSLADDVGRQMQMYH

Hc-NLP-11 (53) ENNAYKRSALSLKRHISPSFDVEEDVGNMRTLMDIGKR-QLSVADDVGRQMQMYH

111 165

Ce-NLP-11 (99) RLIDAGKKKRSPAISPAY-----QFENAFGLSEALERAGRR--------------

As-NLP-11 (101) SISRGRLPFKVTASSNQSEFNKHYLKRIDNIVASTSRSGQKTCHLTIGRFTQSTI

Bm-NLP-11 (101) HLAAAGKK---RSLSPAT-----DFQQQLQLPHMLMDAGR---------------

Di-NLP-11 (98) HLMAAGKK---RSVSPAT-----DLEEQIKIPQVLIDVGR---------------

Na-NLP-11 (102) RLFDAGKKR--AVLSPSA-----DLQNAMDLTSYLERAGRK--------------

Hc-NLP-11 (107) RLFEAGKKR--AALSPSQ-----DLQSAVELSNYLERAGRK--------------

166 179

Ce-NLP-11 (135) --------------

As-NLP-11 (156) MVRVSRSSRSIVAF

Bm-NLP-11 (133) --------------

Di-NLP-11 (130) --------------

Na-NLP-11 (136) --------------

Hc-NLP-11 (141) --------------

NLP-12

1 45

Ce-NLP-12 (1) ---------------------------------------------

Ts-NLP-12 (1) ---------------------------------------------

Tm-NLP-12 (1) ---------------------------------------------

As-NLP-12 (1) ---------------------------------------------

Bm-NLP-12 (1) ---------------------------------------------

Di-NLP-12 (1) ---------------------------------------------

Na-NLP-12 (1) ---------------------------------------------

Hc-NLP-12 (1) ---------------------------------------------

Bx-NLP-12 (1) ---------------------------------------------

Gp-NLP-12 (1) MLLGICGFLSLSLMVVGFIAYSRRFYTYAYGRANAQMRFRRAVYT

46 90

Ce-NLP-12 (1) ---------------------------------------------

Ts-NLP-12 (1) -----------------------------------------*MVII*

Tm-NLP-12 (1) -----------------------------------------*MITS*

As-NLP-12 (1) ---------------------------------------------

Bm-NLP-12 (1) -------------------------------------------*ML*

Di-NLP-12 (1) ---------------------------------------------

Na-NLP-12 (1) ---------------------------------------------

Hc-NLP-12 (1) ---------------------------------------------

Bx-NLP-12 (1) --------------------------------------------*M*

Gp-NLP-12 (46) VRVRKSPRAPMGFQFGDFGEDTVVFNTLSCSVRHISTFNADDAII

91 135

Ce-NLP-12 (1) *MLRHHSCALLMLILVFVEVFA*TQSPT-------------------

Ts-NLP-12 (5*) RVSLMVFFAWLASFFLGQA*ALSQSQQ----VAKYNRAGFSPYLKR

Tm-NLP-12 (5) *RMMITAILVSFLMLLMGQTRS*ASQQQQQQPSFKLNRNGFVPFFKN

As-NLP-12 (1) -*MFGWIYLLLLLCIASSIHC*DLTPTR-------------------

Bm-NLP-12 (3) *RSWITSILFILGFISIAVNC*SLQSER-------------------

Di-NLP-12 (1) ---------------------------------------------

Na-NLP-12 (1) --*MIHCLLLLALVCTVLATADA*QSQT-------------------

Hc-NLP-12 (1) --*MIHYLLLLALACSVLATADA*QSPT-------------------

Bx-NLP-12 (2) *KLYILLMLALVGWVCAAA*ASEEEADR-------------------

Gp-NLP-12 (91) IIVFQHHAVLPNCFTTCHHCSNIAERHKQQAMFFDAVDVSSVGTV

136 180

Ce-NLP-12 (27) -----FDRQDRDYRPLQFGKRDGYRPLQFGKRD--YRPLQFGKRS

Ts-NLP-12 (46) FPALPSQYLKRSDMNNMLGIGVKRDDDQFDREDRDYRPLMFGKRY

Tm-NLP-12 (50) LPPLPRQYLKREILLPYYNAMAKREDDQFDREDRDYRPLMFGKRF

As-NLP-12 (26) -----FDRQDRDYRPLQFGKRDGYRPLQFGKKS--YRPLQFGKRR

Bm-NLP-12 (29) -----FDRQDRDYRPLQFGKRDGHRPLQFGKKS--FRPLQFGKRG

Di-NLP-12 (1) ---------------LQFGKRDGHRPLQFGKKS--FRPLQFGKRQ

Na-NLP-12 (25) -----FDRQDRDYRPLQFGKRDGYRPLQFGKRDS-YRPLQFGKRS

Hc-NLP-12 (25) -----FEREDRDYRPLQFGKRDGYRPLQFGKRD--YRPLQFGKRS

Bx-NLP-12 (28) -----FSRTDRDYRPLQFGKRGDFRPLQFGKKDN-YRPLQFGKRA

Gp-NLP-12 (136) SGE-QFERADRDYRPLQFGKRESYRPLQFGKRIVASRGLLFAPAV

181 214

Ce-NLP-12 (65) SGSSGPVVLEPIWEWQ------------------

Ts-NLP-12 (91) PDYQLPMFG-------------------------

Tm-NLP-12 (95) PDYQPPMFG-------------------------

As-NLP-12 (64) A-YDFSEYTPY-----------------------

Bm-NLP-12 (67) M-DEAPDYFYDI----------------------

Di-NLP-12 (29) S-ERFDRQDRDYRPLQVG----------------

Na-NLP-12 (64) P--LASAYLVPVL---------------------

Hc-NLP-12 (63) P--LASAFLVPAL---------------------

Bx-NLP-12 (67) AVVNHYLDLLPIDEIAN-----------------

Gp-NLP-12 (180) APPAFMHNLLHNSGLEEMDPTMDQYVLMVEERRK

NLP-13

1 50

Ce-NLP-13 (1) --*MQRSLQIFCIMSAIAMAYS*QGSRDDNQSAKRNDFSRDIMSFGKRSGN-

As-NLP-13 (1) --MKAIPIQFLLLSLFMFVNSQIFYDVG---TKREFDRDFMHFGKRS---

Bm-NLP-13.1 (1) -----*MTILILISLAAAIVQS*EYYAN-----YNDRLARDFMHFGKRSPNI

Bm-NLP-13.2 (1) --*METMRILILISLAAAIVQS*EYYAN-----YNDPLTRDSMNFEKRAVPS

Bm-NLP-13.3 (1) --*METMRILILISLAAAIVQS*EYYAN-----YNDPLTRDSMNFEKRAVPS

Di-NLP-13.1 (1) --*MEIITVLIVVSAVVITVQS*EYYVN-----YDGQLARDFMHFGKRS---

Di-NLP-13. (1) --------------------------------------------------

Na-NLP-13 (1) --*MTPPWSLVCLAVTITAVSS*QYILATGDNEKRNDFSRDIMHFGKRS---

Hc-NLP-13 (1) -*MMMPPWSLVCLAVTVTVAST*QYLSDGGENEKRNDFSRDIMHFGKRA---

Bx-NLP-13 (1) *MSTQTHFLAVVAATLCCLILCDA*EDAFYSIGDKRNFDREFMHFGKRATS-

51 100

Ce-NLP-13 (48) TADLYDRRIMAFGKRQ--PSYDRDIMSFGKRSAPSDFSRDIMSFGK----

As-NLP-13 (43) --NAFDRNFMNFGKRE--SQFSRDFLNFGKRESNVRSLKQYYDDLR----

Bm-NLP-13.1 (41) IIPQFDRNIASLKR----GEFSRDFMNFGKRSIG--DDWSDITLKVGDNP

Bm-NLP-13.2 (44) MRNMINLNKRSDSDN---GAFDREFLHFGKREVP--SKHNMINLNK--RS

Bm-NLP-13.3 (44) MRNMINLNKRSDSDN---GAFDREFLHFGKREVP--SKHNMINLNK--RS

Di-NLP-13.1 (41) -----PDMIQQFDGDN--GSPKRGE-------------------------

Di-NLP-13.2 (1) ----NFCLFISIFF----FKFSRDFLNFGKRSVD--DNWSDTMIRIDDTP

Na-NLP-13 (46) ---VLGLSALGNSN----PDFDRDMMSFGKRTG---LEREFMSFGK----

Hc-NLP-13 (47) ---YGNGRLVAYGG----PAFERDMMAFGKRSGG--FEREMMSFGK----

Bx-NLP-13 (50) --EDFGRSFMPFGKRATNDDFGRAFMPFGKRAANEEFGRAFMPFGKR---

101 150

Ce-NLP-13 (92) --RSSS--MYDRDIMSFGKRS---------PVDYDRPIMAFGKR----AE

As-NLP-13 (85) ----QK-KDFDRDFMHFGKR----------SDAFSRNFMNFGKR----DD

Bm-NLP-13.1 (85) ELLHKKSSTFDRDFMNFGKR----------AVPFERNLINSNKKSGIDSG

Bm-NLP-13.2 (87) --DSDNG-AFDREFLHFGKR----------EVPSKHNMINLNKRSDSNSG

Bm-NLP-13.3 (87) --DSDKG-AFDREFLHFGKR----------EVPSKHNMINLNKRSDSDNG

Di-NLP-13.1 (59) --------------------------------------------------

Di-NLP-13.2 (41) ESVHKKS-NFDREFMNFGKR----------MIPYDRNLINFNKKNSVKAD

Na-NLP-13 (82) --RS----PYEREFLPFGKRD---------LSDFNREMMSFGKR-----E

Hc-NLP-13 (84) --RS----PFEREFLSFGKR----------GSEFDREMLSFGKR-----D

Bx-NLP-13 (95) --ADAYDESFNRAFMPFGKRSPMTLDQLIAKKNMNRDFMHFGKR--VAPS

151 200

Ce-NLP-13 (125) DYERQIMAFGRRK-------------------------------------

As-NLP-13 (116) AFSRDFLSFGKRR-------------------------------------

Bm-NLP-13.1 (125) AFDREFLSFGKR--------------------------------------

Bm-NLP-13.2 (124) AFDREFLHFGKR--------------------------------------

Bm-NLP-13.3 (124) AFDREFLHFGKREVPSKHNMINLNKRSDSDKGAFDREFLHFGKREVPSKH

Di-NLP-13.1 (59) --------------------------------------------------

Di-NLP-13.2 (80) AFKREFLSFG----------------------------------------

Na-NLP-13 (112) DFDRNIMPFGRRRR------------------------------------

Hc-NLP-13 (113) EFERSMMAFGRRRK------------------------------------

Bx-NLP-13 (141) SFDREFMHFGKRR-------------------------------------

201 226

Ce-NLP-13 (138) --------------------------

As-NLP-13 (129) --------------------------

Bm-NLP-13.1 (137) --------------------------

Bm-NLP-13.2 (136) --------------------------

Bm-NLP-13.3 (174) NMINLNKRSDSNSGAFDREFLHFGKR

Di-NLP-13.1 (59) --------------------------

Di-NLP-13.2 (90) --------------------------

Na-NLP-13 (126) --------------------------

Hc-NLP-13 (127) --------------------------

Bx-NLP-13 (154) --------------------------

NLP-14

1 45

Ce-NLP-14 (1) ---------------------------------------------

As-NLP-14 (1) ---------------------------------------------

Bm-NLP-14 (1) -----*MLSLILLVLALGEFNDAKS*KFDEIKKMRDTIKGSRFRAKQ

Di-NLP-14 (1) *MTAYQMLSLVLLLLELDKINC*AKSEFNGMAKTQDPVKDS--RSNE

Na-NLP-14 (1) ---------------------------------------------

Hc-NLP-14 (1) ---------------------------------------------

Bx-NLP-14 (1) ----------------------------------*MTVHSRICLSA*

Gp-NLP-14 (1) ---------------------------------------------

46 90

Ce-NLP-14 (1) -------------------------------*MLHLIVLLVALSSA*

As-NLP-14 (1) -----MRNTFISLETRHEKYILRFSDSRLPRFILQFISATSISRS

Bm-NLP-14 (41) SLNSIDGSEFDGLGGIRIRIEKRSLDALQGEGFGMKKRALDALEG

Di-NLP-14 (44) KMRQIEEFRR-G---------KRALDALDDDGLGMDLRNMKASDD

Na-NLP-14 (1) ------------------------*MLFSLARCVLLVGCAQFICG*L

Hc-NLP-14 (1) ------------------------*MLFFLTRCVLVVGLSQLVCS*E

Bx-NLP-14 (12) *ALFVMFCSA*FVSSAVVVRLPVAPTRSVFFTPALFTKRALDALEGS

Gp-NLP-14 (1) --------------MAEAKNICRTKTPFSDFKLMVLLGIVLCSLF

91 135

Ce-NLP-14 (15) *VTA*GRPRRALDGLDGSGFG-FDKRALNSLDGAGFGFEKRALNSLD

As-NLP-14 (41) KDAKK--PSLDILEGAGFSPLKKRALDTLEGSGFGFSKRALDDLE

Bm-NLP-14 (86) EGFGMKKRALDALEGEGFG-MKKRALDALEGEGFGMKKRALDALE

Di-NLP-14 (79) AESRREKRSVDSVDGDGYG-MRLRYLGALEDDGFGLKKRALDELE

Na-NLP-14 (22) PEATHSPPALDDLDGNGFGGLKRVLSRRK---------RALDSLE

Hc-NLP-14 (22) QDATHPPPALDDLEGNGFGGMKKFSSRRK---------RALDSLE

Bx-NLP-14 (57) DFGLKRKRALDSLEGSDFG-LRKRALDSLEGSDFGLRKRALDSLE

Gp-NLP-14 (32) TPSTSIKESSRSSSLGRTGAIKVLALRPR---------RALDILE

136 180

Ce-NLP-14 (59) GQ--GFGFEKRALDGLDGAGFG-FDKRALNSLDGA-GFGFEKRAL

As-NLP-14 (84) GVGFGGMLRKRALDSLEGTDFG-LKKRALDYLEGS-GFGLMKRAF

Bm-NLP-14 (130) GE--GFGMKKRALDALEGEGFG-MKKRALDALEGE-GFGMKKRAL

Di-NLP-14 (123) GE--GFGLKKRALDALEGEGFG-FNKRALDALEGE-GFGFNKRAL

Na-NLP-14 (58) GGGFGFDKKKRALDELDGSGFG-FDKRALNALEGT-GFGFDKRAL

Hc-NLP-14 (58) GDGFGGLFKR-SLDSLEGDGFG-FDKRALNALDGT-GFGFDKRAL

Bx-NLP-14 (101) GA--DFGLKKRALDSLEGADFG-LKKRALDSLEGA-DFGLRKRAL

Gp-NLP-14 (68) SDD-FGGFRKRALDVMDGDGFGSFEKRALDTLEGDDFMGLKRKRL

181 225

Ce-NLP-14 (100) DGLD----GSGFGFDKRALNSLDGAGFGFEKRALNSLDGAGFGFE

As-NLP-14 (127) DAIEDDLEGAGFGLKKRALDAMEGAGFGFDKRALDSMEGTGFGFH

Bm-NLP-14 (171) DALE----GEGFGMKKRALDALEGEGFGMKKRALDALEGEGFGMK

Di-NLP-14 (164) DALE----GAGFGFKKRALDELEGAGFGLKKRALDSLEGTGFGIE

Na-NLP-14 (101) NSLE----GEGFGFDKRALNSLDGSGFGFDKRALNSLEGEGFGFD

Hc-NLP-14 (100) NSLE----GTGFGFDKRSLNSIEGTGFGFDKRSLNSIEGTGFGFD

Bx-NLP-14 (142) DSLE----GADFGLRKRALDSLEGSDFGLKKRALDALEGTGFGFD

Gp-NLP-14 (112) NELEG---DGFMGLDKRALDILDGDDFTGFSKRSEVNGG--LRRE

226 270

Ce-NLP-14 (141) KRALDGLDGAGFGFDKR--ALNSLDGAGFGFEKR---ALDGLDGA

As-NLP-14 (172) KRALDGLEGDGFGFTKK--ALDSLEGTDFDFGKRSYGPLKHLVFD

Bm-NLP-14 (212) KRALDALEGEGFGMKKR--ALDALEGEGFGMKKR---ALDALEGE

Di-NLP-14 (205) KKALDALEGAGFGLRKR--ALDSLEGAGFGIEKK---ALDALEGA

Na-NLP-14 (142) KRALNSLEGEGFGFDKR--ALNSLEGEGFGFDK---RALNSIEGD

Hc-NLP-14 (141) KRSLNSIEGTGFGFDRRRRSLDSTEGTGFGYRR---GKRTLPQIT

Bx-NLP-14 (183) KRALDMLEGSDFGLKKR--ALDSLEGADFGLKKR---ALDSLEGS

Gp-NLP-14 (152) LALLGKVQRQRQMRARR--ALDALEGNSFGFRR------------

271 315

Ce-NLP-14 (181) GFGFDKRALNSLDGNGFGFDKRTFKHS-----SN--KLRSVFRNL

As-NLP-14 (215) RNRPSLRYSLAQSNKNRKRLRSQLVRL--LEHHNYWPVFVVLLRR

Bm-NLP-14 (252) GFGMKKRALDALEGEGFGIDKRVLDALEGAGLRMNRPLKSSDSRT

Di-NLP-14 (245) GFGLKKRVIDVLKNAGYRRIRPKMNKGRFDGMRN-YPFQSSGLRT

Na-NLP-14 (182) GFGFDKRYYSHIINRNTSTLQNLKKQG--FDGRTAETRLTL----

Hc-NLP-14 (183) GTHPYLRLYKGKQSRGYKGDRSNP------NGSNTYLHNN-----

Bx-NLP-14 (223) DFGLRKRALDSLEGSDFGLKKRSPIARFYATGDDIRKLNDLKTQL

Gp-NLP-14 (183) ---------------------------------------------

316 355

Ce-NLP-14 (219) KGFKQH----------------------------------

As-NLP-14 (258) SLIKPYAMKRDQAASDWLRSLPHA----------------

Bm-NLP-14 (297) KGFHMNRFSTNGRHSHPHLTKF------------------

Di-NLP-14 (289) NRFRINRYFSNGRHSRPSKMIISSGNFAIRFISLSSIFLS

Na-NLP-14 (221) ----------------------------------------

Hc-NLP-14 (217) ----------------------------------------

Bx-NLP-14 (268) EVELRRRLEKEAEA--------------------------

Gp-NLP-14 (183) ----------------------------------------

NLP-15

1 45

Ce-NLP-15 (1) *MPSSSSSSSFFAAVLLVIVMMSTVES*AAVRLRPVGSLFFLNRPHE

Ts-NLP-15 (1) -----------------------MYRALGGKKNFNQFDVGLSGFS

Tm-NLP-15 (1) ------MLTVHQIKAQSDWRFDNVVGSEQRPLTAMNNLYKALAQK

As-NLP-15 (1) ---MNRLFSFMAKKTICSTFWQMNTEVTFLKNGAPMVVRIAGNAE

Na-NLP-15 (1) ----------------*MNSTAAPAMRVVLLIFTTMVVVSS*APSRQ

Hc-NLP-15.1 (1) -------------------------------------ILSAPSRQ

Hc-NLP-15.2 (1) ----------------MNSSAASALRIALFVCATVAIISSAPSRQ

Bx-NLP-15.1 (1) ------------------------------------------MDR

Bx-NLP-15.2 (1) ------------------------------*MNTQFLRFSVILIVL*

Gp-NLP-15 (1) ----------------KRSFDSLTGPGFTGLDTRAAFDTDFTNYD

46 90

Ce-NLP-15 (46) KRAFDSLAGSGFDNGFNKRAFDSLAGSGFGAFNK-----------

Ts-NLP-15 (23) KRPFDRLEGAHFSG-FDKRTLDMLEGSSMSGFNKRYFDVPRLYIK

Tm-NLP-15 (40) KYSFDRLDASGMSG-FNKRPFDRFESAQIGGLNK-----------

As-NLP-15 (43) KRAFDSLTGAGFTG-FDKRAFDSLTGSGFTGFDK-----------

Na-NLP-15 (30) KRAFDSFAAPGFDA-FNKRAFDTLAGQGFEAFNK-----------

Hc-NLP-15.1 (9) KRAFDTFAGS-FSG-FDKRAFDSLAGSGFSAFDK-----------

Hc-NLP-15.2 (30) KRAFDTFAGS-FSG-FDKRAFDSLVSFFFLLN-------------

Bx-NLP-15.1 (4) KRSFDSFTGAGFTG-MDKRSFDALTGSGFTGMDK-----------

Bx-NLP-15.2 (16) *GCVRA*ALAGVANTG--EKRAFDALTARGFNGFDK-----------

Gp-NLP-15 (30) KRSFDSFTGPGFTG-LDKR-FEPFDGYGFNGFEK-----------

91 135

Ce-NLP-15 (80) RAFDSLAGSGFGAFNKRA--FDSLAGSGFSGFDKRAFDSLAGQGF

Ts-NLP-15 (67) RNFDKFDSATLSGFRRKR-MLDNIDSAGFAFSNKTLLYQQLHFTF

Tm-NLP-15 (73) KAFDMLENSKLGGFQKRP--FDSIETGDFKEGRAPV---------

As-NLP-15 (76) RSLDSPANRGFTHFDKRA--FDSLTGAGFTGFDKRSFDSLNSRGF

Na-NLP-15 (63) RAFDSLSGSGLTPFNKRA--FDSLAGNGFGGFDKRSPLVRRSYAI

Hc-NLP-15.1 (41) RAFDSLSGSGLTPFNKRA--FDSLAGSGFTGFDKRSPIALNAGYY

Hc-NLP-15.2 (60) -AFGNLLKPMIFPLLLG----------------------------

Bx-NLP-15.1 (37) RSFDSFTGAGFTGMDRKR---------ALARMMAQ----------

Bx-NLP-15.2 (48) RAFDSF---------------------------------------

Gp-NLP-15 (62) RSFDSFMGPGFTGMDKKKRAFDSFTGPGFTGMDKK----------

136 180

Ce-NLP-15 (123) TGFEKRAFDTVSTSGFDDFKL------------------------

Ts-NLP-15 (111) RSLSWYEIINCYINLSSIILMRSIALA------------------

Tm-NLP-15 (107) ---------------------------------------------

As-NLP-15 (119) TGFDKRAFDSLVGHGFTGFD-------------------------

Na-NLP-15 (106) VVLHIDLPSFIIRIIALVMLYLPDIKNYPERETNSVDQHKRISDV

Hc-NLP-15.1 (84) QPLREDALLL-----------------------------------

Hc-NLP-15.2 (76) ---------------------------------------------

Bx-NLP-15.1 (63) ----KRAFDSMTGRGFTGFD-------------------------

Bx-NLP-15.2 (54) ---------------------------------------------

Gp-NLP-15 (97) ----KRAFDLFTGPGFTGMDRK-----------------------

181 225

Ce-NLP-15 (144) ---------------------------------------------

Ts-NLP-15 (138) ---------------------------------------------

Tm-NLP-15 (107) ---------------------------------------------

As-NLP-15 (139) ---------------------------------------------

Na-NLP-15 (151) YALIPVTYKRWLLSLLYTNSKSTGITVGAECLSKETKHKTRRRHA

Hc-NLP-15.1 (94) ---------------------------------------------

Hc-NLP-15.2 (76) ---------------------------------------------

Bx-NLP-15.1 (79) ---------------------------------------------

Bx-NLP-15.2 (54) ---------------------------------------------

Gp-NLP-15 (115) ---------------------------------------------

226 236

Ce-NLP-15 (144) -----------

Ts-NLP-15 (138) -----------

Tm-NLP-15 (107) -----------

As-NLP-15 (139) -----------

Na-NLP-15 (196) ADTQQTHRLGC

Hc-NLP-15.1 (94) -----------

Hc-NLP-15.2 (76) -----------

Bx-NLP-15.1 (79) -----------

Bx-NLP-15.2 (54) -----------

Gp-NLP-15 (115) -----------

NLP-17

1 50

Ce-NLP-17 (1) --------------------------------------------*MFSKSI*

As-NLP-17 (1) ------------------------------------------------*MQ*

Na-NLP-17 (1) ---------------------------------------------*MRQSV*

Hc-NLP-17 (1) ---------------------------------------------*MRQCF*

Bx-NLP-17 (1) MNLCFHCPPFHPFNLFFVVSRKSRVVAGRVIFAGQTYCNRERLTEHSFQL

Gp-NLP-17 (1) -----------------------------------------*MLLNLLAVY*

51 100

Ce-NLP-17 (7) *LFCLLVLVFNVFG*ANFEN--DQDVMRPPFQALKRGSLSNMMRIGKRQ--M

As-NLP-17 (3) *TVVVIITLALLTLPSSRC*IPRYGRMIGTEQWTSNTSQDTVEQSSSDSDVM

Na-NLP-17 (6) *VPILLVWFICLASA*QFVDVDRPEILRPDVRFLKRNNLSNMMRLGKRG--L

Hc-NLP-17 (6) *VLLLLWIVSA*VCMQALDEGTREELMSPDVRFLKRNSLSIMMRLGKRVDAL

Bx-NLP-17 (51) GFSFSTTYNRDVSNLHNHKEMAVLVNSYVWQLIVLALVCLACTFATAQPV

Gp-NLP-17 (10) *AVALMTTLYMPVQS*AYTYQKMDELMPELRQYLGRTHLGTLKARKFNDDDN

101 150

Ce-NLP-17 (53) SRQQEYVQFPNEG------VVPCESCNLGTLMRIGRR-------------

As-NLP-17 (53) PLWWTHVR--------------ERKNILSNMMRIGRRSLSNP--------

Na-NLP-17 (54) RRQM-----------------LCEDCNLGNLMRLGRR-------------

Hc-NLP-17 (56) KEQE-----------------PCVDCSLGNLMRLGRR-------------

Bx-NLP-17 (101) GILGLETQGFNESPFEQIQLRERKSNNLRNLMRIGRRTP--------ADQ

Gp-NLP-17 (60) AFQQIR---------------ERKSNNLRNLMRIGKRNVPIADNFAREDE

151 179

Ce-NLP-17 (84) -----------------------------

As-NLP-17 (81) -----------------------------

Na-NLP-17 (74) -----------------------------

Hc-NLP-17 (76) -----------------------------

Bx-NLP-17 (143) PIVPG-FEMRQAPLDYAQFFRPNAAIF--

Gp-NLP-17 (95) GGVGALSPVFYIPTDFLQAYRPQSVWLRR

NLP-18

1 60

Ce-NLP-18 (1) -----------*MNANVYSIVYFLSFLVLCISAQLHA*DSGATEVDG-----------IVDK

As-NLP-18 (1) ------------*MRTFLALVLVGSLFAGVFC*QELAEDFSPDKRGMSSFAKR--ARFNFAK

Bm-NLP-18 (1) -----------*MASGFKVFVIFMSLLLTIIPCQE*ITDESLDRHDSSVFGKR--AKFAFAK

Di-NLP-18 (1) ----------------------------------MTDEVLNKHESSAFGKR--AKFAFAK

Na-NLP-18 (1) MCMVNKFQWVYIMQSMFTLAFVCTLTTMLLAQQVVDDVTAEKRVRS---------FAFAK

Hc-NLP-18 (1) ------------*MQSTFTVVLVCTLASMILA*QQMIDDVPAEKRMRS---------FAFAK

Bx-NLP-18 (1) ------------*MHSTLGLIVCASLAVMVLS*QELSEQDSMDKRARSFAFAKRARSFAFAK

61 120

Ce-NLP-18 (39) RSP----YRAFAFAKRS--------DEENLDFLEKRARYGFAKRSPYRTFAFAKRASPYG

As-NLP-18 (47) RYP----SR-FAFAKRF--------DYDDWAEADKRAMRFAFAKRSMRNFAFAKRSP---

Bm-NLP-18 (48) RHP----SR-FAFAKRY--------DDEDVNYYDKKAMHFESPIHPTRNFAFAKRGP---

Di-NLP-18 (25) RYP----SR-FAFAKRY--------DDEDMYYYDKKAMHFEYPMHYMRNFAFAKRDP---

Na-NLP-18 (52) RSP----YRQFAFAKRS--------EDDDEEEVEKRARFAFAKRSPYRQFAFAKRASPYG

Hc-NLP-18 (40) RSP----YRQFAFAKRSDE------VVEDDGELEKRARFAFAKRSPYRQFAFAKRGSPYG

Bx-NLP-18 (49) KFDPYPFGRSFAFAKRSAEEPLLDVDSLEEDPMDKRARFAFAKRP--RNFAFAKRAR---

121 133

Ce-NLP-18 (87) FAFAKRGQFSSFA

As-NLP-18 (91) --------YSSFA

Bm-NLP-18 (92) --------YLSFA

Di-NLP-18 (69) --------YLLF-

Na-NLP-18 (100) FAFAKRG-YSSFA

Hc-NLP-18 (90) FAFAKRG-YSSFA

Bx-NLP-18 (104) ---------FAFA

NLP-19

1 52

Ce-NLP-19 (1) *MLLRGVCLALLILVTIVQC*QNDNDLKEEKRRIGLRLPNFLRFKDPDALMIHK

As-NLP-19 (1) ---------------------KRRIGLRLPNILHMKVSIVVFQSYRHSSSQK

Na-NLP-19 (1) -----------------------*MPRFWYILFLLFIGSLP--IFS*QTEDVYK

Hc-NLP-19.1 (1) ----------------------*MISRIYCLLCLIFILSAVPNATS*QEVELYK

Hc-NLP-19.2 (1) -*MSSISPLHLGPLLILISAHFSMISRIYCLLCLIFILSAVPNATS*QEVELYK

Bx-NLP-19 (1) --------------------------------------MRPLNVESGERLQK

Gp-NLP-19 (1) ----------------------------------SRLLNSPFRFCLSHIPDS

53 104

Ce-NLP-19 (53) RRIGLRLPNMLKFKDSSNMYHLE-----------KRRMGMRLPNIIFLRNE-

As-NLP-19 (32) RRFGLRLPNIIYIRRQRNLSFNYTI-------LQMACFGLRLPNIIYIRRQR

Na-NLP-19 (28) RRIGMRLPNMLHYKPTAME---------------KRRIGMRLPNIIYLRSEP

Hc-NLP-19.1 (31) RRIGMRLPNMVHLKPLVR----------------KRRIGMRLPNIIYMRSEP

Hc-NLP-19.2 (52) RRIGMRLPNMVHLKPLVR----------------KRRIGMRLPNIIYMRSEP

Bx-NLP-19 (15) RRLVMGMPNILHMGSKRMVKRMIERNAQRELVMNKRRISMHMPNLVYMRPQK

Gp-NLP-19 (19) RKIGIRVPGFPIRQGYCS-----------------RKIGIRVPGFPIRQGYC

105 156

Ce-NLP-19 (93) -KKNVLEY--------------------------------------------

As-NLP-19 (77) ----------------------------------------------------

Na-NLP-19 (65) EKKDNWEYDSF-----------------------------------------

Hc-NLP-19.1 (67) AKKNIWEYE-------------------------------------------

Hc-NLP-19.2 (88) AKKNIWEHQALLDRHDKNIISDTSTRDRRELLALFTPFWMTSRLRANPITIQ

Bx-NLP-19 (67) ----------------------------------------------------

Gp-NLP-19 (54) SRKIGIRVPGSDSAGIW-----------------------------------

157 189

Ce-NLP-19 (100) ---------------------------------

As-NLP-19 (77) ---------------------------------

Na-NLP-19 (76) ---------------------------------

Hc-NLP-19.1 (76) ---------------------------------

Hc-NLP-19.2 (140) TLFNNLYLNKFEWQHKNGKTSVLRKPWLGMTSQ

Bx-NLP-19 (67) ---------------------------------

Gp-NLP-19 (71) ---------------------------------

NLP-20

1 40

Ce-NLP-20 (1) ----------------------*MQVTLLALLLLIVPFFAF*

As-NLP-20 (1) ---------------------------------MSSTWSW

Na-NLP-20 (1) ----------------------*KRMMCTLAVLILASIIAV*

Hc-NLP-20 (1) *MRTLKTRTARLALAQNTAKISSNKVMEAVPLFVILSTIVL*

Bx-NLP-20 (1) -----------------------------*MSPLLTTVTAL*

41 80

Ce-NLP-20 (19) *A*ASQYSDDDSELMSNNERFARDLELRKKFAFAFAKR----

As-NLP-20 (8) LCSFAFTPKKAGRFARHASGQQNE----LSFAFAKRAP--

Na-NLP-20 (19) *SSG*FAYDDEHNTDLARERFARELDQKQKFAFAFAKRGEDV

Hc-NLP-20 (41) *STGFA*YDDQENTDLTRDGFARDFA-KQRFAFAFANRYPEI

Bx-NLP-20 (12) *FLFSSVYG*QRQNAITDKVGQGNFAFARPFAFALTENGE--

81 120

Ce-NLP-20 (55) --------SAGD-------------ADVVIEAR---SGPQ

As-NLP-20 (42) --------FAKR------------EMQFAFEEPS-VDAER

Na-NLP-20 (59) PDAKQRFARDLENAKKFAFAFAKRSSDMVEESRSARFARD

Hc-NLP-20 (80) PEERSRFARDLDNSKKFSFAKK--SPDFSEESR--GFARE

Bx-NLP-20 (50) -----WYPIAKRSR----------TSTFAFKETPKEKERD

121 160

Ce-NLP-20 (71) AHEGAG------MRFAFAKRRAPKEFARFARASFA-----

As-NLP-20 (61) FARFAR------AAFAFAKRSPFAFA--------------

Na-NLP-20 (99) LDNDKK------FAFAFAKRLADDELRARYPRTPMEDQGF

Hc-NLP-20 (116) LEGNKK------FAFAFAKRFAGSHNEVRRQDNGRSNFAA

Bx-NLP-20 (75) SFAFAKRSEDPKFAYDFAKRAKFAFLKK------------

161 181

Ce-NLP-20 (100) ---------------------

As-NLP-20 (81) ---------------------

Na-NLP-20 (133) AREFAKRSDDRLMRFARPSFA

Hc-NLP-20 (150) PNFTNVHSVSEGV--------

Bx-NLP-20 (103) ---------------------

NLP-21

1 40

Ce-NLP-21 (1) ----*MRNSLFTTLFFGLAALVMVLN------A*QYTSELEE

As-NLP-21 (1) MIFIAKHQANSATFIGEKKCNQINTR-----WVNDCTFVS

Na-NLP-21 (1) --------MLPPAAVSHADDDSIEK------RGGGRAFVP

Hc-NLP-21.1 (1) *MRAVALVVIAAVAAISNA*EEYSIEK------RGGGRAFVP

Hc-NLP-21.2 (1) -----------XAAISNAEEYSIEK------RGGGRAFVP

Bx-NLP-21 (1) MDVTQKPMPNGERLVGTRPRGGVKKGREKVSELGFSIVAT

Gp-NLP-21 (1) --*RIVYIIPISSALFILPRIGVA*TS------ESDVDALAT

41 80

Ce-NLP-21 (31) DEKRGGARAMLHK------RGGARAFSADVG---------

As-NLP-21 (36) SNPLGHLKNFEKCS-------SIRLVVLLSEKQIYCMIAI

Na-NLP-21 (27) IEERGGARAFFEDN----KRGGARVFPADKKGGAHA----

Hc-NLP-21.1 (35) IEERGGARAFFGDE----KRGGARAFVVDTRGGARA----

Hc-NLP-21.2 (24) IEERGGARAFFGDE----KRGGARAFVVDTRGGARA----

Bx-NLP-21 (41) DRSWPGVVCKIAS----IMNLGVRTIDLFLWVSLFGILTQ

Gp-NLP-21 (33) EQKRGGARAFNFFAPPDEKRGGARAFNFFAPDEKRGGARA

81 120

Ce-NLP-21 (56) -----------------DDYKRGGARAFYDEKRGG-----

As-NLP-21 (69) G----------------KRQLRIAQEPDVLEKRGG-----

Na-NLP-21 (59) ----------------FRELKRGGARAFVPLEVRGGAVPS

Hc-NLP-21.1 (67) ----------------FREVKRGGGRAFLPVEERG-----

Hc-NLP-21.2 (56) ----------------FRDLKRGGGRAFLPVEERG-----

Bx-NLP-21 (77) IVYGASFVQNLEDAELNEEDKRGGGRAFLNTDDKRG----

Gp-NLP-21 (73) FNF------------FAPDEKRGGTRAFNFFVSDALPSSY

121 160

Ce-NLP-21 (74) -------------ARAFLTEMKRGGARVFQGFEDE-----

As-NLP-21 (88) -------------GRPFTLVDKRAGGRFFKIEDVDALE--

Na-NLP-21 (83) Q-------LEKRGGGRAFVEPKRGGARAFYGIEQRGGARA

Hc-NLP-21.1 (86) ------------GARAFFKDDKRGGARAFVPVKEEDEGQS

Hc-NLP-21.2 (75) ------------GARAFFKDDKRGGARAFVPVKEEDEGQS

Bx-NLP-21 (113) ------------GGRAFQFNDKRGGARAFAPVDVK-----

Gp-NLP-21 (101) EKRSGIQTFRDDYDEKQAGELKRAGGRLFRMVDLPDGDDF

161 200

Ce-NLP-21 (96) -----KRGGARAFMMD-------KRGGGRAFGDMM-----

As-NLP-21 (113) -----KRGGGRPFYTQTDE----KRGGGRSFRLSSLGE--

Na-NLP-21 (116) FFNDEKRGGARPFTDW-------KRGGARGFSTIE-----

Hc-NLP-21.1 (114) FTDLEKKGGARAFFVP-------KRGGGRAFLPIE-----

Hc-NLP-21.2 (103) FTDLEKKGGARAFFVP-------KRGGGRAFLPIE-----

Bx-NLP-21 (136) ------RGGARAFAPID-----YKRGGGRAFSGFNGGN--

Gp-NLP-21 (141) VPEGKKRGGARPFYGGGYMDGTWKRAGGRYFMRHFDDSPF

201 240

Ce-NLP-21 (119) -----KRGGARAFVENSK----RDEDWVIR---PFEDDRL

As-NLP-21 (142) -----KRMAAQKIAMPELDFYEGALAEIGNWP--------

Na-NLP-21 (144) -----LRGGARAFFEDEK----RGGARAFK-RGGGRAFVL

Hc-NLP-21.1 (142) -----ERGGARAFFQEPK----RGGARAFPKRGGGRAFVP

Hc-NLP-21.2 (131) -----ERGGARAFFQEPK----RGGARAFPKRGGGRAFVP

Bx-NLP-21 (163) YYLLNKRGGGRSFYGGFN----YGSLYAPSFPLSYEKKAD

Gp-NLP-21 (181) AGWMAKRGGARAFFGDADGPFNSASYWAPRFVLPINKHPR

241 280

Ce-NLP-21 (147) E---------------------------------------

As-NLP-21 (169) --------------------------------------FI

Na-NLP-21 (174) D---------------------------------------

Hc-NLP-21.1 (173) Q---------------------------------------

Hc-NLP-21.2 (162) Q---------------------------------------

Bx-NLP-21 (199) YVP--------------------------------YYLTE

Gp-NLP-21 (221) RSLLMGPLENVEDEAEQQRMDSSPSPFFTNNHNFDGRANG

281 320

Ce-NLP-21 (148) -KRGGGRSFPVK-------------------P--------

As-NLP-21 (171) EKRAGARSFPVLEDLLE-----------------------

Na-NLP-21 (175) -KRGGARMFQNSKRDYTDDYPDWLMYADHEQSPFEVDENV

Hc-NLP-21.1 (174) -KRGGARIFQN----------------THKRP--------

Hc-NLP-21.2 (163) -KRGGARIFQN----------------THKRP--------

Bx-NLP-21 (207) MKRAGGRAFQPKYYDP-------------YFWYVD-----

Gp-NLP-21 (261) RKRGGARAFNGAEETLLNVANLAKRGGGRNFNEMEDTEEY

321 355

Ce-NLP-21 (160) -----GRLDD-------------------------

As-NLP-21 (188) ------KRRIYDAFSRFP-----------------

Na-NLP-21 (214) DVWQAGKRGGAHSFPVENSTKIRGNY---------

Hc-NLP-21.1 (189) -----WKRDDARSLPRTVESV--------------

Hc-NLP-21.2 (178) -----RKRDDARSLPRTVESI--------------

Bx-NLP-21 (229) ----QTKRAGGRSFPISDESKEKRMLEEQADSRTI

Gp-NLP-21 (301) K----KKRFGERIVSANPASCAMNFQPTQ------

NLP-35

1 48

Ce-NLP-35 (1) -*MPRVSSFIVFLTFMVALLAVSHA*AVVS-----GYDNIYQVLAPRFRR

As-NLP-35 (1) -MPRYASFVVLTAIILYVVESASSEQFG-------ES-YLPGFDELFG

Na-NLP-35 (1) --*LRATLVILVVAVVLALCHA*LPIEENNPPVDYGSLDNFFYLSPRLRR

Hc-NLP-35 (1*) MSLRAVLVILVVAVVMVLCHA*LPIDDTG--ADYGNLENFFYL-PRLRR

49 96

Ce-NLP-35 (43) ARMMSFDAEAPQYLQHLLQNLKPRFRRSV-------------------

As-NLP-35 (40) GRER--RDSKRAFLQQLMQRLKPRFRRDYAVNDHDITNFTRNRSELQL

Na-NLP-35 (47) DPLS--FGGQNAYLHQLLQNLKPKFRRSV-------------------

Hc-NLP-35 (46) DQLA--FSGQNAYLRQLLQNLKPKFRRSV-PLVPSIIANGLSLRDLSP

97 112

Ce-NLP-35 (72) ----------------

As-NLP-35 (86) S---------------

Na-NLP-35 (74) ----------------

Hc-NLP-35 (91) ALVDVTSMIFRAPVVY

NLP-36

1 50

Ce-NLP-36 (1) ---------------MSVDLKQQLELADYLGALAVWCIFFGVLFILSVIF

Ts-NLP-36.1 (1) ----------------MVNLKQDVNVADYFGFIVVLVTFFVIIFVISTTC

Ts-NLP-36.2 (1) ----------------MVSLKQELQVLDYFGAFCVFISFFVIILIISVTC

Tm-NLP-36.1 (1) ----------------MVNLKQEVSIGDYFGAAIVFCLFFLIISVISFTC

Tm-NLP-36.2 (1) ----------------MVYLGQELSVLDYFSPLIVFGTFLTIVFIISATC

As-NLP-36.1 (1) ------------------MPQQEFEFIDYLGPLAVSICFAV-ALFLLSLV

As-NLP-36.2 (1) ----------------MVDLKQQLDVLDYFGPVAVWISFFAIVFIISVTC

Bm-NLP-36.1 (1) -------------------MKQIFGFTDYLGPLAASMCFFV-AIFLLSLI

Bm-NLP-36.2 (1) ------------------MPKQEFEFIDYLGPLAVSVCFVI-ALFILSAI

Bm-NLP-36.3 (1) ----------------MVSLKQEFEFYDYFGPVAVISLFAITILLISFFI

Bm-NLP-36.4 (1) ----------------MVDLKQQLDMLDYLGPIAVWISFFSITFIISVTC

Di-NLP-36.1 (1) ----------------MVSLKQEFEFYDYFGPLAVISIFAITVLLISFFI

Di-NLP-36.2 (1) ----------------MVDLKQQLDMLDYLGPIAVWISFFSIIFIISVTC

Na-NLP-36.1 (1) ---------------MSIDLKQNLDVIDYFGALVVWVIFFSILFIVSVTC

Na-NLP-36.2 (1) ------------------MAKQEFEPIDYFGPVVVAAIFAITLLLISFFV

Hc-NLP-36.1 (1) ------------------MAKQDFETIDYFGPVVVAAIFAVALFLISFFI

Hc-NLP-36.2 (1) ------------------MGKQTFTLLDYCGPLIVGAVFAS-ILFILSLM

Hc-NLP-36.3 (1) ---------------MSVDIKQQLDVIDYFGALVVWVIFFSVLFIVSVTC

Hc-NLP-36.4 (1) ---------------MSIDLKQHLDVIDYFGALVVWIIFFSFVFIVSVTV

Hc-NLP-36.5 (1) ------------------MAKQDFETIDYFGPVVVAAIFAVALFLISFFI

Bx-NLP-36.1 (1) ------------------MPKQDFLFTDYMGPVVAAVIFAICLLVVSFLL

Bx-NLP-36.2 (1) ------------------MPKQDFELIDYLGPVVVAIIFAICLLLISFTV

Bx-NLP-36.3 (1) MAETLKEDVRMSSDLLKKMPKQSLELIDYLGPIAAAVIFFSTMFILSVTV

Gp-NLP-36.1 (1) --------------------MGPTGTQGIGITKVLCRQFVLLVFLISFLF

Gp-NLP-36.2 (1) --------------------MGPTGTQGIGITKVLCRQFVLLVFLISFLF

Gp-NLP-36.3 (1) ------------------MPKQSLDLIDYFGPLAAWIVFFSIIFVVSVTC

51 100

Ce-NLP-36 (36) N-FVCIKKDDDVTALERW--------------------------------

Ts-NLP-36.1 (35) ILWFCVDKTQEATVFDKWRG------------------------------

Ts-NLP-36.2 (35) ILWFCVSPTDDVTVFAKVRSLLTFL-------------------------

Tm-NLP-36.1 (35) ILWFCISKKDEATVVDKWRG------------------------------

Tm-NLP-36.2 (35) L-LCCCVEDVDSTVFSKFRR------------------------------

As-NLP-36.1 (32) INFIWITKKDDRTVFEKTERTHTE--------------------------

As-NLP-36.2 (35) ILWCCVSENDDSTVFAKWGM------------------------------

Bm-NLP-36.1 (31) INFIWISKDDERTIFEKFGSN-----------------------------

Bm-NLP-36.2 (32) INFIWITKNDDRTVFEKFGSTFDL--------------------------

Bm-NLP-36.3 (35) LNFFFISKHDEPTVFERIASKHNL--------------------------

Bm-NLP-36.4 (35) ILWCCVSETDDKTVFAKWGM------------------------------

Di-NLP-36.1 (35) LNFFFISKHDEPTVFERIASKHNL--------------------------

Di-NLP-36.2 (35) ILWCCVSETDDKTVFAKWGM------------------------------

Na-NLP-36.1 (36) INWCCIQKHDDITVLEEFSIRLPYGSCVSASAANSTILILEMKISLRKGS

Na-NLP-36.2 (33) INFFCITKYDDFTQFEKFGAKHN---------------------------

Hc-NLP-36.1 (33) INFFCITKYDDVTQFEKFGAKRN---------------------------

Hc-NLP-36.2 (32) MNFIFIRKRDEITSFEKLGAKYNL--------------------------

Hc-NLP-36.3 (36) INWCCIQKHDDITVLEEW--------------------------------

Hc-NLP-36.4 (36) INWCCIAKYDDVTVLEEW--------------------------------

Hc-NLP-36.5 (33) INFFCITKYDDVTQFEKFGAKRN---------------------------

Bx-NLP-36.1 (33) LNYCFVLKTDELTVFERFGSKHN---------------------------

Bx-NLP-36.2 (33) INWYCITHRDDLTVFEKLGRRADIR-------------------------

Bx-NLP-36.3 (51) ILWCFVTPNDDKTVFAKWGL------------------------------

Gp-NLP-36.1 (31) VNFFLITEEDDLTVFEKFGARHFG--------------------------

Gp-NLP-36.2 (31) VNFFLITEEDDLTVFEKFGARHFG--------------------------

Gp-NLP-36.3 (33) ILWCCVSETDDTTVFAKWGM------------------------------

101 150

Ce-NLP-36 (53) ------------------------GYKKNIDMKLGPHRRSMVARQIPQTV

Ts-NLP-36.1 (55) ------------------------RRKGSVASRKSKHSMLISPTNY----

Ts-NLP-36.2 (60) ----------------------VLVVIVEQQLKKNLHNSICNNDSLLNAF

Tm-NLP-36.1 (55) ------------------------RRKESQTYPKS-KPLLAHGSNY----

Tm-NLP-36.2 (54) -------------------------SSRRRSYTMSSERTSSR--------

As-NLP-36.1 (56) -----------------------AAWGLRHPDAGFLLDSSENFRVIGFI-

As-NLP-36.2 (55) ------------------------GPRPRVSSQQQKYASGSPKAE-----

Bm-NLP-36.1 (52) -----------------------LCGVHRMRHRSKKGTKMMNKLMVNSYG

Bm-NLP-36.2 (56) -----------------------RCGVHRMRHRPNKSWKRVQLIDNQDV-

Bm-NLP-36.3 (59) -----------------------RLGPHSVKTVISRKQRYMKDNNSGDPD

Bm-NLP-36.4 (55) ------------------------GPRHQLTVAQQKYDSTSTHKAL----

Di-NLP-36.1 (59) -----------------------RLGPHSVKTVISRKQRYMKDSNSGDPD

Di-NLP-36.2 (55) ------------------------GPRHQLTVAQQKYDSTSTHKAL----

Na-NLP-36.1 (86) RADRKRVTTFTESIANVFDWFFEWGYNHKLHMRMGPHRPSVVARNVRHPL

Na-NLP-36.2 (56) ----------------------WRMGPHRLHIVKKGGFVADAYDDDDEEA

Hc-NLP-36.1 (56) ----------------------WRLGPHRMLHAKGGGLILKPEDDDDDTY

Hc-NLP-36.2 (56) -----------------------RVGPHRVSVVKRYVERPPLLEE-----

Hc-NLP-36.3 (54) ------------------------GYNHHVNMRLGPHRPSVVARNVRHSK

Hc-NLP-36.4 (54) ------------------------GYNHNLRMRMGPHRPSMVGRKLGHRT

Hc-NLP-36.5 (56) ----------------------WRLGPHRMLHAKGGGLILKPEDDDDDTY

Bx-NLP-36.1 (56) ----------------------VRLGPHSLDSIKRRSQRRLTEDEGCPIV

Bx-NLP-36.2 (58) ------------------------LGPHKMSVIRRGGYASTYAKDEEALM

Bx-NLP-36.3 (71) ------------------------GPQPK--NQQVVYRDGVASESLLVV-

Gp-NLP-36.1 (55) ----------------------VRLGPHSLAQIKSGGPSSVAAGQNVAPQ

Gp-NLP-36.2 (55) ----------------------VRLGPHSLAQIKSGGPSSVAAGQNVAPQ

Gp-NLP-36.3 (53) ------------------------GPQRRKRSGHHTLLNTPPQQQPKAE-

151 176

Ce-NLP-36 (79) VADH----------------------

Ts-NLP-36.1 (77) --------------------------

Ts-NLP-36.2 (88) LIVSSTPLFILLYYSVI---------

Tm-NLP-36.1 (76) --------------------------

Tm-NLP-36.2 (71) --------------------------

As-NLP-36.1 (82) --------------------------

As-NLP-36.2 (76) --------------------------

Bm-NLP-36.1 (79) PAVDTTVV------------------

Bm-NLP-36.2 (82) --------------------------

Bm-NLP-36.3 (86) GSVHESRKVVFTKEPEPEVHINSA--

Bm-NLP-36.4 (77) --------------------------

Di-NLP-36.1 (86) GSIQGSRKVVFTKEPEPEVHINSA--

Di-NLP-36.2 (77) --------------------------

Na-NLP-36.1 (136) KEDNAL--------------------

Na-NLP-36.2 (84) AYFA----------------------

Hc-NLP-36.1 (84) YA------------------------

Hc-NLP-36.2 (78) --------------------------

Hc-NLP-36.3 (80) VESDSL--------------------

Hc-NLP-36.4 (80) STTIPGHTGHP---------------

Hc-NLP-36.5 (84) YA------------------------

Bx-NLP-36.1 (84) TENTKVPIPQVQVDAASTS-------

Bx-NLP-36.2 (84) KKQSHAAQVALASEIA----------

Bx-NLP-36.3 (94) --------------------------

Gp-NLP-36.1 (83) KEQRKAQKDEKKAEEFEMKEHRTNVF

Gp-NLP-36.2 (83) KEQRKAQKDEKKAEEFEMKEHRTNVF

Gp-NLP-36.3 (78) --------------------------

NLP-37

1 50

Ce-NLP-37 (1) ---------------------------*MSSRISVSLLLLAVVATMFFTAN*

As-NLP-37 (1) ---------------------------MKFTALPALLAFAIIAVNANSEE

Bm-NLP-37 (1) --------------------------MKYFYCIVFFLPIFCCFNIANNKK

Di-NLP-37 (1) --------------------------------------------------

Na-NLP-37 (1) ------------------------------*MVSRAVAPTALLLAILATTL*

Hc-NLP-37 (1) MHIIWRWCVENRMRPSSITTLCPYRFHPLQTQMISRALFTVLFAVSAISS

Bx-NLP-37 (1) ----------MHAIIDYFRAVPSLLGKMQSLVSSFVVLMTITVTVMTSKS

51 100

Ce-NLP-37 (24) *VVDA*TPRSQGNMMRYGNSLPAYAPHV-------------------LYRFY

As-NLP-37 (24) RRSLPAMGNRVIAQFLGRLPLYQGSSN------------KRFDAVMMPFD

Bm-NLP-37 (25) LLMLYPREQVMERLLESLPKRDDEHV-------------------VNTRN

Di-NLP-37 (1) ----------------IFFLKFTKRK-------------------LQIRN

Na-NLP-37 (21) *A*QQERIMGAMPSRYTFYHDRGYEYAP-------------------PLTLY

Hc-NLP-37 (51) ANQQRRMETTPSGYNSYQDKPYSRT--------------------LLSLY

Bx-NLP-37 (41) DALPFPPHTSLVIQDTQLIRQFLDRYQPAVLFHPMVRRYDVQQEDMDDGE

101 136

Ce-NLP-37 (55) NSRQFAPINKRNNAEVVNHILKNFGALDRLGDVGK-

As-NLP-37 (62) TSSDDYTVQKRNNAEVVNHILKNFGALDRLGDVGRR

Bm-NLP-37 (56) DGISAYMVRKRNNAELVNHILKNLNEVDLLGEVGR-

Di-NLP-37 (16) DKISAYTMRKRNNAELVNHILKNLDEVDLLGDVGR-

Na-NLP-37 (52) RKVGARPVSKRNNAEVVNHILKNFGTLDRLGDVGK-

Hc-NLP-37 (81) RKLHDGLISKRNNAEVVNHILKNFGTLDRLGDVGK-

Bx-NLP-37 (91) ERLALRPMAKRNNAEVVNHILKNFGTLDRLGDVGK-

NLP-38

1 50

Ce-NLP-38 (1) --------------------------------------------------

As-NLP-38 (1) TNERRKRLYAYNIPYRTSYHDRREKWTRYQCSEAEYHSMMSSHSSFARHM

Bx-NLP-38 (1) --------------------------------------------------

Gp-NLP-38 (1) ----------------------------MSTQNSKSSAPVFSTKHCALGH

51 100

Ce-NLP-38 (1) -*MQLIHFIVGLAMLISLSLA*ASDDRVLGWNKAHGLWGKR-----------

As-NLP-38 (51) LRTGIVLIATIVFLSAPALAREQDKRDNWNKALGLWGKRSI---------

Bx-NLP-38 (1) --*MVQKSAWQMLILLLLAVFSTIQA*DRQWSAGVGLWGKRS----------

Gp-NLP-38 (23) RFALLVALLLFGLVTISVGAPADGQSHRWSNGVGLWGKRSDGLTNLALLD

101 150

Ce-NLP-38 (39) --SVQEASQDKRTPQNWNKLNSLWGKRSASSFDDDYTTENGDDDVTMLYK

As-NLP-38 (92) SPQFLDTNLEKRSQDQWNKLNSLWGKR------TSWQTANG------LWG

Bx-NLP-38 (39) ----YPLLRKRSPDFNPNGEAGLWDKR-----------------------

Gp-NLP-38 (73) GLFDEARSVRKRPEHSWHKLNALWGKR----------SSNS---------

151 197

Ce-NLP-38 (87) RSNLSPRFLGRMTFARIPKISPAQWQRANGLWGR-------------

As-NLP-38 (130) KRS-----------------S---WETANGLWGKRSLKSEQLDDH--

Bx-NLP-38 (62) ----------------------AQWQAANGLWGKRSVPPVPQYVPWN

Gp-NLP-38 (104) --N---------------------WQTANGLWGKR------------

NLP-40

1 50

Ce-NLP-40 (1) --------------------------------------------------

As-NLP-40 (1) --------------------------------------------------

Na-NLP-40 (1) --------------------------------------------------

Hc-NLP-40 (1) --------------------------------------------------

Bx-NLP-40 (1) --------------------------------------------------

Gp-NLP-40.1 (1) --------------------------------------------------

Gp-NLP-40.2 (1) MASSSAYPILFFTQTWSGDDKLWIFYWLSSESKFGIASFPDTVYEMEADR

51 100

Ce-NLP-40 (1) --------------------------------------------------

As-NLP-40 (1) --------------------------------------------------

Na-NLP-40 (1) -----------------------------------------MSLSAFQTR

Hc-NLP-40 (1) --------------------------------------------------

Bx-NLP-40 (1) --------------------------------------------------

Gp-NLP-40.1 (1) --------------------------------------------------

Gp-NLP-40.2 (51) DTRKAFLGNIAKSLVEEYWETMFEEGKTKIKQMNDESATVSYAFKHFLEL

101 150

Ce-NLP-40 (1) --------------------------------------------*MKLVIL*

As-NLP-40 (1) ------------------------------------------*MYRMMRFT*

Na-NLP-40 (10) SASLKHFDKKACMDELVITKLFEWAEPHQLHTDTQHTKRTHTAIAPMRAL

Hc-NLP-40 (1) ------------------*MWAEHGKTSVLIRPRGWHTGHSRSPTSPMRPL*

Bx-NLP-40 (1) ----------------------------------------------*MKSV*

Gp-NLP-40.1 (1) ------------------MGHRRCCTACTCGAGVCMVNNNDEFCPKSSAS

Gp-NLP-40.2 (101) PQLDDPVKRRQRIDRLVKFFVDEALEKKRMEQGKQPLSLHNRNHPTNASS

151 200

Ce-NLP-40 (7) *LSFVATVAVFA*APSAPAG-LEEKLRALQEQLYSLE---KENGVDVK----

As-NLP-40 (9) *LFAVLLPAVLQAAA*QPLSELEGKFKSLQQQLLNLQ---QEMQLQQQQDKS

Na-NLP-40 (60) IIAVSIGLIFAAPIQDADSVQEKLKALQEQLNTLQ---KQLVVPAS----

Hc-NLP-40 (33) *VIALIFAAAY*AAPVQDGEKIEEKLKHLQEDLAALQ---RQLTAPTT----

Bx-NLP-40 (5) *LLVFGVVLGVVLA*APAGEDLQKKIDAMEKHIQLLE---KALLQRRSPLQG

Gp-NLP-40.1 (33) RCGAAQSNSSNGRQDQPQQQINALRAQMDQLQYELGQLRQYAPAGADWME

Gp-NLP-40.2 (151) LRNNSAKRKQPTNTRKALRKLPTLGLNFDDVIEED---QQRGLQIADWME

201 250

Ce-NLP-40 (49) -----QKEQP------------AAADTFLGFVP------QKRMVAWQPMK

As-NLP-40 (56) VAVPAQEQQPQIHDEYMLPELMAQGIQYGDLRAKPSNTQQKRLVYWQPMK

Na-NLP-40 (103) -----SQNED------------ARKFLFNDWQP------QKRMVAWQPMK

Hc-NLP-40 (76) -----MEKEE------------ARKLLYNDWQP------QKRMVAWQPMK

Bx-NLP-40 (52) SLIEGNQIMN------------PRATRALAFQP------MKRMVAWQPMK

Gp-NLP-40.1 (83) PVWPFSNNVDYHPKSMLVRRHMAWQPMRRGIENDV---------------

Gp-NLP-40.2 (198) PVWPFSNNVDYHPKSMLVRRHMAWQPMRRGIEKDER-SMTQRHIAWQPMK

251 300

Ce-NLP-40 (76) RSM------------------------INEDSRAPLLHAIEARLAEVLRA

As-NLP-40 (106) KS--------------------------VVSTKEQVLQAIESRLAEVIHA

Na-NLP-40 (130) R---------------------------NLDERQPLLHAIEARLSEVLRA

Hc-NLP-40 (103) R---------------------------NLDERQPLLHAIEARLSEVLRA

Bx-NLP-40 (84) RSI------------------------AAEYNKDQVIRAIEEQLLEILHA

Gp-NLP-40.1 (118) --------------------------------------------------

Gp-NLP-40.2 (247) RQQQPEHGMGEQYDGEGPDVAAATVYAAGDGQKQQVMRTVEQELIEVLRA

301 330

Ce-NLP-40 (102) GERLGVNPEEVLADLRARNQFQ--------

As-NLP-40 (130) GEKLGVSADELLAHLKMRNSSPQ-------

Na-NLP-40 (153) GERLGVSPEEVLQDLRYRNQFKK-------

Hc-NLP-40 (126) GERLGISPEEVLQDLRYRNQFKK-------

Bx-NLP-40 (110) GETLGVNAEEVLGDLKKKNGDLM-------

Gp-NLP-40.1 (118) ------------------------------

Gp-NLP-40.2 (297) GERLGVSADEILAHLRARNNNNNAGGLGRR

NLP-42

1 50

Ce-NLP-42 (1) ------------------------------------------*MRVQVVTL*

As-NLP-42 (1) --------------------------------------------------

Na-NLP-42 (1) -----------------------------------------------*MRS*

Hc-NLP-42 (1) -------MQLTLAYGMKASDYLQGMPKGDIKPGWEDLGWAWGKRINMKGS

Bx-NLP-42 (1) -----------------------------------------------*MSL*

Gp-NLP-42 (1) *MLRQCFLRPSCPFVGGGSSLLLLALLALPSAFA*WNSRTFGGLDPNSGGGG

51 100

Ce-NLP-42 (9) *LAVLLAVLQFTSA*AG--NYYSGYPSDRTMKRSALLQPENNPEWNQLGWAW

As-NLP-42 (1) *MCRRLHRLSVAFTSFIVIAFSHEA*LSLPSSAREREMKRNKPHWDDLGWSW

Na-NLP-42 (4) *LQVIVLLIAIIQ------ATFT*YNLKLADALQSMPKREIKPDWEDLGWAW

Hc-NLP-42 (44) SSVFLLFIAFMQ------LTLAYGMKASDYLQGMPKGDIKPGWEDLGWGK

Bx-NLP-42 (4) *LTRILCTLTVVG------ITIAITHA*SPMDRSALKKASDNPQWEDLGWAW

Gp-NLP-42 (51) GTRRLYMLKKSESPQWEDLGWAWGKRSGGPNSAAPSPQNSANWDDLGWAW

101 150

Ce-NLP-42 (57) G---------------------KRSAGMEIP-HRAARALHPVKK-NPDWQ

As-NLP-42 (51) GKRALGGSVSPQWESSGWTWGEQTFPLQESHTQRALRVAMTKKN--PDWH

Na-NLP-42 (48) G---------------------KRSVPDSVYPQRFLRPMQLVKK-TPDWH

Hc-NLP-42 (88) ----------------------RSAVGELSYPRRFLRSMQLVKK-SVDWH

Bx-NLP-42 (48) GKRSVA---------------VELVDPEDVMLQRYVRSMKAIKK-NPDWH

Gp-NLP-42 (101) GKRSPAEGGEAARR-------AKAHREDPIVSMMSNRVVRSMTKQSPDWH

151

Ce-NLP-42 (84) DLGFAWGRK-

As-NLP-42 (99) DLGWTWGRK-

Na-NLP-42 (76) DLGWTWGRRK

Hc-NLP-42 (115) SLGWAWGRRK

Bx-NLP-42 (82) DLGWAWGK--

Gp-NLP-42 (144) DLGWAWGRRK

NLP-43

1 50

Ce-NLP-43 (1) ------*MSLAQSTFYLLFVAFLAVVIAVTA*DKQQSAYDLETPISAYKRFY

Ts-NLP-43 (1) *MSSVKSCICIFTAFCLLASFTSA*TLVDSPSSAKEVQMIPVFYSDTVGDHH

Tm-NLP-43 (1) ---------------MEIQSGFGESPKLPQGAQSWDMDKEFLQNLGPSAL

As-NLP-43 (1) ----------*MHVQCAAAIFLLITVQLLISTVSA*QDTPLVFKANQMKRFY

Na-NLP-43 (1) ----------*MQRLLLVIATIFSVLANCQG*EQQHSVENRYFTDDGITGDY

Hc-NLP-43 (1) --------------------MLAVLVSCQREQLSPEMSRYFMDDIGSSEY

51 100

Ce-NLP-43 (45) SWEDAKRAASSEEG----MRNKRKQFYAWAGKRSSAPVHYFEDSIAQEAG

Ts-NLP-43 (51) VIDPTVDNLEDVSELN---NLMKRKFYAWAGKRASRFYQWAG---KRNGE

Tm-NLP-43 (36) GTIRRPAYPDDPEDQMGHYQLLKRKFYAWAGKRSPAPRFYQWGGKRSDDV

As-NLP-43 (41) SWEETKRSPSEDVQPP--SDYTKRKFYAWAGKRS----------APISDS

Na-NLP-43 (41) IPYDMKGFVT-----------KRKQFYAWAGKRS---------------V

Hc-NLP-43 (31) VPSLMKGSDS-----------KRKQFYAWAGKRS---------------H

101 150

Ce-NLP-43 (91) PSMEKRKQFYAWAGK-----------------------------------

Ts-NLP-43 (95) KVEKVRRKFYPWAAKCNKKTYEVAKDIQNRTTILFIFAITYLYCTLTVQN

Tm-NLP-43 (86) SDEKVRRKFYAWAG------------------------------------

As-NLP-43 (79) VAYNKRK-FYQWAGK-----------------------------------

Na-NLP-43 (65) PVIDKRKQFYAWAG------------------------------------

Hc-NLP-43 (55) PPYEKRKQFYAWAGK-----------------------------------

151 161

Ce-NLP-43 (106) -----------

Ts-NLP-43 (145) LQKACKILRIR

Tm-NLP-43 (100) -----------

As-NLP-43 (93) -----------

Na-NLP-43 (79) -----------

Hc-NLP-43 (70) -----------

NLP-44

1 50

Ce-NLP-44 (1) ----------*MLLWIVATLLIFSLPVSTA*LDYNDFSLQRIARAPHPSSAL

As-NLP-44 (1) --------------*MATLAVTFFALLLTVFVSG*NYPKQRRNDRPQSSAAV

Na-NLP-44 (1) MEALLDQDSRQTQEERGMKNRSTPTTQSAENLDVFPLQRISRSQLALSTL

Hc-NLP-44 (1) ---------*MKLTLTLLLVIIFFRVNA*EHSDVDMFPLHRISRSTLPLSSL

Bx-NLP-44 (1) --------*MKNAAVAVLLCLCLVYVSS*QELFSERFAVPKPIGVPVEASQL

Gp-NLP-44 (1) ---------------MSVVAGRKVLIDPYEWPRVLAARKAQRPPTDSWNY

51 100

Ce-NLP-44 (41) LVP----YPRVGKR-SNILNNNSESQNS-----VQKRLYMARVGKRA-FF

As-NLP-44 (37) LVP----YPRVGKRFDTIAATYPYSSDE-----NSQSSSSPRAKQPV-RL

Na-NLP-44 (51) LVP----YPRVGKRSTNGTDHQVSAPIL-----HAKRLFAT-VGKRT-YM

Hc-NLP-44 (42) LVP----YPRVGKRSFFTGDRNVYPPTS-----IRKRLYVARVGKRR-YL

Bx-NLP-44 (43) RSRGSDVYVTPGKYPSRVYGLFKRAADQPAPEFQVKRMYVARIGKR--QV

Gp-NLP-44 (36) DPPWYDDIFTRGEGMHKRMSSMDGGGGEGEDGSMGKRLFVARIGKRMAHG

101 134

Ce-NLP-44 (80) YTPRIGK---------------------------

As-NLP-44 (77) EARSPHRWVGGGCTYPFTDLSSVHNAAPLNVASD

Na-NLP-44 (90) YAARVGK---------------------------

Hc-NLP-44 (82) YTARVGK---------------------------

Bx-NLP-44 (91) YRARVGK---------------------------

Gp-NLP-44 (86) YWARVG----------------------------

NLP-46

1 50

Ce-NLP-46 (1) -----------------------------*MLSIRTFVLVLLVLVGLAAA*L

As-NLP-46 (1) -------MQYGYSIKVLLTTTLIALVISLPVHDADYSSKDQYRALEETPI

Bm-NLP-46 (1) -----------------*MVDVSYTLVLLLATFIKFAQG*LPIFHETNYEPF

Na-NLP-46 (1) ------MLLVPYKDYGATESAKTITVKSYMTTVIGYVRSLVFFLLIGLSL

Bx-NLP-46 (1) MEKCPMVDDKVRADFGALASAAYCWVKIRRKFVWTMSQALEAALLCVVLI

51 100

Ce-NLP-46 (22) PYFRLNYDYDP------------------------VDLDNDEKNAARQFL

As-NLP-46 (44) LYPANILFLRG-----------------------------DEYADFISPR

Bm-NLP-46 (34) ANYLNSLDFDQP------------------------KLSMYEPLPLPILD

Na-NLP-46 (45) ALPLLWLDVEG------------------------GDDQQYQQWRGIGPL

Bx-NLP-46 (51) SVHSLPILMNGQPMEMMYIDGPHDMFLPALDTEGPESAWSWFDQTQSSHP

101 119

Ce-NLP-46 (48) PYMKRNIAIGRGDGLRPGK

As-NLP-46 (65) AG-KRNIAIGRGDGFRPGK

Bm-NLP-46 (60) VMVKKNIAIGRADGFRPGK

Na-NLP-46 (71) TMVKRNIAIGRGDGLRPGK

Bx-NLP-46 (101) VAQKRNIAIGRGDGFRPGK

NLP-47

1 50

Ce-NLP-47 (1) ----------------*MQLYVVLCFLVLLGLSAG*QMTFTDQWTKKR---A

Ts-NLP-47 (1) -------*MQFIFKEAVTVSWCTLLLAFLVSQTAS*QLTFSSGWGKRTTAEP

Tm-NLP-47 (1) -------------*MSHTSLRVTILLVFVICGAVA*QLTFSQGWGKRTTDAL

As-NLP-47 (1) ------------MFLTRHNLILIAIVELLRMGTAQITFTDQWTKRS-GIS

Bm-NLP-47 (1) ------------------*MRRITLLPAIIYIVTA*QMTFTDNWDKRSLSNL

Di-NLP-47 (1) ------------------*MRRVVLLLTVIYIVTA*QMTFTDNWDKRTFPSL

Na-NLP-47.1 (1) ---------------*MRSVLVSLIVLLLVPSSFS*QMTFTDQWSKK--SEP

Na-NLP-47.2 (1) ------------------*MRALLVLLAFLALTFA*QMTFTDQWNKRSAPYE

Hc-NLP-47 (1) *MAQNHSIQRRKHGSNMRFVLVTIIVALCIGISLS*QMTFSDHWSKKSLPGP

Bx-NLP-47 (1) ------*MDLVCRSLTNTMQTIMILLLVSVTMTSA*QMTFSDGWGKRSSPLK

Gp-NLP-47 (1) -------------*MPSLVFPTLCFFVSAVVLA*NSQMTFSDGWGKRS-AVA

51 100

Ce-NLP-47 (32) TLHKQLPVVTPE------------------------EPICPSDRVQAVFE

Ts-NLP-47 (44) IGKASYCAHLDQD----------------------ILQLIQQIITVHISF

Tm-NLP-47 (38) ILKNANLDCESIN---------------------EEAMKIVRRLVEVSFF

As-NLP-47 (38) PIPLLKDLSQRG------------FASEAIPNATAFHMSFHAEYTANCSI

Bm-NLP-47 (33) FRGWQQDDLMVN-------------------------RACSWDTIAEITR

Di-NLP-47 (33) FRGWQRDELTIN-------------------------EACSSDVIIEIIK

Na-NLP-47.1 (34) PFPRYKPLLRQD--------------------------------------

Na-NLP-47.2 (33) PLASLKTTGRSS--------------------------MCGHRAIDEMLL

Hc-NLP-47 (51) AFPRYKPRESESSSQQKPAQ---------------QQQFCQEEKVTDALA

Bx-NLP-47 (45) PFSAGDDSGVNS-------------------------NPLLDKCNTEYSQ

Gp-NLP-47 (37) PFALLADRHMTEPTKRQQKSKTNANFFVSPDEDTLTMSSEMEACHAGYAQ

101 135

Ce-NLP-47 (58) QLDQLQKAQQRLTEYLASCAYPVEVPQKAEKM---

Ts-NLP-47 (72) ENFFIQFIYSYFSFLFILFSFSIRNITFNIKNA--

Tm-NLP-47 (67) RGIILLCMQLMLQDYRRSYRICQQQHMRRLFGRK-

As-NLP-47 (76) TLESSFITQIFIDQQVMRMKDAKRPFSSQWWLS--

Bm-NLP-47 (58) ELKQIYKKQNELINLIDRCSIIERPVFVKEG----

Di-NLP-47 (58) ELKHIYKRQNELINVIDRCSIINRPVFIKEGKQKF

Na-NLP-47.1 (46) -----------------------------------

Na-NLP-47.2 (57) QLVQLQSAEKEIIAELNACLPAKRQ----------

Hc-NLP-47 (86) QLAQLGEAQNMLTSYLNTCYQSRPRV---------

Bx-NLP-47 (70) TLVQLHHRIMKLYDTYEQCQTRALNGVATHRQQ--

Gp-NLP-47 (87) RLIQLHEQMLSLFSTYQQCQTKAAIETKKSMRQS-

NLP-48

1 50

Ce-NLP-48 (1) *MMSSRRIVYTLLLITIALCAYSTAYA*PRNIRGVGDVPSMFFSPFRMMGKR

Na-NLP-48 (1) *MSRSRVLVFTFMALFILAAVVDS*ATIVR-----RSIPEVFNSPFHVLGKR

Hc-NLP-48 (1) *MLALRVFVLTIMLAMVFNVIA*ESDRVIR----SGPLYNAFNVPFRVIGKR

51 100

Ce-NLP-48 (51) NQFYGLFKKIRSNEVVDY--------------------------------

Na-NLP-48 (46) NQFYGLFKPFCERRVNDFYKKKKLLSKLFEVNYSQRMSCTHGLLGVLVMT

Hc-NLP-48 (47) NQFYGLFKKLNYESSKPF--------------------------------

101 131

Ce-NLP-48 (69) -------------------------------

Na-NLP-48 (96) FFFLNITLTPLGAVPGTAPGVACGGLLMTRT

Hc-NLP-48 (65) -------------------------------

NLP-49

1 50

Ce-NLP-49 (1) ------------------------------MMKWLLLAVFCIA----AYA

Ts-NLP-49 (1) MGNNQRILSANDPKTEDIHWLNSNQLAELQMNITVIALVLTTLCVIVVHS

Tm-NLP-49 (1) --------------------------MTLFKNLALLSLLLSTLYTSSVLS

As-NLP-49 (1) ---------------------------------------------MQFYR

Bm-NLP-49 (1) ------------------------------MKLSVTLLIVLVTDCIAWPK

Di-NLP-49 (1) ------------------------------MKLSIALLIVFITDCIAWPK

Na-NLP-49 (1) ------------------------------MMKWTLLSVLALVCILNAFA

Hc-NLP-49 (1) ------------------------------MMKWTMFAVLVLACIMNAFA

Bx-NLP-49 (1) ---------------------------MMKLWVSVLALLAIVSAVLAYPQ

Gp-NLP-49 (1) -----------------------VAPMRRSLPSFLPFRFLLLPLLFLIVS

51 100

Ce-NLP-49 (17) WADGTEDSNIDQLMSRVYRTVLLKSKRS----------------------

Ts-NLP-49 (51) EPNLYDWYQNSGSLERFLQELREKRKRSRISKYSQNQESIRHRGNVLSST

Tm-NLP-49 (25) QRNPYEWIQPSQSFEKMIEQLKEKRK------------------------

As-NLP-49 (6) KTTLKHNDDEEAKRASCYSVSTFWLSHIFIA-------------------

Bm-NLP-49 (21) SIDRTGNDLPIEQLSRIYQAMLLKSKRS----------------------

Di-NLP-49 (21) NIDRTDNDLPIEQLSRAYQPILLKSKRS----------------------

Na-NLP-49 (21) WADASEDSNMDQLMARVYRTVFLKSKRS----------------------

Hc-NLP-49 (21) WADGVEDANIDQLMSRVYRTVLLKSKRS----------------------

Bx-NLP-49 (24) TPSEFDAEFARQLIGRAYSALAKTKRSRE---------------------

Gp-NLP-49 (28) NSAAPTIDSDLIPPARFYRTLWSFNPKRS---------------------

101 144

Ce-NLP-49 (45) ------PSMGLSLAEYMASP-QGGDNFHFMP-SGRK--------

Ts-NLP-49 (101) VHLERLPSIGLSLAEYMADP-ERSQDFHFLR-GRKKRMIQFFNS

Tm-NLP-49 (51) ------PSIGLSLAEYVADP-TRADNFHFLN-GRRKRSTESGS-

As-NLP-49 (37) ------ASMGLSLAEYMASP-QGQDNFHFIP-SGRK--------

Bm-NLP-49 (49) ------PSMGLSLAEYMASP-QSQDNFHFIP-IIRR--------

Di-NLP-49 (49) ------PSMGLSLAEYMASP-QGQDNFHFIP-IIRR--------

Na-NLP-49 (49) ------PSMGLSLAEYMANP-QGGDNFHFIP-SGRK--------

Hc-NLP-49 (49) ------PSMGLSLAEYMASP-QGGDNFHFIP-SGRK--------

Bx-NLP-49 (53) ----FKTSMGLSLAEYMASP-QGGDNFHFIP-SGRK--------

Gp-NLP-49 (57) ------PSIGLSLAEYMAAGNQGQEQFHFIPGSGRRK-------

NLP-50

1 50

Ce-NLP-50 (1) --*MRFSVFVALFALLAVSYG*FMYEMED-SYPVSGELPKRSLENFWRNIHL

Na-NLP-50 (1) *MRALRELLVLLLAILATTQA*FMIDGDEYSGLVDYPLPKRNFQHIWRNIQM

51 77

Ce-NLP-50 (48) QMPSGRGGQKRTEGLSRASANAYYRLG

Na-NLP-50 (51) QMP---GSQKRTESLSRARANAYYRLG

NLP-51

1 50

Ce-NLP-51 (1) ------------*MRFLILALLVLFAITQA*YPSSDYQPRYRKS------QT

As-NLP-51 (1) *MRMPQVRIIAVSLVLLVLVDA*LPSLQYPYYSIQQLQPRYRKSS--SANQP

Hc-NLP-51 (1) ------------*MRILLFCLALLLSVCGF*H--SEAQTRLRKS------HA

Bx-NLP-51 (1) *MSRLTVLLLIAFMCTMLMVSVRP*SPIHQQPLNTDVYLRFRRNAQNSRPTR

51 89

Ce-NLP-51 (33) QEANIQPFIRFRKSTQPQHNWMFRPDVAPYFE-------

As-NLP-51 (49) HDSMVQPFIRFRKALSNGFHSWFDEVTPSLAYLYAESRN

Hc-NLP-51 (31) QDANIQPFIRFRKASYGSWIDPLNAYH------------

Bx-NLP-51 (51) ADEYVQPFIRFRKNYQLPYDVSFDYVPYMTRI-------

NLP-52

1 50

Ce-NLP-52 (1) *MFRTLLICFFLMAAVSITSA*YILDYDVPERQTRGDVKSVFFSPFRMVGKR

Na-NLP-52 (1) ----*MSRTSFVIIVAFICLVLIAHVQS*RNLAMTGGVSSLFNSPFRVIGKR

51 76

Ce-NLP-52 (51) SQFYGLYKQLGHSKRETSLE------

Na-NLP-52 (47) SQLYGYYRQGNLDFPQVPLAIDQFDY

NLP-54

1 50

Ce-NLP-54 (1) *MMNYSVVLLLFALCSIVAA*SPMWFDE---EEPMNLRAFRVMPNQLDSIDA

As-NLP-54 (1) ---MNNKARCNDITGRELFGKRSLDASTNEYDALDAVHSVEGSTSISSSY

Na-NLP-54 (1) *MQLLLLFTMVALIVTVVSA*EPRLRFSNSAESGLLRRAGLFFRKRGSAWDG

Hc-NLP-54 (1) -*MNLLLLLCTLGLVALTAA*EPRLRLS-SAEEDLVRRAKVLLKKKGNMWGT

Bx-NLP-54 (1) *MHAIISFALFATIAAGMSA*QDMELNGDFSQHDYRMLPVQREMMRENVGET

Gp-NLP-54 (1) *MPILFVLIGAKIVIG*GLDVEEWDFPTDRLEAFQKRKVRELFGKR----ST

51 100

Ce-NLP-54 (48) SRRLLKRASMFN---------------KRRGRELFGKRSLPIEYP-----

As-NLP-54 (48) TTPSEQQQRLFEFVPSMADDAHG----AGINLEVNRERMPAVEFLKLLAA

Na-NLP-54 (51) VSAPFTFTNLAE---------------KRRVRELFGKRSAPAVETPDVNV

Hc-NLP-54 (49) PSKSYGYTNLAE---------------KRRVRELFGKRSAISATPENE--

Bx-NLP-54 (51) LPTLHFLRSIRRSRELFGKRSPSVVEEKRRSRELFGKRSAKSEE------

Gp-NLP-54 (47) PPAAIPYERHSR-----------------RTRELFGKRADPFAESNDEPL

101 150

Ce-NLP-54 (78) -------------------------------------------DETVMYE

As-NLP-54 (94) TSGPK----------------------------------IVLQNRAALGM

Na-NLP-54 (86) LELPD-------------------------------DQYPSLYDSMLELH

Hc-NLP-54 (82) -ALSD-------------------------------EQYQPVDDLLMELH

Bx-NLP-54 (95) -EIPF-------------------------------NSQEVLALATLLTE

Gp-NLP-54 (80) MAVDLPEEDEHRIKMPRTVFARLRTFQNRANAVEEEQEAPFSTEGLWLLQ

151 163

Ce-NLP-54 (85) SRFRRRANELFG-

As-NLP-54 (110) RHRRGRSRELFG-

Na-NLP-54 (105) RDRRGRVRELFG-

Hc-NLP-54 (100) RDRRGRVRELFG-

Bx-NLP-54 (113) RPRRARGTELFGK

Gp-NLP-54 (130) RPRRGVHRELFG-

NLP-55

1 50

Ce-NLP-55 (1) --------------------------------------------------

As-NLP-55 (1) --------------------------------------------------

Na-NLP-55 (1) MTTNELATCDASHSALGHYSNQDFLSMEPVYLLVYHDVKKKRCNYVVETD

Hc-NLP-55 (1) --------------------------------------------------

Bx-NLP-55 (1) --------------------------------------------------

Gp-NLP-55 (1) --------------------------------------------------

51 100

Ce-NLP-55 (1) --------------------------------------------------

As-NLP-55 (1) -----------------------------------------MKAPWIIYI

Na-NLP-55 (51) EERISTRITRDHTEARQRLVLRWYCVKSCVRFGCGSQGVELIPGSAVYGT

Hc-NLP-55 (1) --------------------------------------------------

Bx-NLP-55 (1) ------------------------------------MVSVSVIILQLITA

Gp-NLP-55 (1) -------------------MATLSEDFRRHNQCSIRNMHRTWLLLLCATV

101 150

Ce-NLP-55 (1) --------*MSCISMLFILLVACLLVSNA*MYINPDYY---YVEQLPTMKKS

As-NLP-55 (10) TVLAISTGRINGHLMERFMGNRANLNDATFILPDDS-PSSSHSILFTKKS

Na-NLP-55 (101) APHAMNGSLSAIIVRVLLLCALVALSSGYYLAYKPS-----KNGLLPIKK

Hc-NLP-55 (1) ----*MNSSLSAAIVRVLLLCALVALCNS*YYLAYEPNGASGYEGAWRAAKK

Bx-NLP-55 (15) FLLVVDGYIIDRWSTLEDPARLYPLSDSEYIRYPVRRSVDTEEASMDRTF

Gp-NLP-55 (32) FFMSIASNQVHGYLIDGRSLQKMMAVQRRWAQRPAKLEEFSKFVPASKKS

151 200

Ce-NLP-55 (40) GQLRA---------------LAGSRNCFFSPVNCIITHDINSYRRLAKGS

As-NLP-55 (59) LRQFQ---------------GSGSRNCFFSPIQCVLQHDLSKYRKLVDST

Na-NLP-55 (146) SQIGG---------------LVGSRNCYFSPVSCVITRDVSAYRKLAGPS

Hc-NLP-55 (47) SQLRA---------------LAGSRNCFFSPINCVITHDVSTYRKLAGTR

Bx-NLP-55 (65) SQGYAKKSQNLSYKALKKFSGSGSRNCFFTPIQCMIQHDMSKYKKLVDGS

Gp-NLP-55 (82) AGYGVLSKFG----------GSGARNCYFTPVSCMIQHDMSKYKKLIESS

201

Ce-NLP-55 (75) SYA------

As-NLP-55 (94) NYKLNV---

Na-NLP-55 (181) ---------

Hc-NLP-55 (82) ---------

Bx-NLP-55 (115) KVIQN----

Gp-NLP-55 (122) RLDVFANLR

NLP-56

1 53

Ce-NLP-56A (1) -------------------------*MPSPSSLLGSLLLVCAVLTITSRA*SS--

Ce-NLP-56B (1) -------------------------*MPSPSSLLGSLLLVCAVLTITSRA*SS--

Ce-NLP-56C (1) -------------------------*MPSPSSLLGSLLLVCAVLTITSRA*SS--

As-NLP-56 (1) MPGKLKEDFRERPEVLSTSYLSAFLFLRLHYKYHLIKLLLSYVHVVEDLMESG

Na-NLP-56 (1) ---MFLAEKPQGVDIRDANQKKFEAKGKYVMRLWLNTLLAAMLLVIVTALEP-

Bx-NLP-56 (1) -------------------------*MCSRFSWALLIVGMALAIHLASG*VPSP-

Gp-NLP-56 (1) ---------------------------TEMAEGYVISSYQTKLLPPKGFFPS-

54 106

Ce-NLP-56A (27) -----------------IMTDDVEPPQLLTRQLRSFPYSVSFYRMLG------

Ce-NLP-56B (27) -----------------IMTDDVEPPQLLTRQLRSFPYSVSFYRMLG------

Ce-NLP-56C (27) -----------------IMTDDVEPPQLLTRQLRSFPYSVSFYRMLG------

As-NLP-56 (54) VMYWLGLCSVLLWLHASVFSTVQSSEDINFRTARGFPQTTSFVQLLQPDEG--

Na-NLP-56 (50) ----------------------FEDEFLKERPTRAFPFLLRITPLIG------

Bx-NLP-56 (28) ----------------KAKSDKQKLFRVARMTFQSFPYSLAYMRMMRPAPSSI

Gp-NLP-56 (26) ----------------STIAYYRSMKTLLQRPNKIVPNHVGDSEGHRP-----

107 159

Ce-NLP-56A (57) ---HDRQLRPYYGVNDEVAALIDSMNSDNVANEDVFPTRPR-RSDGLRGSFYW

Ce-NLP-56B (57) ---HDRQLRPYYGVNDEVAALIDSMNSDNVANEDVFPTRPR-RSDGLRGYACR

Ce-NLP-56C (57) ---HDRQLRPYYGVNDEVAALIDSMNSDNVANEDVFPTRPR-RSDGLRGSFYW

As-NLP-56 (105) RKWRSYNGAVAVRLSSDLNDLINQIEAEQMMGRRRRSKDIG----KYRRYACR

Na-NLP-56 (75) -----YDRRYGSKVDKVPLSTEQIDSTDGVIARMRRDGHML------KRYTCR

Bx-NLP-56 (65) RPLASYQWLHKAEADNELAELIDSIDGQAIGDRERRSTGP-----VYKRYACR

Gp-NLP-56 (58) ---NGREMTEWVKVFNARAQKGRDRRSEGEAIKREFSISFKNFKIRNLGYTCR

160 171

Ce-NLP-56A (106) ARIRLPLQIL--

Ce-NLP-56B (106) FKFCRIYDA---

Ce-NLP-56C (106) ARFVVCQPVLVI

As-NLP-56 (154) FKFCRIFD----

Na-NLP-56 (117) FKFCRIFDE---

Bx-NLP-56 (113) FKFCRIFDA---

Gp-NLP-56 (108) FKFCRIFSPTDE

NLP-57

1 50

Ce-NLP-57 (1) -------------------*MRFILGLLIAIVAFVAS*SPIHGIWNNLPAPP

As-NLP-57 (1) ---------------------FLEGKRNSQAVLINEMTERSSTSKSELFQ

Bm-NLP-57 (1) -----------------*MKQIFSFALISLLLNLLHA*SPIASIWGNLPDSA

Na-NLP-57 (1) MMNIQVWTFQRRIREAVGKVLFLRDIGSPRGYRTNGKERQDGNDMVLKEF

Bx-NLP-57 (1) ---------------*MSRSLALLFALFMAAVIMVHC*APIDS----LPEME

51 100

Ce-NLP-57 (32) QK----RVYGFYNYLPKEEDDRDKRNTILLLTPNEDYVE-----------

As-NLP-57 (30) DK--K-SIYGLYRHLPGYYDDDTVEKKSAFFGIPLNEME-----------

Bm-NLP-57 (34) AQSMKRSIYGLYHHLPGYAYNEQPEPEIYVLPPAYRSWTN----------

Na-NLP-57 (51) EATIQKKKYGYYVRLPWKKDSADLPDNKAMSFRRLQTIVAKLQKNPELMQ

Bx-NLP-57 (32) KK----SIYGLYQYIPKASGVKS-KKFSSDPGPMIGQLPDGSYVIFGY--

101

Ce-NLP-57 (67) ----------

As-NLP-57 (66) ----------

Bm-NLP-57 (74) ----------

Na-NLP-57 (101) QYHNTFMNQL

Bx-NLP-57 (75) ----------

NLP-58

1 50

Ce-NLP-58 (1) -------------------------------------*MISKCSVMGLLLL*

Ts-NLP-58 (1) ----------------------------*MLVLLGVVMVGFSEL*QVAPLLE

Tm-NLP-58 (1) ----------------------------------FLQQSLSERDNDVAYL

Na-NLP-58 (1) MVERRARVGLGENCHKELLEYHTKQPFKQAFLSSGIHAAFLADLKNLKKK

Hc-NLP-58 (1) ------------------------------------------------IV

51 100

Ce-NLP-58 (14) *LFVHITTA*QFETHFDDAQVYWPSQYKRSGPSSAS----EGEAYAFPGLRG

Ts-NLP-58 (23) NEYDLSSSSSSSSWPGLAKSFKILYSNDNSNMVPNQLWTIVDRRSVPFFG

Tm-NLP-58 (17) DQTYPSWNFGFPRWFDGETYQPVQLSTINR-------------RSVGFFG

Na-NLP-58 (51) VAREQTAFCGRSQQYPYLDLVPEYVKRSDF--------VDAGPTFRGLRG

Hc-NLP-58 (3) IAVEIETTCIFFQQYPYLDLVPEVYKRSLYG-------VDDGFTFKGFRG

101 148

Ce-NLP-58 (60) LRGKRDPTYHKRVPMMSLKGLRGKRARIFDGQEEQ-------------

Ts-NLP-58 (73) VRGK-------KVPFFGARGKRLRPVVIGAGEDERYKKLSMDFFGPRG

Tm-NLP-58 (54) SRGK-------KVPFFGSRGKRLQMDNVHESYQGDGKNKKLDFFGVRG

Na-NLP-58 (93) LRGK-------RMPYMNLKGLRGKRML---------------------

Hc-NLP-58 (46) LRGK-------RMPYMNLKGLRGKRTF---------------------

NLP-59

1 50

Ce-NLP-59 (1) --------------*MSALHIARLFLLIAIMSTVVSTYA*VLPS-------G

As-NLP-59 (1) *MVIAQILFALAISSPVMC*FLEPLPSLIDTSEGNAATSTTLQQPKFFSIAP

Na-NLP-59 (1) -----*MAYCSTPGTMFVQLAVIWCVLIAQAFS*QPSSSDVLLQRLHYINTP

Hc-NLP-59 (1) --------------*MIVHVASVWCALVAQALS*LTIPSDELEKRLQYMEVP

51 100

Ce-NLP-59 (30) EILFRRPAS--------------------------LFDRAADYSDDVAKF

As-NLP-59 (51) YLRYVNAFRENQLEHLNDNLGNMNARVIYSIPKHFDDDPTWKMMDIDQQQ

Na-NLP-59 (46) VVRYIAEN--------------------------EGLRKSLRGLNDAMRQ

Hc-NLP-59 (37) IVQFVEGREALKENFRRMHPEYMEVPIVQFVEGREALKENFRRMHPELRQ

101 136

Ce-NLP-59 (54) DESRDKRELDGIDTQLSKRTNLKRLVILSARGFGKK

As-NLP-59 (101) MNINRRSLSRFIGASNEKRNALQRLAMNGARGFGRK

Na-NLP-59 (70) KRFEGVQDYTSLEDPYMKRTSLKRLAILSARGFGRK

Hc-NLP-59 (87) KRFEGLADYVALEDPNAKRSSLKRLAILSARGFGRK

NLP-60

1 50

Ce-NLP-60 (1) --------------------------------------------------

Ts-NLP-60 (1) MLAKYVQVILISRAGTALASLRQRASITQPQWTTAIASIIRIKSRNVGGH

Hc-NLP-60 (1) --------------------------------------------------

51 100

Ce-NLP-60 (1) -*MSSSSSTFISIFIITMVLVAFCSA*NLATGRASLRPSAGKRAAVVGRLPA

Ts-NLP-60 (51) SRMSVMFILLSRFDRFSSLLGPANLNLATPIAGCRPSTGRREQLLKTPEQ

Hc-NLP-60 (1) ------*MYMHSQCIILCVLVACGSS*NLFVGRMSTRPSAGKRSTGG-----

101 150

Ce-NLP-60 (50) HYYQLASAVRSPKQYENEFTCNENTMSVINARYQKLQEELNGLVELMEAC

Ts-NLP-60 (101) AG-KQADGLKSLLECKGGRQISALKHLSMLAGYERRSKTR----------

Hc-NLP-60 (40) -----LEPFRRIFQNSGQFCISGIVLNYALTRF-----------------

151

Ce-NLP-60 (100) QTLQSTISL

Ts-NLP-60 (140) ---------

Hc-NLP-60 (68) ---------

NLP-61

1 50

Ce-NLP-61 (1) --------------------------------------------*MSHWIA*

As-NLP-61 (1) ------------------------------MGFAVQKTFGTSFIPSRKLL

Bm-NLP-61.1 (1) *MEQTATVCILKSSNVGTYVSIVQVKRNFFSLLFEEITLSKFCMLLAMKVA*

Bm-NLP-61.2 (1) ------------------------------------------*MLLAMKVA*

Di-NLP-61 (1) ----------------------------------------------*MKMT*

Na-NLP-61 (1) ----------------------------------*MRNVIQMKSLMGVLFV*

Hc-NLP-61 (1) -----------------------------------------------VIS

Bx-NLP-61 (1) --------------------------------------*MFTVTAFRTMFL*

Gp-NLP-61.1 (1) ----------------------------*MLSIFREKNNGGSSHLSTAFIG*

Gp-NLP-61.2 (1) -----------------------------*MATSKNDSSRQITPLAAFAFL*

51 100

Ce-NLP-61 (7) *TLLAFILIANAFA*ALSTGADVGASDDVRSYVMPFYG--------------

As-NLP-61 (21) QFSTITIMLSNDTHVKFLFAGSPSRAVPVAAFERYPLLTIEDIGFHP---

Bm-NLP-61.1 (51) *SSLFLIFVFLWTSVVS*KSIG---QIEAPLMEPMR----------------

Bm-NLP-61.2 (9) *YSLFLTFVILWTSVVPKSIG*---EIEAPLMEPMR----------------

Di-NLP-61 (5) *YTLVFVFVFLWASVIP*QSMEKSNQIEIPIMELNR----------------

Na-NLP-61 (17) *TALLVLFGLA*TPKPIDINEECGPFVGSRKIKFTKS---------------

Hc-NLP-61 (4) SSSMEVFASTFNHIYNLDGS----HISRILNFS-----------------

Bx-NLP-61 (13) *VVFVVFVCISFSSA*LPAPAGYGYDLAPSAFEFPQLN--------------

Gp-NLP-61.1 (23) *LFLAALFVARTAG*QSDSSTAIEYEIVLPVSAADMSIGPRAWQGEWVR---

Gp-NLP-61.2 (22) *AIMAALMSSAET*AAMPQSHGVSRMVLVGEDQQPSLFLLLGPHGWVQQQPM

101 150

Ce-NLP-61 (43) -------------------------------------MEKKWSRREPSIR

As-NLP-61 (68) ----------LELAR-----------------------SKKWTRLEPSIR

Bm-NLP-61.1 (82) --------------------------------------YKKWTKLEPSVR

Bm-NLP-61.2 (40) --------------------------------------YKKWTRLEPSVR

Di-NLP-61 (39) --------------------------------------YKRWTRLEPSVR

Na-NLP-61 (52) --------------------------------------QKKWSKLEPSIS

Hc-NLP-61 (33) ---------------------------------------KKWSSLEPSIR

Bx-NLP-61 (49) -------------------------------------MAKKWSRLEPSIR

Gp-NLP-61.1 (70) ----------RFSPRVVQNGWKYRTAPRNDFGAHRIVMEKKWSPLEPSIR

Gp-NLP-61.2 (72) VFGGSAAPRNRFLPPLSSGTSYRGNKVGEDSGVAEGKVEKKWSRFEPSIR

151 200

Ce-NLP-61 (56) FFKRNGGGQDLPPSRFLWDY------------------------------

As-NLP-61 (85) FIGGSNPYFI----------------------------------------

Bm-NLP-61.1 (94) FLTNDEANERKPSRKAKHIDTNTLYNRTYISSIYACQPALLNKVLFNNLT

Bm-NLP-61.2 (52) FFDKRWTRLEPSVRFFDK--------------------------------

Di-NLP-61 (51) FFDKRWTRLEPSVRFFDKR-------------------------------

Na-NLP-61 (64) REGVQRASHISRRCCPV---------------------------------

Hc-NLP-61 (44) --------------------------------------------------

Bx-NLP-61 (62) FFKRSQFDSDFDN-------------------------------------

Gp-NLP-61.1 (110) FL------------------------------------------------

Gp-NLP-61.2 (122) FF------------------------------------------------

NLP-62

1 50

Ce-NLP-62 (1) *MVKFFVFLLFFAFLCSFTSA*IPLRSLFLRSYDDINQESIARGYFAPQPVD

As-NLP-62 (1) -------MKSNTTVCSKKYWLHQEAIFTPVERANEGKRAES--DRQMRLY

Na-NLP-62 (1) -------*MWPLRVLCLCLLAIGSIHG*FELFQPYWGYNPRTLIRGSDPDFN

Hc-NLP-62 (1) *-------MWPLRVLCFCVLLLVSAHA*FEGYKPHLAYYRQ-----SEPEVY

51 85

Ce-NLP-62 (51) DDNADNRPKRGIDLLKRRVEIIERNRCFFNPITCY

As-NLP-62 (42) LHRASRLPPRIRKHVT--RSQVAKMRCFFNPISC-

Na-NLP-62 (44) LENVRPLWKRQSAEQSKRREPLSRSRCFFNPITC-

Hc-NLP-62 (39) LEENHPLLKRHVIEVK-RKEALARARCFFNPITC-

NLP-63

1 50

Ce-NLP-63 (1) ------------*MLVSLLICLALVAVASA*MPFYASSNDGDVVRQFPQQGV

Na-NLP-63 (1) -----------------------YYSQNFPCFQMVDSNDSAGLRVPLQDD

Hc-NLP-63 (1) *MPIRRLPLLVTVILLSLCTSAAA*QHRLFYMLGNAVDGTSAAGLHVPRQDD

51 84

Ce-NLP-63 (39) LENMVNEIMRVQSVEQMNNHHVRRQIFSRKFW--

Na-NLP-63 (28) LQSIVSQLLTRVNDNNSEARHVRKQIFSRKFWKR

Hc-NLP-63 (51) LQSMVSQLLTRVNENNSDALHVRKQMFSRKFWKR

NLP-64

1 50

Ce-NLP-64 (1) -----------------------------*MMAQKTLIIAVMLVCSILQPM*

As-NLP-64 (1) --------------------------------------------------

Bm-NLP-64 (1) --------------------------------------------------

Di-NLP-64 (1) --------------------------------------------------

Na-NLP-64 (1) -------------------------*MSRSLCLFLALVSLTHS*FSLGNYYV

Hc-NLP-64 (1) -------------------------*MSRTLCLVLAFIAVTNA*FNLGNYYV

Bx-NLP-64 (1) *MQPALVICLVASVVGTAHA*FYALPPSFQRYMSDRSMLRPSAPLPQLVPFL

Gp-NLP-64 (1) ------*MRSTNRTIPFTGVLLLLLVLFMAKFCHC*DTNFRLSNFLPSYNRL

51 100

Ce-NLP-64 (22) *LA*LGSLTPSAAFRANMQQRERSPNTLFYMDG-ASKQYGDEIKDPIYKRFK

As-NLP-64 (1) ---------MEHVKQYEEPLQKYNSKVVDDNAGILKRATYLRQSPLKRLR

Bm-NLP-64 (1) ------------FDNIKLNFRKKDETIYKDKYGYLSMKDFGEQRPLKRLR

Di-NLP-64 (1) --------------------XRNDPTIYKDDYGSLMLNDSG----MKRLR

Na-NLP-64 (26) GALPYRFMDSPYENRIAFGQWRGGRDFPVG--DSALQKDKKDTLPMKRFK

Hc-NLP-64 (26) GSMPYRYLDAQYDGRLGFGPLRGGREFGIDNPTPELQKDKKDAAPMKRFK

Bx-NLP-64 (51) PPVPIALEEEPIAAEPHPNNAKGYEEVQVPR--RSLRQFIGETHQMKRMR

Gp-NLP-64 (45) AAINPDDDQQLPVDEDEAVVANAGRLFADDDDEQPKQMHTMNQQVVLPER

101 150

Ce-NLP-64 (71) PCYYSPIQCLIKRK------------------------------------

As-NLP-64 (42) PCFYSPIQCLMKRANVHGISANLAP-------------------------

Bm-NLP-64 (39) PCFYSPIQCLMKKRTARIENFMDNYR------------------------

Di-NLP-64 (27) PCMYNPIQCIVKRLKLRRR-------------------------------

Na-NLP-64 (74) PCYYSPIQCLIKRR------------------------------------

Hc-NLP-64 (76) PCYYSPIQCLIKRR------------------------------------

Bx-NLP-64 (99) PCFYSPIQCLMKKRSQP---------------------------------

Gp-NLP-64 (95) PCYFSPIQCLLRTPGQHEVFENHPKFNVFRADLQRPTIDQSVIGASRQHQ

151 197

Ce-NLP-64 (85) -----------------------------------------------

As-NLP-64 (67) -----------------------------------------------

Bm-NLP-64 (65) -----------------------------------------------

Di-NLP-64 (46) -----------------------------------------------

Na-NLP-64 (88) -----------------------------------------------

Hc-NLP-64 (90) -----------------------------------------------

Bx-NLP-64 (116) -----------------------------------------------

Gp-NLP-64 (145) QVRRHLLGLMNSKSLVVTPSSSARHKLQASEIGRQRLFGGNPWKLGL

NLP-66

1 50

Ce-NLP-66 (1) *MSSGKLFQFFIVFLATLLLADA*IPMVSSRDEDDQIIQKRLSNDALIRLLM

As-NLP-66 (1) ------MRPITSANFISNGRSPFAEMKFAEHDTRRLQRRSP--SIFPVLD

Na-NLP-66 (1) ----*MSFLKYIVVLSLSSFLIIHALA*MFNENEVPTIKKRLSNDALIRLIM

Hc-NLP-66 (1) -----*TMIYFAPVYFLLLLFIPTDNVA*MPEEDLLEITKRLSNDALIRLLI

51 98

Ce-NLP-66 (51) RNRGTQTQLGLKRGLVKKAEVERRSIDEDFSNCFLSPVQCMLPSSRK-

As-NLP-66 (43) R-------R------------GFDTGDADFQNCFLSPVQCMLPRMRRR

Na-NLP-66 (47) RN---QHSMFTSKRDEKRADFDRRSVDDDFSNCFLSPVQCMLPRSRR-

Hc-NLP-66 (46) R-----FAMQRKR-HDKRAEVDRRSVDDDFSNCFLSPVQCMLPRMKR-

NLP-67

1 50

Ce-NLP-67 (1) ---------------*MRVLTFLLVTLFALANVMQA*QRYDRAIYEALLNDL

Na-NLP-67 (1) ---------------*MKSVTLLFVLLLTVMHLSLS*ISHDRSIYEALLKNM

Hc-NLP-67 (1) ---------------*MKSLALLFVALLALVQVSIA*ISYDRLVYEALLKNM

Bx-NLP-67 (1) *MSLSSAMLTALRESRGVLMFSLLTVLLISTVTA*KPLSQDRRSLQILLRAI

51 100

Ce-NLP-67 (36) EREFVERELAQHVLEK-RELLRQDRQELDRVRRASEKKSYPRNCYFSPIQ

Na-NLP-67 (36) EQELAERELLRIIS---RRERAIVAESEPRERRSSEKKSYPRNCYFSPIQ

Hc-NLP-67 (36) EQEMAERELVRELS---RRVRAVPVEVEQRERRSSEKKSYPRNCYFSPIQ

Bx-NLP-67 (51) DNELESPYSDDLMPSRSRRAVASELPQDKRELLAYDKKSYPRICYFSPIQ

101

Ce-NLP-67 (85) CLFTRN----

Na-NLP-67 (83) CLFTRS----

Hc-NLP-67 (83) CLFTRS----

Bx-NLP-67 (101) CLFTRMSLKK

NLP-68

1 50

Ce-NLP-68 (1) -*MLLVLLFSLFSVGFG*MRVMRRELLKDQPTVLLLGPLEVLTSSSSE-DDT

Na-NLP-68 (1) -*MYLAVLLAFLSISCRA*SFVGHSPPNSPLTGRHALGESIEYNGSGESSKE

Hc-NLP-68 (1) *MYSVTVLSALLCISSG*ATFYGNS-LNSDFSGDQALYYVNEYPTSEEPQKR

51 100

Ce-NLP-68 (49) PDFPSLRDKRGVDPMSIPRLIKEPPLKKRSGDFKRGDVVYPSAAKQTVPL

Na-NLP-68 (50) PVLESIVTKRGVDPLSIPQLIREPPLKRG---DKRDSYVYPSTSSKLSDR

Hc-NLP-68 (50) LVREPPLIKRGIDPFSIPTLIKEPPLKRG---DKRDSYVYPSTNSQLSDR

101 150

Ce-NLP-68 (99) VRVREPPLKR--G----------QMFLEELLQFNDDAHRQFMDPLRRIRY

Na-NLP-68 (97) VPLREPPLKR-------------QEYFELSNLFSPSEKIVEWQDTTDENS

Hc-NLP-68 (97) IPLREPPLKRSQSLDDKVFVLVPEERHEPNGLANVYRRMLLLDQSTNHAA

151 172

Ce-NLP-68 (137) G-----------PNRLIYTW--

Na-NLP-68 (134) G-----EGKSNGKHVLLRLDQ-

Hc-NLP-68 (147) DSEAVPHPKLRSQRFLSRIWWH

NLP-69

1 50

Ce-NLP-69 (1) *MHFFPILLLSILLILISTCSS*TLVNNSPTAAFDTNAYSELNAKAKASMRR

As-NLP-69 (1) --------------------------------------------------

Na-NLP-69 (1) --------------------------------------------------

Hc-NLP-69.1 (1) ------------------------------------MCTVFARPSKNFEM

Hc-NLP-69.2 (1) -------------------------------------------MRQLEPL

Bx-NLP-69 (1) --------------------------------------------------

Gp-NLP-69 (1) --------------------------------------------------

51 100

Ce-NLP-69 (51) LADILDFEMYQRRLSAAPDNSYYYQISPPHHQRISSLPLTIFPLPDKRLH

As-NLP-69 (1) --------------------------------QQTLQPEDDR-PLVKRYN

Na-NLP-69 (1) -----------TLRLNHEIEREIYRRLSGGAKPRLRRKINSMEIPIKRIT

Hc-NLP-69.1 (15) FLPADYRQLGNTLQRVDDTDQEMFQGYYDRPMARLQRKVNTFALPIKRIP

Hc-NLP-69.2 (8) GTLFQDAPQCVDAPAGDVHDQEVFQGYYDRPMARLQRKVNTFALPIKRIP

Bx-NLP-69 (1) -----------------RGKQIHGTFEFSALSATVIQFQDDRDQRHKRYG

Gp-NLP-69 (1) --------------------------------------AGRSPTVNKRFN

101 112

Ce-NLP-69 (101) RIGGNIVMGK--

As-NLP-69 (18) RIGGMIVMGRRK

Na-NLP-69 (40) RIGGNIVMGRKK

Hc-NLP-69.1 (65) RIGGTIVMG---

Hc-NLP-69.2 (58) RIGGTIVMG---

Bx-NLP-69 (34) RIGGTIVMGR--

Gp-NLP-69 (13) RIGGNIMMGRK-

NLP-70

1 50

Ce-NLP-70 (1) --------------*MSVSRPLNLAILSILVGLLYLSACTC*APSVSAHHLG

As-NLP-70 (1) MLDDELRFLPRRMRQKPFADSSSGAITKRTASRRSISISVYVTIIISDQP

51 100

Ce-NLP-70 (37) LRLKKWYEWNN-DMEITKKWYDWQNVPHALQQ-----------KRQPFET

As-NLP-70 (51) LRPKKWYDWNDDETRLHKRWYDWQALPLSDSIDLMKKNRLSHLERQLHPG

101

Ce-NLP-70 (75) SDYIIE

As-NLP-70 (101) NDWLLG

NLP-71

1 55

Ce-NLP-71 (1) -------------------------------------------------------

As-NLP-71 (1) -------------------------------------------------------

Bm-NLP-71 (1) -------------------------------------------------------

Di-NLP-71 (1) -------------------------------------------------------

Na-NLP-71 (1) MNTEEGSSTDEKEIVDVLTHHAGEHEWMQPGRTDVETGRASNVDDRKERGRVPLW

Hc-NLP-71.1 (1) -------------------------------------------------------

Hc-NLP-71.2 (1) -------------------------------------------------------

Bx-NLP-71 (1) -------------------------------------------------------

56 110

Ce-NLP-71 (1) -------------------------------------------------------

As-NLP-71 (1) -------------------------------------------------------

Bm-NLP-71 (1) -------------------------------------------------------

Di-NLP-71 (1) -------------------------------------------------------

Na-NLP-71 (56) DGQLNATLRTKMNNLQTPECREQMNRGGGYITCRSSAKANAKDGKSGIMSDRNLT

Hc-NLP-71.1 (1) -------------------------------------------------------

Hc-NLP-71.2 (1) -------------------------------------------------------

Bx-NLP-71 (1) -------------------------------------------------------

111 165

Ce-NLP-71 (1) ----------*MKSSSVLSVALIVLVIVQLISASLA*SVPSSS---AVSDGQIDFDA

As-NLP-71 (1) -------MTSCCRLALVFAVIVFHSQWFTNATSVGWDYSKE-----NS-LQRQGG

Bm-NLP-71 (1) -----MAVFLGKIFEKSKIPFESVKLICSDVFCILKIFSRE----NLAMSQLFPW

Di-NLP-71 (1) ----------MLKTSKTTGIISSVSRTNSAAISLWDEESRNNAWGNLATSQLFPW

Na-NLP-71 (111) HNSEVLERLECEDVKRRESLYLFLMMSDVEDVTPHLHFRKI-----ASAKTEQGF

Hc-NLP-71.1 (1) ------------*MQTAIAYLLAFILFVQCLG*SSVGWAGERQ-----DDSEQVADP

Hc-NLP-71.2 (1) ------------*MQPTVTYLLALILFIRCFGPSVG*WEAER------KVDGQALDP

Bx-NLP-71 (1) -------------------MQNGTFLANGSGPPLLYGMVAQ-----ESQDPLAYF

166 217

Ce-NLP-71 (43) LAAKIEMLRPNRYWKR--AHNIDTRALNQFKNCYFSPIQCVLMERRRK----

As-NLP-71 (43) ISRPIASWEAALLEQKRAVHNVDARSISQFKNCYFSPIQCVLVEKRK-----

Bm-NLP-71 (47) HTISHEQTRHLEHQQQQPKHHIVLRSTNHFKNCYFSPIQCVLVEEQ------

Di-NLP-71 (46) RTISHKQTRHYEQRK--PEHHIVLRSANHFKNCYFSPIQCVLVEEQ------

Na-NLP-71 (161) GRNVLRQIGAYRLWKR--AHNIEPRALSQFKNCYFSPIQCVLMERRRR----

Hc-NLP-71.1 (39) RTAALRQMVPYRLWKR--AHIIEPRALNQFKNCYFSPIQCVLMERRRR----

Hc-NLP-71.2 (38) RTIALRELYPYRLWKR--SPYAGRRAAKRFKNCYFSPIQCVLMDRRRR----

Bx-NLP-71 (32) VQNPPNEVFNAAAKRN--IHRVQARAVNTFKNCYFSPVQCVLLERRRRSVQV

NLP-72

1 50

Ce-NLP-72 (1) -------------------------------------*MLTRVPVLILAVI*

Ts-NLP-72 (1) ------------------------------------------*MDLKIKIR*

Tm-NLP-72 (1) MALYDRIKPGDVFRDYNGELSNRQSEAASLNQSTVALGKRLRRIMSLKVV

As-NLP-72 (1) --------------------------------*MSISTQLFVSLCILTLMV*

Na-NLP-72 (1) ---------------------------------*MATSTMHSWLRFVLVCC*

Hc-NLP-72 (1) --------------------------------------*MQFWLRLTLVFC*

Bx-NLP-72 (1) ------------------------*MSLWAAKGSSRRAWTSWLTCFCLIIC*

Gp-NLP-72 (1) --------------------------------------MFFALFLSTAVQ

51 100

Ce-NLP-72 (14) *VMLALC*QEPEKPEKRP----ALLSRYGRA-VLPRYGKRSGN--LMESSQ-

Ts-NLP-72 (9) *IIFFLIGMALFSEA*KP----ASLARYGRA-ALPRYGKRAELISGLDAMR-

Tm-NLP-72 (51) AFVILLVSVLAEAKGP----LAMARYGRA-ALPRYGKRMEISNGPEYFR-

As-NLP-72 (19) *TAEFA*TNDILEPTKRA----ALLSRYGRA-VLSRYGKRSLPQSGYSSFG-

Na-NLP-72 (18) *FAVLIIA*SDQVPEKRP----ALLSRYGRA-VLSRYGKRSPLPKVAAPENE

Hc-NLP-72 (13) *FVVLLIA*DNQNLEKRP----AHLSRYGRA-LLSRYGKRSVISPNNGKYF-

Bx-NLP-72 (27) *YLMVMVTA*EFEKAEQPKRGNALLSRYGRA-VLSRYGKRSAPNFFESDVP-

Gp-NLP-72 (13) IRSHILPPQGANEAAG--GTILLSRYGRAAVLSRYGKRDGGAERREFNQS

101 150

Ce-NLP-72 (56) ------------NSLTEE-SSDVVCQLIDGKYICLPVDAVRFR---PFFL

Ts-NLP-72 (53) ------------DEVTEFKNSPCVYTGYEDLYRCSSMT------------

Tm-NLP-72 (95) ------------DDILEYRNNMCVYTGYEDLYRCSPVT------------

As-NLP-72 (63) ---EQTDVSPRIKTFALVLFEYCTERHIEGEYLRSCLNGVVFRRGEGVRK

Na-NLP-72 (63) TEIRQYRYRKENLSLYSSNHKTGEKKDFTELLLCRSVNGEFLCVPYTDEV

Hc-NLP-72 (57) ------------FLKVLSKRPSIKHRQTKSRSVVFHVHRIFFFNSSPVFY

Bx-NLP-72 (75) -------------FDGIAEEWLLCRRAPNDMLQCLPTPLYHY--------

Gp-NLP-72 (61) FGEGERAVHPPVAGPGPQCTHRLQGRARSAPTGCRAGPAVHPPVAGPGPQ

151 179

Ce-NLP-72 (90) -----------------------------

Ts-NLP-72 (79) -----------------------------

Tm-NLP-72 (121) -----------------------------

As-NLP-72 (110) GEKGGMNDRIGEREIDIYYAVAKCLLYHI

Na-NLP-72 (113) RK---------------------------

Hc-NLP-72 (95) -----------------------------

Bx-NLP-72 (104) -----------------------------

Gp-NLP-72 (111) CTHRLQGRPPPPDPK--------------

NLP-73

1 55

Ce-NLP-73 (1) *MSCSSSSMLFLVLIATTVLIAES*RVFYNRFDGGLSSDRFMEQKRDGAEASYDYDA

As-NLP-73 (1) -------*MLTKYARVLFMLVVALKFTSA*MITPSYTSIDYLNRRLQQANAYHEPII

Na-NLP-73 (1) --------*MKFCLALLALTVIAATIEA*RLFGYEDVLVDPTQDRDSSSQNEWG--Y

Hc-NLP-73 (1) -------*MNSTVSLCFVLVCFAAVIEA*RLFQLGDVLVDPSKDRDPTWIADW----

56 103

Ce-NLP-73 (56) NQVIRNTMKRNRQCLLNAGLSQGCDFSDLLHAQTQARKFMSFAGPGK-

As-NLP-73 (49) DDQSAPIMKRSRLCILNAGLSQGCDLSDMLFAKLQANKFSSFAGPGRR

Na-NLP-73 (46) KYSPARPSKRNRMCLINAGLSQGCDLSDILMAKQQASKFLSFAGPGRR

Hc-NLP-73 (45) EYALERPAKRNRMCLINAGLSQGCDLSDILMAKQQASKFMSFAGPGRR

NLP-74

1 50

Ce-NLP-74 (1) MIQTFFFLPNSNFSISFSLYFSQISVIFSPRVPGRFKSGRVAPSWCAGRR

Ts-NLP-74 (1) --------------------------------------------------

Tm-NLP-74 (1) --------------------------------------------------

As-NLP-74 (1) --------------------------------------------------

Bm-NLP-74 (1) --------------------------------------------------

Di-NLP-74 (1) --------------------------------------------------

Na-NLP-74 (1) --------------------------------------------------

Hc-NLP-74 (1) --------------------------------------------------

Bx-NLP-74 (1) --------------------------------------------------

Gp-NLP-74 (1) --------------------------------------------------

51 100

Ce-NLP-74 (51) EAESKTREICLSLGCAHSFYSTCASQPSEQAFPALPLPSSLNVNMNRFII

Ts-NLP-74 (1) -----------------------MRATCASVVTIRQHKREAYSIPSLRLL

Tm-NLP-74 (1) --------------------------------------------------

As-NLP-74 (1) --------------------------------------------------

Bm-NLP-74 (1) ------------------------------------------*MVSNISKS*

Di-NLP-74 (1) --------------------------------------------------

Na-NLP-74 (1) --------------------------------------------*MRSFFS*

Hc-NLP-74 (1) --------------------------------------------*MRSFFA*

Bx-NLP-74 (1) --------------------------------------------*MKVSGL*

Gp-NLP-74 (1) ------------------------------------------MSSGLSPA

101 150

Ce-NLP-74 (101) SMIALLAVFCAVSTASPLLYRAPQYQMY----------------------

Ts-NLP-74 (28) FSTIFVYLFLKAVEKYPIRLQIMNGKLIFGIIFLYATIFTYITAVPVVYE

Tm-NLP-74 (1) --------------------------------------------------

As-NLP-74 (1) -------MINAITIRESRLDNFG---------------------------

Bm-NLP-74 (9) *TLRTFLLLILTVTIAST*FEFGELMYGDN----------------------

Di-NLP-74 (1) --------------------------------------------------

Na-NLP-74 (7) *VILALVALVFAVASA*NPLLYRVP--QMY----------------------

Hc-NLP-74 (7) *IFLTLLVSVVVISTA*NPLLYRAP--QMY----------------------

Bx-NLP-74 (7) *VQALFVLLVIALAQA*QPLVYRPYGLHNVG------------------IPV

Gp-NLP-74 (9) QLTTFGEDNVNIGFSTANNSSANGTPTYTACTS------------FDEPH

151 200

Ce-NLP-74 (129) DDVQFVKRSNAELINGLIGMDLGKLSAVGKRS-------NAELINGLLSM

Ts-NLP-74 (78) ADLTAQEKEILQSLAEKIGRTYGSRFDSGRTIKR----ANAELVNGLLGM

Tm-NLP-74 (1) ----------LEALLERAGRRYLTRMASGNYIKR----ANAELVNGLLGM

As-NLP-74 (17) DGELLTKRSNAELINGLIGMDLNKLSAIGKRS-------NAELINGLLGM

Bm-NLP-74 (37) ELQGAWKRSNAELINGLIGMDLGKLAKAGKRSRTSSMITNDENINNDIRF

Di-NLP-74 (1) EMRGVWKRSNAELINGLIGMDLGKLTRAGKRS-VSPLITNDDNTNSN---

Na-NLP-74 (33) DDTQFVKRSNAELINGLIGMDLGKLSAVGKRS-------NAELINGLLGM

Hc-NLP-74 (33) DDVQFVKRSNAELINGLIGMDLGKLSAVGKRS-------NAELINGLLGM

Bx-NLP-74 (39) STYRHAKRSNAELINGLIGMDLNTLQAVGKRS-------NAELINGLIGM

Gp-NLP-74 (47) EFQAVEKRRNAEFINGLLTMERLNAIGKRSPP-----VPDVDDVQQLFWR

201 214

Ce-NLP-74 (172) NLNKLSGAGRR---

Ts-NLP-74 (124) RYGDLMRAG-----

Tm-NLP-74 (37) RYGDLMRAG-----

As-NLP-74 (60) NLNRLSSAGRR---

Bm-NLP-74 (87) KMNLKKLLSNQRHY

Di-NLP-74 (47) --------------

Na-NLP-74 (76) NLNKLSSAGRR---

Hc-NLP-74 (76) NLNKLSSAGRR---

Bx-NLP-74 (82) DLNRLSSIGRRR--

Gp-NLP-74 (92) PQHPFFWTE-----

NLP-75

1 60

Ce-NLP-75 (1) ------------------------*MGSSPILLVLAISIGLASA*CFLNSCPYRRYGRTIR-

As-NLP-75 (1) ------------------------------CAGRRIRTAILIDFFINQCPYRHYGRTQCA

Na-NLP-75 (1) ----------------------------*QSLAFVIFSLSIVNG*CFLNSCPYRRYGRTLR-

Hc-NLP-75 (1) SVKMDYPLRAAGGSFRRGRDELFNIMNWLCFAFLIVSTIIIDGCFLNSCPYRRYGRTIR-

Bx-NLP-75 (1) ----------------------*MGDRTRTLALIICLLTMHCDG*CFLNSCPYRRIGRSNQP

61 120

Ce-NLP-75 (36) -----------------CSSCGIENEGVCISEGRCCTNEECFMSTECSYSAVCPELFCKI

As-NLP-75 (31) ------------------------------------------------------------

Na-NLP-75 (32) -----------------CALCAPSREGICHGDGQCCTHTHCFPSKDCGTRDSCPENFCRF

Hc-NLP-75 (60) -----------------CESCGEFMNFHR-------------------------------

Bx-NLP-75 (39) ANAVIPSGPSVDITVETCGICGPKNNGICVNERVCCTVEECKEDTGCQNVHACPLPLCII

121 155

Ce-NLP-75 (79) GHHPGYCMKKGYCCTQGGCQTSAMC----------

As-NLP-75 (31) -----------------------------------

Na-NLP-75 (75) DSHSGVCITKLLCCTSSHCIRSMQCA---------

Hc-NLP-75 (72) -----------------------------------

Bx-NLP-75 (99) DNGPGFCATNTLCCADKFCQRNLQCMVVASKQLNL

NLP-76

1 50

Ce-NLP-76 (1) --------------------------------------------------

As-NLP-76 (1) -----------------------------------------------*MRY*

Bm-NLP-76.1 (1) --------------------------------------------------

Bm-NLP-76.2 (1) --------------------------------------------------

Di-NLP-76.1 (1) --------------------------------------------------

Di-NLP-76.2 (1) --------------------------------------------------

Na-NLP-76.1 (1) --------------------------------------------------

Na-NLP-76.2 (1) MDIRGGDCFNSQGKAVEKRLEGGLAITVRLILFEAPVKTCIENLAWVGYF

Hc-NLP-76 (1) --------------------------------------------------

Bx-NLP-76 (1) --------------------------------------------------

Gp-NLP-76 (1) -------------------------------------------------*F*

51 100

Ce-NLP-76 (1) --*MSRKFLLVFIAICVLSQNAMA*LRGALFRSGRSLIHR-------QSQNE

As-NLP-76 (4) *NRSMNAALLCSIVLLITLPTADA*LRGALFRSGRSMQRISRQSTDAKFPLD

Bm-NLP-76.1 (1) ----*MKFSLSFILFFLMITTSMT*LRGALFRSRKAALES-------IIRAN

Bm-NLP-76.2 (1) -MSATYGITCVFVGLALFTATNALRGALFRTGKSFWSSG------KYDPY

Di-NLP-76.1 (1) ------------VFFLLITTSMALRGALFRSRRASLDS-------IIRAH

Di-NLP-76.2 (1) -------YSGVFICLALFTATNALRGALFRTGKRLWSSD------EYDPY

Na-NLP-76.1 (1) ----MRAFIYVLAVLATTSSVFGIRGALYRTGRSATPAYG---KQIPMIK

Na-NLP-76.2 (51) QSFFKAHTIEALYMVLLTTSVLSLRGALYRSGRSFRFRQPTVVEHNALVE

Hc-NLP-76 (1) ---*MLRSLLFLVVLTAIPQTAHG*VRGALFRTGRADLVPF------FDNVN

Bx-NLP-76 (1) -----*MFPKSFLVILLMPLMVEC*IRGALFRSGRSPGLQKTRGSQAIKMLY

Gp-NLP-76 (2) *KQKDVLLLLFVLLCCSAPPSAMA*IRGALFRSGRSMAIPLWR-PPAKNTGA

101 141

Ce-NLP-76 (42) EAVLNQPHLLPAEKRSPIVELYPVVDSGNVEPEAFPAYFRF

As-NLP-76 (54) SSAFEFQHTMPFYPFGYRKRFDVDQELHDFRNE--------

Bm-NLP-76.1 (40) TGRQDFLRFGRTNEKYNDRHLIASALLTPVDLK--------

Bm-NLP-76.2 (44) TNLFELGKSLKAIDLMDNGNRNVISTNRDAKKTIYLQ----

Di-NLP-76.1 (32) ASRVRYLHKFKNLSLFE------------------------

Di-NLP-76.2 (38) MSLFKPSRSVEMVSLI-------------------------

Na-NLP-76.1 (44) NPQVEMPYGSQPVMVG-------------------------

Na-NLP-76.2 (101) TKRNVVVAYPPNSMESLDNQEANLRVFPNFLL---------

Hc-NLP-76 (42) FISIK------------------------------------

Bx-NLP-76 (46) SNLFRPDNGPLLRPQISYNNVIDNEQIFSRLRTLLMPFNAE

Gp-NLP-76 (51) ASGYSAGLASTSEKLANYARPSVIVDLRQVVQVLVADSKS-

NLP-77

1 50

Ce-NLP-77 (1) -----------------------------------------*MCRLAVFAL*

As-NLP-77 (1) ----------------------------------------------*MKVL*

Bm-NLP-77 (1) ------------------------------------------*MKMMKFVL*

Di-NLP-77 (1) --------------------------------------------------

Na-NLP-77 (1) ---------------------------------------------*-MLKL*

Hc-NLP-77 (1) --------------------------------------------------

Bx-NLP-77 (1) ---------------MWNVPCGHDRRETVNPEAPLKPQATIMSSLSCVLL

Gp-NLP-77 (1) MSEGVRTLKFRPSRRKHNNFSSTFTLDHSIPDMTSSSALLLVASLAMTVL

51 100

Ce-NLP-77 (10) *LAVAAVSVYG*QPAGGQD---VPPFLRNATPAQLQSFQQLIQANGHLTETA

As-NLP-77 (5) *IIFVAIVVIAFA*QGPQG---PPPFLVGAPANVVAEFKQIITGAPDKTDAE

Bm-NLP-77 (9) *LAFPVLLLVCPVFG*EEQQFMIPPFLLGAPESAVREMKELFQKYANKPDSQ

Di-NLP-77 (1) ----------MILEQLE---VPPFLVGAPQSVIKQFYDLLKADETKTDAQ

Na-NLP-77 (5) *VALVCLVAICFA*QGPQG---PPPFLQSAPAAVQQDFDKLFVNAGSKTDAE

Hc-NLP-77 (1) ---------------------------------------MANAGSLTDAQ

Bx-NLP-77 (36) VALFGLLALSQAQNIP----PPPFLAGESPDVIQSFQRVLERGNDLTDAQ

Gp-NLP-77 (51) ALCRAQLSAPSSQGAGQ---APPFLAEAGADKINEFYQFVASLGHLKDAE

101 150

Ce-NLP-77 (57) LDGKVQAWVNQQGGKVAADWADFQKFIKGQQGQAEAAHQAAVSNFSPAAK

As-NLP-77 (52) IDRDIENWVSRQGPKIKTEFNKFKTQMQQGKARAEAAHRASIAKFSPAAK

Bm-NLP-77 (59) LEAAVEEWVNGKGGMIKEKYNQFKANMQLMHEKTALLRKAMAENLSPEAK

Di-NLP-77 (38) TEADVEAFINRLGGTYKTRFDQFKQEIKQGKAAYERLHQQAVAKFSKEAR

Na-NLP-77 (52) IDKMVQDWVGKQDASIKTAFDAFVKEVKAAQAQGEAAHQAAIAKFSAEAK

Hc-NLP-77 (12) IDQKVQDWVNQQDPAIKTAFDAFVQQVKQAQAQSEAAHQAAIANFSPAAK

Bx-NLP-77 (82) LDREVDAWVQTQSAAVKNKYAQFKSDLAKHQAQAEAAHKAAVSRFSSVAR

Gp-NLP-77 (98) IDAKVKEWVGKQSDDVQKKYAGFEQEREKAKAQGEAAHEEAIKHFSEGAK

151 198

Ce-NLP-77 (107) KADADLTAISNDSSLSVQAKGQKIQAYLNSLPANVKAELEKAQGQ---

As-NLP-77 (102) AADAQLTAIADNPNLKGREKQQKITSLLQSLPAAVQAELQKEMQG---

Bm-NLP-77 (109) KADVDLSSIGKDKTLSEQQKREKFKEYLRNLSPAVRNELQAVFYAKF-

Di-NLP-77 (88) EADAKMSAIADSPSLTTQQKTQQIQAIMD-------------------

Na-NLP-77 (102) AADAKLSAIANDRSKTNAQKGAEIDSVLKGLPPNVRTEIENAMKG---

Hc-NLP-77 (62) EADAKLSAIAADPNKTNQQKAQEIQATLASLSPEVRNEIETAMKGGK-

Bx-NLP-77 (132) EADAKLSTIANNPSLTSAQKNAQIEKFVSQLPPAVRKEIESAMQG---

Gp-NLP-77 (148) KADKELSEIANNVNLSGQDKAKQINDKVQSLPKDVRDEIEGNVGGGRN

NLP-79

1 50

Ce-NLP-79 (1) --------------------*MSTRWFVFVALMALVLLSAHTTEA*M-----

As-NLP-79 (1) MKRQVAEIARMDRTTYAKRALATSLFVMCALCWLANATPLIDGNLRKQST

Na-NLP-79.1 (1) ---------MNLRSFVSFRMQSSTRWSFLAIISLLLVVQSFANQG-----

Hc-NLP-79.1 (1) -----------*MDSLQVMMHDNKRTLLLVIVGLLLLVEQSYA*SLR-----

Na-NLP-79.2 (1) ------------------MRYSVCWVLLAAFTIMSMTSARIHER------

Hc-NLP-79.2 (1) ------------------*MRYSICWVVLATACSLIFLRITQA*ELS-----

51 100

Ce-NLP-79 (26) ------PRPGQMSLSNTRNCFFSPMGCVFVPKMSRIRKLSADFRNEIPPP

As-NLP-79 (51) LSFVGNGMQQYENSRYDRNCFFSPMNCVFYEHPMDAPVRRFTFGEEALRR

Na-NLP-79.1 (37) ------EIDRLLGARVERNCFFSPMGCMFLPGKKLSRVRRLPELADQDPQ

Hc-NLP-79.1 (35) ------ELDRVLGARIERNCFFSPMGCMFLPNKKFARVRRLQESEDES--

Na-NLP-79.2 (27) ------YADYAITLRSSRNCFFSPIGCVFFSSGVEFPRARHKKRHVNRRH

Hc-NLP-79.2 (28) ------ALDYRIAARADRNCFFSPIGCVFLTGNKQIRARRHKNVDYPWSN

101

Ce-NLP-79 (70) DYI------

As-NLP-79 (101) ---------

Na-NLP-79.1 (81) SERSLLWFI

Hc-NLP-79.1 (77) ---SPLQFL

Na-NLP-79.2 (71) ---------

Hc-NLP-79.2 (72) ---------

NLP-81

1 50

Ce-NLP-81 (1) ------------------------*-MLKSSIFSLLIVLLMVCS*-------

As-NLP-81 (1) ----------------------*MCRPAQTAACALLLFLSLFV*S-------

Bm-NLP-81 (1) ------------------*MIYGTFLLLLHLPLLFAIAILNLHC*-------

Di-NLP-81 (1) -------------------------XXXXXXWIFFLILLFFFL-------

Na-NLP-81 (1) -----------------------------*MHAILGLLLAMVAA*-------

Hc-NLP-81 (1) XDETHECLGILFVAIQLWSISAHRSSTWKMHVILRLLLAMVAA-------

Bx-NLP-81 (1) -----------------------*MSPLSTVILALFMLLVVQSG*-------

Gp-NLP-81 (1) -------MNISKSQNLSSSSMNYVPMFLRFVLLIACFLLLMSGPFAMAND

51 100

Ce-NLP-81 (19) -----*FGNVSA*NDFFLRSAKWVSKMNPSGGALVSGRGGFRPGFVS--RDW

As-NLP-81 (22) -----*STQT*KPSDMFIRSPKWARLA-PSGGSLVSGRGNFRPGFVSSAADT

Bm-NLP-81 (26) -----*QAVDA*TNDYFFRSPKWVNLG-PSGGSLVSGRGNFRPGFSSSVSDS

Di-NLP-81 (19) -----QIVDAENEYFAQPPKTVYLR-PSGGLLVSGRGNFRPGFLPSNIDN

Na-NLP-81 (15) -----VAAFSNSDFFIRSAKWIPSG---GAGLVSGRGNFRPGFTG--RDW

Hc-NLP-81 (44) -----AAAFTNGDFFVRSAKWIPSG---GAGLVSGRGNFRPGFTG--RDW

Bx-NLP-81 (21) -----*KA*DDMDSYYFRRNAKWISLAP-SGGSLVSGRGNFRPGFYGR-GGS

Gp-NLP-81 (44) DVANELAMRGTQQFFRRNAKWTNLP-SGGGSLVSGRGNFRPGFHSS-LRY

101 143

Ce-NLP-81 (62) RHAMAEPNFVKRSYNDY--------------------------

As-NLP-81 (66) RMLLSEPLLIFKKQVLFQAKHISALSTVYQLVSSISADNRYVK

Bm-NLP-81 (70) RFYLSEPLFAFKRT-----------------------------

Di-NLP-81 (63) PFYFFEPLFALRV------------------------------

Na-NLP-81 (55) RSAMAEPNFNKRSAQPWFSEDY---------------------

Hc-NLP-81 (84) RSAIAEPNFRKRSVESWYSDEY---------------------

Bx-NLP-81 (64) AAYMGDPVLFKRSQPADLE------------------------

Gp-NLP-81 (92) LAQLSEPSFAFKRSPQHFPMTMDELELADNFLGTQ--------

NLP-82

1 60

Ce-NLP-82 (1) -------------------------*MPSYHTVIIILLISIISTTS*FILQDLPLERFERSG

Na-NLP-82 (1) ------------------------FQMGFVRSVLVWCAIVCLTLSFVLQDLPLERYERS-

Hc-NLP-82 (1) MGGSFGPIIPLTTSYVRSLPYEVLSGMSFVRSLLLWCAVACLTLGFVLQDLPLERFERG-

61 120

Ce-NLP-82 (36) HGSDELLGGGSQFDRHIRSGLSYRPMPNLMARYRMMSG----------------------

Na-NLP-82 (36) -------QREAYFERPYRALPGPAFIYNRFM-----------------------------

Hc-NLP-82 (60) -------YPEISFDRASQAECVDSELVSIVIIIVVIVMFITGLQAVVRIGNSLHSITVVL

121

Ce-NLP-82 (74) ---

Na-NLP-82 (60) ---

Hc-NLP-82 (113) FFE

NLP-83

1 60

Ce-NLP-83 (1) ----------------*MARFTPLLMILLALVPLYYS*LDCRKFSFAPACRGIMLKRSGGHP

Ts-NLP-83 (1) --------------*MCTSVLPFITIIVVSIQLSSC*NINCRNYPFAPKCRGIILKKSVLS-

Tm-NLP-83 (1) MQFSVVPQTTTAYLTAHRSIVVIFCLSSSLTAYYAFEFFRKYPYAPPCRGIVLKRSPIYE

As-NLP-83 (1) -------------------*MRIVIFALFALVPLVSA*LDCRKFSFAPACRGIMLKRSEGS-

Na-NLP-83 (1) -------------------*MRIILFVFLSLIAIALS*LDCRKFSFAPACRGIMLKRSETTE

Hc-NLP-83 (1) -------------------*MRSLLFLIVAIIPLAIA*LDCRKFSFAPACRGIMLKRSEVAE

61 120

Ce-NLP-83 (45) MIAEQQPMIDNAKAREVQMMELLIRNIEDEALIANSDCVSMSWLRDRLVNAKNEMPQ---

Ts-NLP-83 (46) -------------------LKANREIVEEDENPIIK------------------------

Tm-NLP-83 (61) P--------------LP--KTHMTHIATLK------------------------------

As-NLP-83 (41) ---------AVAFDTDMLPLIAVCWHFQELKLQKVRSRTLLFGK----------------

Na-NLP-83 (42) RQQ------SLELYQLAQNLEMLSLLIREAEAEGIKECIPISWIRNKLATKASDAPPPPL

Hc-NLP-83 (42) VES------PLDLDQLAQNLETLSWILRLAEADGIKDCVPLSWIKHKLTSSSWLTSRS--

121 142

Ce-NLP-83 (102) ----------------------

Ts-NLP-83 (63) ----------------------

Tm-NLP-83 (75) ----------------------

As-NLP-83 (76) ----------------------

Na-NLP-83 (96) FTVSRDSRDRNEAHAQNIRTHK

Hc-NLP-83 (94) ----------------------

NLP-85

1 50

Bm-NLP-85 (1) *MSSFRLSTIYFCRTLLGTLICCYLLTDHVTA*ITDDSNIAKIPAYRTFQET

Di-NLP-85 (1) *MPSFRPWINHFHKTLLSTLICYYLLADHATG*INDGSNFMTYPAYR--QD-

51 100

Bm-NLP-85 (51) GYGLNEASHLRSLRSALGMDNIG---QITYPLTSRSAAMISGRGFRPGKR

Di-NLP-85 (48) LYGMNDASHLRSLRSDLGMNNIGHVEDFNYPLASRSAAMISGRGFRPGKR

101

Bm-NLP-85 (98) YDAEVISFM

Di-NLP-85 (98) YDAGIISFI

NLP-85 Pan-phylum

Note: additional species *Acanthocheilonema viteae, Brugia pahangi, Brugia timori, Ditylenchus destructor, Elaeophora elaphi, Enterobius vermicularis, Gongylonema pulchrum, Loa loa, Onchocerca flexuosa, Onchocerca volvulus, Onchocerca ochengi, Syphacia muris, Thelazia callipaeda, Wuchereria bancrofti.*

1 50

A. viteae NLP-85 (1) --------------------------------------------------

B. malayi NLP-85 (1) --------------------------------------------------

B. pahangi NLP-85 (1) --------------------------------------------------

B. timori NLP-85 (1) --------------------------------------------------

D. destructor NLP-85 (1) MSGLHLPLLNSKPKMTSKTILYLMFGVFGMCLCQEGSAFEPTRTLSIATG

D. immitis NLP-85 (1) --------------------------------------------------

E. elaphi NLP-85 (1) --------------------------------------------------

E. vermi NLP-85 (1) --------------------------------------------------

G. pulchrum NLP-85 (1) --------------------------------------------------

L. loa NLP-85 (1) --------------------------------------------------

O. flexuosa NLP-85 (1) --------------------------------------------------

O. volvulus NLP-85 (1) --------------------------------------------------

O. ochengi NLP-85 (1) --------------------------------------------------

S. muris NLP-85 (1) --------------------------------------------------

T. callipaeda NLP-85 (1) --------------------------------------------------

W. bancrofti NLP-85 (1) --------------------------------------------------

51 100

A. viteae NLP-85 (1) --------------------------------------------------

B. malayi NLP-85 (1) ----------------------------------------------MSSF

B. pahangi NLP-85 (1) ----------------------------------------------MSSF

B. timori NLP-85 (1) --------------------------------------------------

D. destructor NLP-85 (51) RGNIFRPSYAIGIPRFFYNGFQPYQFMNKRSMSSAVPAKFMSLAGEDSEL

D. immitis NLP-85 (1) ----------------------------------------------MPSF

E. elaphi NLP-85 (1) ----------------------------MNVQFAKKKFSATAKFQVMPSL

E. vermi NLP-85 (1) --------------------------------------------------

G. pulchrum NLP-85 (1) --------------------------------------------------

L. loa NLP-85 (1) ----------------------------------------------MSPS

O. flexuosa NLP-85 (1) ----------------------------------------------MSPF

O. volvulus NLP-85 (1) ----------------------------------------------MSSF

O. ochengi NLP-85 (1) ----------------------------------------------MSSF

S. muris NLP-85 (1) --------------------------------------------------

T. callipaeda NLP-85 (1) ------------------------------------------------MV

W. bancrofti NLP-85 (1) ----------------------------------------------MSSF

101 150

A. viteae NLP-85 (1) --------------------------------------------------

B. malayi NLP-85 (5) RLSTIYFCRTLLGTLICCYLLTDHVTAITDDSNIAKIPAYRTFQETGYGL

B. pahangi NLP-85 (5) RLSTIYFCRTLLGTLICCYMLTDHVTAITDDSNFAKIPAYRTFQETGYGL

B. timori NLP-85 (1) --------------------------------------------------

D. destructor NLP-85 (101) DNDLQFMKRSAALGRTRFRPGKRSLVTMEELEDMGMLPGFSLLTEAQMGK

D. immitis NLP-85 (5) RPWINHFHKTLLSTLICYYLLADHATGINDGSNFMTYP-AY---RQDLYG

E. elaphi NLP-85 (23) RPSIIHFHKTLLMLLSCS-LLADHATGILDNSNYGRLPSSF---RLGRFG

E. vermi NLP-85 (1) --------------------------------------------------

G. pulchrum NLP-85 (1) --------------------------------------------------

L. loa NLP-85 (5) RSSSIHFNRTLLGTLICCYLSADHVTGIIDDSNTAKFPVYR---QTGCGS

O. flexuosa NLP-85 (5) RPWIIHFHRNLLATLICCYLLTSHVTGVFDDSSFK------------A--

O. volvulus NLP-85 (5) RLWIIHYHRNLLGTLIYCYLLASSVTGIIDDSNFIRYP-AY---KKTAHD

O. ochengi NLP-85 (5) RPWIIHFHRNLLGTLIYCYLLASSVTGIIDDSNFIRYP-AY---KKTAHD

S. muris NLP-85 (1) --------------------------------------------------

T. callipaeda NLP-85 (3) RSQLLHWQ---IIYLICSCFWTDRVIANLYYPNFSRLSENKVDNERTPCG

W. bancrofti NLP-85 (5) RSSTIYFCKTLLGTLICCYFLTDHVTAITDDSNFAKFPAYRTFQETGYGL

151 200

A. viteae NLP-85 (1) --------------------------YPLASRSAVMISGRGFRPGKRYDE

B. malayi NLP-85 (55) NEAS-HLRSLRSALGMDNIG---QITYPLTSRSAAMISGRGFRPGKRYDA

B. pahangi NLP-85 (55) NEAS-HLRSLRSALGMDNIG---QITYPLTSRSAAMISGRGFRPGKRYDA

B. timori NLP-85 (1) --------------------------YPLTSRSAAMISGRGFRP------

D. destructor NLP-85 (151) RSMAVGRVGFRPGKRSIATGRAGALFWNPNYAKAAVISQRGFRPGKRSLT

D. immitis NLP-85 (51) MNDASHLRSLRSDLGMNNIGHVEDFNYPLASRSAAMISGRGFRPGKRYDA

E. elaphi NLP-85 (69) LNEVAHLRSLRSALGMENIGHVESFTYPLASRSAAMISGRGFRPGKRYDT

E. vermi NLP-85 (1) -----------------------SFQIPILSRSATMISGRGFRPGKRAQS

G. pulchrum NLP-85 (1) -----------------------KFQYPLNSRSAAMISGRGFRPGKRSDL

L. loa NLP-85 (52) NEGSSHLRSLRSVFGMDSVG---QVVYPLTSRSAAMISGRGFRPGKRYDT

O. flexuosa NLP-85 (41) IHELEFLRSLRSALERKSNGPLKNFNYPIKSRSAAMISGRGFRPGKRYDA

O. volvulus NLP-85 (51) LNKASFLRSLRSVLGIDGTGYIEGFTYPLTSRSAAMISGRGFRPGKRYDA

O. ochengi NLP-85 (51) LNKASFLRSLRSVLGIDGTGYIEGFTYPLTSRSAAMISGRGFRPGKRYDA

S. muris NLP-85 (1) ------------------KRSLEYQEIPILSRSATMISGRGFRPGKR---

T. callipaeda NLP-85 (50) TNEVPYFRSIRSPILINNEDY-DSFAYPLSSRSAAMISGRGFRPGKRFNQ

W. bancrofti NLP-85 (55) NEAS-HLRSLRSALGMDNIG---QITYPLTSRSAAMISGRGFRPGKRYDA

201 240

A. viteae NLP-85 (25) GFISFM----------------------------------

B. malayi NLP-85 (101) EVISFM----------------------------------

B. pahangi NLP-85 (101) EVISFM----------------------------------

B. timori NLP-85 (19) ----------------------------------------

D. destructor NLP-85 (201) LSENADSWEQMNMVERLTDN--------------------

D. immitis NLP-85 (101) GIISFI----------------------------------

E. elaphi NLP-85 (119) GIISFM----------------------------------

E. vermi NLP-85 (28) TVSYPIVLYRINVSPLSFPFFSLFSSSTTFCIIDIQIYPV

G. pulchrum NLP-85 (28) RNIAFM----------------------------------

L. loa NLP-85 (99) EIISFM----------------------------------

O. flexuosa NLP-85 (91) PILSFM----------------------------------

O. volvulus NLP-85 (101) EIISFM----------------------------------

O. ochengi NLP-85 (101) EIISFM----------------------------------

S. muris NLP-85 (30) ----------------------------------------

T. callipaeda NLP-85 (99) NTAMM-----------------------------------

W. bancrofti NLP-85 (101) EIIPFM----------------------------------

NLP-86

1 50

As-NLP-86 (1) MLDCVESAFVGGACCLYTNQRYSIPQQHSFSCIRCRDALVACGALRFDYR

Bx-NLP-86 (1) -----*MRLLLALLFTLLAVGFA*DFLEPNELDQMEDIDVPRERRSSLASGR

Gp-NLP-86 (1) ---*MTSLPLLTLLSVLLALQLIGQLFA*DKFEQVEDQDQLNQQQLTIGSNR

51 100

As-NLP-86 (51) GRCVSFSIRSLASGRWGLRPGKRS-QDVPIYTDELGLDGNSPLYDALRQS

Bx-NLP-86 (46) WGLRPGKRSSLASGRWGLRPGKRSIVDLVLEDDIEDESDKTRERRNLASG

Gp-NLP-86 (48) DIR------SLANGRWQLRPGKR--ASLIDFRPMDGLEERARRFRNLYAL

101 144

As-NLP-86 (100) HFVYVVRK------------------------------------

Bx-NLP-86 (96) RWGLRPGKRSLANGRWGLRPGKRSMGMMPTQARHNPIMLLIPQL

Gp-NLP-86 (90) LPPLNNWN------------------------------------

NLP-86 Pan-phylum

Note: additional species *Ascaris lumbricoides, Globodera rostochiensis, Meloidogyne arenaria, Meloidogyne incognita, Meloidogyne javanica, Panagrellus redivivus, Parascaris univalens, Syphacia muris, Toxocara canis*.

1 50

A. lumbricoides NLP-86 (1) --------------------------------------------------

A. suum NLP-86 (1) --------------------------------------------------

B. xylophilus NLP-86 (1) --------------------------------------------------

G. pallida NLP-86 (1) --------------------------------------------------

G.rostochiensis NLP-86 (1) --------------------------------------------------

M. arenaria NLP-86 (1) --------------------------------------------------

M. floridensis NLP-86 (1) --------------------------------------------------

M. hapla NLP-86 (1) --------------------------------------------------

M. incognita NLP-86 (1) --------------------------------------------------

M. javanica NLP-86 (1) --------------------------------------------------

P. redivivus NLP-86 (1) MNKCNGASEPVGAHQDRFSSRRSKKPKAVYPQTVTSTKHFITLKNNGLHH

P. univalens NLP-86 (1) --------------------------------------------------

S. muris NLP-86 (1) --------------------------------------------------

T. canis NLP-86 (1) --------------------------------------------------

51 100

A. lumbricoides NLP-86 (1) --------------------------------------------------

A. suum NLP-86 (1) -------------------------------------------MLDCVES

B. xylophilus NLP-86 (1) --------------------------------------------------

G. pallida NLP-86 (1) --------------------------------------------------

G.rostochiensis NLP-86 (1) --------------------------------------------------

M. arenaria NLP-86 (1) --------------------------------------------------

M. floridensis NLP-86 (1) --------------------------------------------------

M. hapla NLP-86 (1) --------------------------------------------------

M. incognita NLP-86 (1) --------------------------------------------------

M. javanica NLP-86 (1) --------------------------------------------------

P. redivivus NLP-86 (51) PGGQLSLTTLLAKIENQRLGKSTSIASQNEVNKNPATSKNLTFEDSPTQL

P. univalens NLP-86 (1) -------------------------------------LKVRLWEEHVASA

S. muris NLP-86 (1) --------------------------------------------------

T. canis NLP-86 (1) --------------------------------------------------

101 150

A. lumbricoides NLP-86 (1) -------------------MRSLLAVLFVSIIVDVVYPSPLYVAGPSISK

A. suum NLP-86 (8) AFVGGACCLYTNQRYSIPQQHSFSCIRCRDALVACGALRFDYRGRCVSFS

B. xylophilus NLP-86 (1) ----MRLLLALLFTLLAVGFADFLEPNELDQMEDIDVPRERRSSLASGRW

G. pallida NLP-86 (1) --MTSLPLLTLLSVLLALQLIGQLFADKFEQVEDQDQLNQQQLTIGSNRD

G.rostochiensis NLP-86 (1) --MTSLPLLTLFSVLLALQLIGQSFADEFEQVEDQDLLNPQQLTIDSNRN

M. arenaria NLP-86 (1) --------MRFSIALIVFAFILVIFN--CAESKDEGGNQEEIEQQYQNRR

M. floridensis NLP-86 (1) --------MRFSIALTIFAFILVIFN--CAESKDEGGNQEEIEQQYQNRR

M. hapla NLP-86 (1) --------MRFSIAFLVLTFILVISTMFVVESRDEGGNQEEIEQQYQNRK

M. incognita NLP-86 (1) --------MRFSIALIVFAFILVIFN--CAESKDEGGNQEEIEQQYQNRR

M. javanica NLP-86 (1) --------MRFSIALTIFAFILVIFN--CAESKDEGGNQEEIEQQYQNRR

P. redivivus NLP-86 (101) INMVAARSAQTSSAFNKMLVAMVLVTILAAANAHPFVYGSDIQAPASFNI

P. univalens NLP-86 (14) RISGIQFQSSTHSVAYGAEMRSWLAVLVVSIIVDVVYPSPLYVAGPSISK

S. muris NLP-86 (1) -----MRLLLTLLLVSSFCVLISAIPLLDGLKEDSGYVDKR---------

T. canis NLP-86 (1) -MDPAMLFLESLCEYDAAKRAVSAVNESIENVEFHAHFYCAFADYLKELF

151 200

A. lumbricoides NLP-86 (32) VS----------EFASGRWGLRPGKRSQDVPIYTDELGLDGN---SPLYD

A. suum NLP-86 (58) IR----------SLASGRWGLRPGKRSQDVPIYTDELGLDGN---SPLYD

B. xylophilus NLP-86 (47) GLRPGKRS----SLASGRWGLRPGKRS--IVDLVLEDDIEDE---SDKTR

C. sinica NLP-86 (148) VENDNMNNNQKRSLANGRSQWRPGKKS----SINSRVNLGNGMDLIVDAN

G. pallida NLP-86 (49) IR----------SLANGRWQLRPGKR----ASLIDFRPMDGL---EERAR

G.rostochiensis NLP-86 (49) IR----------SLANGRWQLRPGKR----ASLIDFRPMDGL---GERAR

M. arenaria NLP-86 (41) LR----------SLANGRWQLRPGKRFVP-ENYYYQMMLDN---------

M. floridensis NLP-86 (41) LR----------SLANGRWQLSSPPHLKK-GSHQEKDSCQKT---ITIK-

M. hapla NLP-86 (43) LR----------SLANGRWQLRPGKRFVP-ESYYYQMMLDN---------

M. incognita NLP-86 (41) LR----------SLANGRWQLRPGKRFVP-ENYYYQMMLDN---------

M. javanica NLP-86 (41) LR----------SLANGRWQLRPGKRFVP-ENYYYQMMLDN---------

P. redivivus NLP-86 (151) MP---KR-----SLASGRWGLRPGKRS-------YDADLDDEDSHFAARF

P. univalens NLP-86 (64) R-----------SLASGRWGLRPGKRSQDAPNYVDELGFDGN---SPLYD

S. muris NLP-86 (37) ------------SLASGRWGLRPGKRSLGPLTPSDMFGGNGDGQFLLLPD

T. canis NLP-86 (50) QR----------SLASGRWGLRPGKRS-SDMAMSYVDDADGI---APLYD

201 250

A. lumbricoides NLP-86 (69) ALRQSHFVYVVRK-------------------------------------

A. suum NLP-86 (95) ALRQSHFVYVVRK-------------------------------------

B. xylophilus NLP-86 (88) ERRNLASGRWGLRPGKRSLANGRWGLRPGKRSMGMMPTQARHNPIMLLIP

G. pallida NLP-86 (82) RFRNLYALLPPLNNWN----------------------------------

G.rostochiensis NLP-86 (82) RFRNLYALLPPLNNWN----------------------------------

M. arenaria NLP-86 (71) --------------------------------------------------

M. floridensis NLP-86 (76) --------------------------------------------------

M. hapla NLP-86 (73) --------------------------------------------------

M. incognita NLP-86 (71) --------------------------------------------------

M. javanica NLP-86 (71) --------------------------------------------------

P. redivivus NLP-86 (186) PYRFVLVD------------------------------------------

P. univalens NLP-86 (100) ALRQPHFVYLVRK-------------------------------------

S. muris NLP-86 (75) AYDRPQHLYWWLG-------------------------------------

T. canis NLP-86 (86) TFRQQPVVYVVSKRK-----------------------------------

251

A. lumbricoides NLP-86 (82) --

A. suum NLP-86 (108) --

B. xylophilus NLP-86 (138) QL

G. pallida NLP-86 (98) --

G.rostochiensis NLP-86 (98) --

M. arenaria NLP-86 (71) --

M. floridensis NLP-86 (76) --

M. hapla NLP-86 (73) --

M. incognita NLP-86 (71) --

M. javanica NLP-86 (71) --

P. redivivus NLP-86 (194) --

P. univalens NLP-86 (113) --

S. muris NLP-86 (88) --

T. canis NLP-86 (101) --

NLP-87 Pan-phylum

Note: additional species *Ascaris lumbricoides, Plectus sambesi, Steinernema carpocapsae, Steinernema glaseri, Steinernema monticolum, Steinernema scapterisci, Toxocara canis.*

1 50

A. lumbricoides NLP-87 (1) --------------------------------------------------

A.suum NLP-87 (1) --------------------------------------------------

P. sambesi NLP-87 (1) MQVAHEWDEDGEIALFYFCVYDVFESRRPPRRADDNDAQALIICLQQTLP

S. carpocapsae NLP-87 (1) --------------------------------------------------

S. glaseri NLP-87 (1) ------------------------------MLRKEWEGRHAAFLDRVCEC

S. monticolum NLP-87 (1) --------------------------------------------------

S. scapterisci NLP-87 (1) --------------------------------------------------

T. canis NLP-87 (1) --------------------------------------------------

51 100

A. lumbricoides NLP-87 (1) --------------------------------------------------

A.suum NLP-87 (1) ---------------------------------------*MMCTWKCVLLV*

P. sambesi NLP-87 (51) STRRINIAFRLQKAQFHARTARRFAYDDDDDQDNNMAQTLALKLICVAMV

S. carpocapsae NLP-87 (1) ------------------------------------------------MT

S. glaseri NLP-87 (21) RTLSERTEAACVSTEGAYNHASYSLLFGGKSGPGDEWYFQCNGEPIETMS

S. monticolum NLP-87 (1) -----------------------------------------------MSP

S. scapterisci NLP-87 (1) ------------------------------------------------MT

T. canis NLP-87 (1) ---------------------------------------MMCIWKCVLVA

101 150

A. lumbricoides NLP-87 (1) -MVSVLSCDANLAVGRSSLRPSGKRSIALGRFSLRPGKR-----SIESTA

A.suum NLP-87 (12) *LMVSVLSCDA*NLAVGRSSLRPSGKRSIALGRFSLRPGKR-----SIGSFP

P. sambesi NLP-87 (101) VALVTEETAANVAIGRSGSVRPGKRSLALGRFNLRPGKR--------SII

S. carpocapsae NLP-87 (3) RSVLRFWLVLSFFALAFLLVTAEKRSIALGRLSLRPGKRADYSSTAQDIS

S. glaseri NLP-87 (71) PSLAVFRIIVFLLLVVYVCATYNKRSIALGRLSLRPGKRS-PFSSSDSLE

S. monticolum NLP-87 (4) SAMTMFQLFVTMLFVTCILANQNKRSIALGRLSLRPGKR--DSTIAPTME

S. scapterisci NLP-87 (3) RCVLIVRLVLSLLALTLLLVTAEKRSIALGRLSLRPGKRSGYSSTAQDLN

T. canis NLP-87 (12) LIVAVMFSEANLAVGRSSLRPSGKRSIALGRFSLRPGKR-----SVESAV

151 200

A. lumbricoides NLP-87 (45) QSLQTILGDKSGWDCSTQQLAMISDVMEGLASLLEDYTSYLGRCSALADI

A.suum NLP-87 (57) FK------------------------------------------------

P. sambesi NLP-87 (143) DSINAIDVDTNS--CNPSDIADVTNVMENLVPHLEKYVVYLERCTDGNSD

S. carpocapsae NLP-87 (53) DVLQFIRVPSVAPFCSGALVEAVTTNLEAVIRLLDKYSTYLERCNELGYD

S. glaseri NLP-87 (120) DLLNVVRLPAVAAQCSNAHVETITGGLKNLARLLDEYSFYLEKCRDLGYS

S. monticolum NLP-87 (52) DLIQVVRVPTIVSQCSNVHMEAVANTLDNLIRLMEQYSFYLEKCSDLGYS

S. scapterisci NLP-87 (53) DMLHFIRVPSVAPVCSNAQVEAVTRNLEAVIHLMDKYSTYLERCYELGYD

T. canis NLP-87 (57) QSLERMLQEKTEWECSTEQLGMMNNLMERAASLLEEYTSFLNACSLLSDI

201

A. lumbricoides NLP-87 (95) AGNFR

A.suum NLP-87 (59) -----

P. sambesi NLP-87 (191) K----

S. carpocapsae NLP-87 (103) IPI--

S. glaseri NLP-87 (170) T----

S. monticolum NLP-87 (102) V----

S. scapterisci NLP-87 (103) YFM--

T. canis NLP-87 (107) GSQL-

NLP-88

1 50

Ce-NLP-88 (1) -------------------------------------------MSILLIL

As-NLP-88 (1) ---------------------------------------*MKPTDKCLLFI*

Na-NLP-88 (1) -----------------------------------------*MLGPLLLSV*

Hc-NLP-88 (1) MGPFSPPFSRDWNPPTGGAVPEENSFPGLLNFPIAKKQEGKMISPLLLSL

Bx-NLP-88 (1) -----------------------------------*MKGLDMQIKHVLLFF*

Gp-NLP-88 (1) --MLSLPALSLVSFSENGRFRNFCLLLATLCLISGGRSLPLDRTNDRLFF

51 100

Ce-NLP-88 (8) LFCVVATSALVQLP--------RFSRYGIITNYDSPSRE---EMIVER--

As-NLP-88 (12) *VCLIAIIGAGNA*TP--------TDEINLSYLSSSLNGLHPLATWDMKNER

Na-NLP-88 (10) *AAFVPVLS*SIISSS--------QDSQLMNLLASTRPASEGYPEWILQSSR

Hc-NLP-88 (51) ATLVPIVSSILSSS--------QDSQLMNLLASSRGASDGYPEWILQSSK

Bx-NLP-88 (16) *LITMASVRTVQS*ASGSIYYYRDPPSAVLNPLSRFFYAEE---PRHLSPAK

Gp-NLP-88 (49) STVLRNPHSVNTLIPIG-----GVSPMFRFVGTNQFVGLKKRRERVGPIQ

101 150

Ce-NLP-88 (45) ----KRYDRNCFFSPVQCMLTYNDDSR------LPLVVTRKSSKRSIPLF

As-NLP-88 (54) RPIGKKFDRNCFFSPVQCMLSLNSNPA------DDMIWVRRK--------

Na-NLP-88 (52) A--VKKYDRNCFFSPVQCMLSFNQGQE------QPIVFTSKG-KR----L

Hc-NLP-88 (93) A--VKKYDRNCFFSPVQCMLSFNQGQE------QPIVFTSKG-KR----L

Bx-NLP-88 (63) R--DKKYDRNCFFSPVQCMLSYNTNNN------DALYHPRGR-K------

Gp-NLP-88 (94) PFLAKKYDRNCFFSPVQCMLSFKDGSNSMGSNSEVLHFEAGRRRRR----

151

Ce-NLP-88 (85) SDFRNLK

As-NLP-88 (90) -------

Na-NLP-88 (89) FYYRK--

Hc-NLP-88 (130) YYYRK--

Bx-NLP-88 (98) -------

Gp-NLP-88 (140) -------

NLP-92

1 50

As-NLP-92 (1) ----------------------------IIALKLYCDFFIEATNCRPYAP

Bm-NLP-92 (1) MVLALRKQTSPIFLSAVFLLCFPICNHATLLTHIIHGRLFDIDPSSLFAR

Di-NLP-92 (1) MVLTSWKKIFTYSPISAISTMLSHLQSCNIPNTYCSQQIFDTDPLSLIGR

51 98

As-NLP-92 (23) YPPVAPLTKLRSGA-LRNCFFSPVQCLLPFNNKSMRRLDEKRTAFNE-

Bm-NLP-92 (51) ELKLIHLKKQRSNN-LRNCFFSPVQCLLPFDDGRSVESEAKRRS----

Di-NLP-92 (51) ELKLTYQKKLRSNNNLRKCFFSPVQCLLPFDCSRSMESDNKKRIGSYD

NLP-92 Pan-phylum

Note: additional species *Ascaris lumbricoides, Anisakis simplex, Acanthocheilonema viteae, Brugia phangai, Brugia timori, Elaeophora elaphi, Gongylonema pulchrum, Onchocerca flexuosa, Onchocerca ochengi, Onchocerca volvulus, Toxocara canis, Wuchereria bancrofti*.

1 50

A. lumbricoides NLP-92 (1) MKAMSSWITGSMLWLAVLLLLDGQHAESLPTVDFEDARFIDGTEFNARNA

A. simplex NLP-92 (1) --------------------------------------------------

A. suum NLP-92 (1) --------------------------------------------------

A. viteae NLP-92 (1) --------------------------------------------------

B. malayi NLP-92 (1) ----------------------------------------MVLALRKQTS

B. phangai NLP-92 (1) --------------------------------------------------

B. timori-NLP-92 (1) --------------------------------------------------

D. immitis NLP-92 (1) ----------------------------------------MVLTSWKKIF

E. elaphi NLP-92 (1) --------------------------------------------------

G. pulchrum NLP-92 (1) --------------------------------------------------

O. flexuosa NLP-92 (1) ----------------------------------------MVLTSWKQSS

O. ochengi NLP-92 (1) --------------------------------------------------

O. volvulus NLP-92 (1) ----------------------------------------MVLTLWKQSS

T. canis NLP-92 (1) -----------------------------MNNDNPKVAGRAGHSNNSK-V

W. bancrofi NLP-92 (1) --------------------------------------------------

51 100

A. lumbricoides NLP-92 (51) NLTHTEGANREGNTSKRVSILYCDFFIEATNCRPYAPYPPVAP---LTKL

A. simplex NLP-92 (1) -----------------MLSVVCSFLEKRLYIGSYRSYGPRFYP-LLNKR

A. suum NLP-92 (1) ---------------IIALKLYCDFFIEATNCRPYAPYPPVAP---LTKL

A. viteae NLP-92 (1) ----------------MLCDKTPKTFYRLFDIDPSSLFTKEFKLIQSTKQ

B. malayi NLP-92 (11) PIFLSAVFLLCFPICNHATLLTHIIHGRLFDIDPSSLFARELKLIHLKKQ

B. phangai NLP-92 (1) ------------------------MFYRLFDIDPSSLFARELKLIHLKKQ

B. timori-NLP-92 (1) ---------------------------RLFDIDPSSLFARELKLIHLKKQ

D. immitis NLP-92 (11) TYSPISAISTMLSHLQSCNIPNTYCSQQIFDTDPLSLIGRELKLTYQKKL

E. elaphi NLP-92 (1) ------------------------------------------------KQ

G. pulchrum NLP-92 (1) ---------------------YNLICYRTFGYYPSLPVD---DLEYVVKR

O. flexuosa NLP-92 (11) PLLLSALLLICFPICNHATLLTHIVRGRSFDVDPLSIVGRELKVIQPEKQ

O. ochengi NLP-92 (1) ---------------------------RSFDVDPLSIVGRELKVIHPEKQ

O. volvulus NLP-92 (11) PLLLSAVLLICFPICSHATLLTHIVRGRSFDVDPLSIVGRELKVIHPEKQ

T. canis NLP-92 (21) MSSWILGSVVWTAMLLILTDQQTNAFPTMILDDPRPYMPPNLDGLIVPKR

W. bancrofi NLP-92 (1) ---------------------------RLFDIDPSSLFARELKLIHLKKQ

101 138

A. lumbricoides NLP-92 (98) RS-GALRNCFFSPVQCLLPFNN-KSMRRLDEKRTAFNE

A. simplex NLP-92 (33) RN-AALRNCFFSPVQCLLPFDN-RSLRRFTDQSITDFD

A. suum NLP-92 (33) RS-GALRNCFFSPVQCLLPFNN-KSMRRLDEKRTAFNE

A. viteae NLP-92 (35) RSKNNLRNCFFSPVQCLLPFDDSRLMQSNSKRRTEFPD

B. malayi NLP-92 (61) RS-NNLRNCFFSPVQCLLPFDDGRSVESEAKRRS----

B. phangai NLP-92 (27) RS-NNLRNCFFSPVQCLLPFDDGRSVESDAKRRS----

B. timori-NLP-92 (24) RS-NNLRNCFFSPVQCLLPFDDGRSVESDAKRRS----

D. immitis NLP-92 (61) RSNNNLRKCFFSPVQCLLPFDCSRSMESDNKKRIGSYD

E. elaphi NLP-92 (3) QNMNALRNCFLSPVQCLLPFD-----------------

G. pulchrum NLP-92 (27) RSKNSLRNCFFSPVQCLLPFDNSRSMRRIGEKRAENYD

O. flexuosa NLP-92 (61) RSSNNLRNCFFSPVQCLLPFDSSRSMENDIKKRIEFFY

O. ochengi NLP-92 (24) RS-NNLRNCFFSPVQCLLPFDSSRSMENDIKKR-----

O. volvulus NLP-92 (61) RS-NNLRNCFFSPVQCLLPFDSSRSMENDIKKRIEFFY

T. canis NLP-92 (71) RD-GGLRNCFFSPVQCLLPFDN-KSLRRFGEKRTIGFQ

W. bancrofi NLP-92 (24) RS-NNLRNCFFSPVQCLLPFGDGRSVESNTKRRS----

NLP-93

1 50

As-NLP-93 (1) ---------------------------------LYALRLDGNLSK-----

Na-NLP-93 (1) -----------------------------MSHIKRVTMCCFRFRK-----

Hc-NLP-93 (1) --------*MLSLRSLATIFFVLVVVASS*QRDHLSYYSQPDLRSSS-----

Bx-NLP-93 (1) *MQMLVILLLLNFISA*NPALRSMPQLNPVMRYYRLKSQPADENSLPNERPV

51 100

As-NLP-93 (13) RFRKQSRNSRNCFFSPIQCHLPVAAINDMETLSKRRFRSKPW--------

Na-NLP-93 (17) --ESDSQKYRNCYFSPVQCQLPVLINIDPLASAIKPDLQQDIYTRFRRYR

Hc-NLP-93 (38) EYTKRRAALRNCFLSPMQCLLPVDNLRFNKFHKRFFFAK-----------

Bx-NLP-93 (51) RATPLASSGRNCFFSPVQCRLPVSKVRSMDSSGQRLFLNDQPGLGIFDRM

101

As-NLP-93 (55) ------

Na-NLP-93 (65) K-----

Hc-NLP-93 (77) ------

Bx-NLP-93 (101) RRWNKY

NLP-93 Pan-phylum

Note: additional species *Ascaris lumbricoides, Anisakis simplex, Ditylenchus destructor, Haemonchus placei, Heligmosomoides polygyrus, Oscheius tipulae, Pristionchus exspectatus, Pristionchus pacificus, Plectus sambesii, Steinernema glaseri, Toxocara canis*.

1 60

A. lumbricoides NLP-93 (1) --------------------------------MAQSLMMGYERLFLLSIFISAVICNESS

A. simplex NLP-93 (1) ------------------------------------------------------------

A. suum NLP-93 (1) --------------------------------------------------LYALRLDG--

B. xylophilus NLP-93 (1) --------------------------MQMLVILLLLNFISANPALRSMPQLNPVMRYYRL

D. destructor NLP-93 (1) -------MALSSKMLWDSVRWSHVITAIFVLTTFIVICPTTSVASPLARYQLGRSSTDFA

H. contortus NLP-93 (1) ------------------------------MLSLRSLATIFFVLVVVASSQRDHLSYYSQ

H. placeii NLP-93 (1) -----------------------------------------------------FSRLNSP

H. polygyrus NLP-93 (1) --------------------------MKFVQLIALLCFLSPLYSRVIQGDANYYYKVLRM

N. americanus NLP-93 (1) ------------------------------------------------------MSHIKR

O. tipulae NLP-93 (1) -----------------------------MRKDLEGCDIRVHLTEVCFRYWAQNRLRRKL

P. exspectatus NLP-93 (1) ------------------------------------------------------------

P. pacificus NLP-93 (1) ---------------------MSSSSSSTISSLLFVLISLSLLLSTAQAYPSYVYADDGR

P.sambesii NLP-93 (1) MTANSERVFILLAVVAVVGVFAEIQTSDTQQRLNNYDQRDMTRLYRSMSKIGAIKDINEW

S. glaseri NLP-93 (1) ---MSLDRLGNVGPNWLAVLLMISLLHEGLSTSPKNTASLIESMNPIVRYRFTAHLKNRL

T. canis NLP-93 (1) ------------------------------------------------------------

61 120

A. lumbricoides NLP-93 (29) DQFEDMNPILRKQ----------SRNSRNCFFSPIQCHLPVAAIND-------METLSKR

A. simplex NLP-93 (1) -----MLKSFRKQ----------SKNSRNCFFSPVQCQLPVPAIDQ-------MENLSKR

A. suum NLP-93 (9) ----NLSKRFRKQ----------SRNSRNCFFSPIQCHLPVAAIND-------METLSKR

B. xylophilus NLP-93 (35) KSQPADENSLPNERPV--RATPLASSGRNCFFSPVQCRLPVSKVRSMDSSGQRLFLNDQP

D. destructor NLP-93 (54) PTWDVILVKRAKNNMSPSQIAPSIRNNRNCYFSPVQCLLPVNTDVLR------SFSSDRY

H. contortus NLP-93 (31) PDLRSSSEYTKRR-----------AALRNCFLSPMQCLLPVDNLRFN--------KFHKR

H. placeii NLP-93 (8) PSPPSHSEAASRL----------LSLDRNCFFSPVQCMLPHNSMQQ-------TI-----

H. polygyrus NLP-93 (35) TNPGASHSIASRA----------PSLDRNCFFSPVQCMLPVTMQHP-------I------

N. americanus NLP-93 (7) VTMCCFRFRKESD----------SQKYRNCYFSPVQCQLPVLINIDPLASAIKPDLQQDI

O. tipulae NLP-93 (32) PLLDAMAAKNRQEG-------GDSVKYRNCFFSPVQCQLPVTVDKDPLVTALHIPNQAAF

P. exspectatus NLP-93 (1) ---YFRMTTPRKP----------EFLRRNCFFSPVQCQFPVSQGVN-------ELRFRRK

P. pacificus NLP-93 (40) SDFNVPVVRRSPS----------HSAMRNCFFSPVQCMMPMQSVNFN--------GFHKR

P.sambesii NLP-93 (61) KTKTANAPNKNLPPLQPSLADLSQGNIRNCYFSPVQCLLPVGKRSDAFPKLDFSTDLSRF

S. glaseri NLP-93 (58) QQLDSNSADRQKP----------NRNSRNCFFSPVQCQLPVEVASQQRPDNLYVVNKFRK

T. canis NLP-93 (1) ------NFNFRKQ----------SLNSRNCFFSPVQCQLPVTAIDD-------METLSKR

121 137

A. lumbricoides NLP-93 (72) RFRSKPW----------

A. simplex NLP-93 (39) WQWMRPS----------

A. suum NLP-93 (48) RFRSKPW----------

B. xylophilus NLP-93 (93) GLGIFDRMRRWNKY---

D. destructor NLP-93 (108) -----------------

H. contortus NLP-93 (72) FFFAK------------

H. placeii NLP-93 (46) -----------------

H. polygyrus NLP-93 (72) -----------------

N. americanus NLP-93 (57) YTRFRRYRK--------

O. tipulae NLP-93 (85) DRFRRLGWGRK------

P. exspectatus NLP-93 (41) L----------------

P. pacificus NLP-93 (82) LN---------------

P.sambesii NLP-93 (121) LKFLGARNSIDSE----

S. glaseri NLP-93 (108) RLSSLPFFSASALGLVD

T. canis NLP-93 (38) RLRAKPW----------

NLP-94 Pan-phylum

Note: additional species *Ascaris lumbricoides, Anisakis simplex, Toxocara canis*.

1 50

A. lumbricoides NLP-94 (1) ---------MHGNNRIGVTVAFVICSHIFGN-AMLITHENVMKPFAIGAI

A. simplex NLP-94 (1) --------MYYNKQQIKIILAFIICAIGYGTNALPLTSMYMLRPLELSEM

A. suum NLP-94 (1) MNECMHCRFMHGNNRIGVTVAFVMCSHIFGN-AMLITHENVMKPFAIGAI

T. canis NLP-94 (1) -----------------MSITLLLKTQLHAI-TQRKVCFNKRATLGINNM

51 97

A. lumbricoides NLP-94 (41) EEQPMNLAYEDNSES---MNAEENAFRRNCFFSPVQCMFRRSTT---

A. simplex NLP-94 (43) NDNAVSLDYNEDELTNSSLEGASLTFKRNCFFSPVQCMFRRSSSK--

A. suum NLP-94 (50) EEQPMNLAYEDNSES---MNAEENAFRRNCFFSPVQCMFRRSTT---

T. canis NLP-94 (33) EERPMHIAYDDDLTP---FYVKSDSFRRNCFFSPVQCMFRRSVDYSM

NLP-95

1 60

As-NLP-95 (1) ------------------------------------------------------------

Bx-NLP-95 (1) ------------------------------------------------------------

Gp-NLP-95 (1) MIRRNSSPISAHFLANFGTSMAAFLLIVVVACSLASHPFTTGTYRYVPKHNFGALYDGGR

61 120

As-NLP-95 (1) ---------------------------------------------------------GAL

Bx-NLP-95 (1) ---*MLTYCRFVILIVGVNVISLSA*QSRAATWKSALNFGPDFEKDWIQMGESSEKREPNAP

Gp-NLP-95 (61) PHLPRSASTFFASSQIPDRTVSSLFGAVPDYLRHAGGVSPSDLSGVQLFVPETIAADEMP

121 180

As-NLP-95 (4) IPFQNILQEMFSDNEGEIVEESSRLGNKNCFFSLVQCSFYYR------------------

Bx-NLP-95 (58) RPKPEIAKESLRGMMSDEPIGSGKLASKNCFFSPVQCSFFYKRNYDPFPYSRFV------

Gp-NLP-95 (121) QIGGAELKQNDLPSTDLHGDQKGQLARKNCFFSPLQCSFYYKKRSHHGRNAPGGAIFAPG

181

As-NLP-95 (46) --------

Bx-NLP-95 (112) --------

Gp-NLP-95 (181) TVIRSDLF

NLP-95 Pan-phylum

Note: additional species *Ascaris lumbricoides, Anisakis simplex, Ditylenchus destructor, Globodera rostochiensis, Meloidogyne arenaria, Meloidogyne floridensis, Meloidogyne hapla, Meloidogyne incognita, Meloidogyne javanica, Parascaris equorum, Panagrellus redivivus, Rhabditophanes spp., Steinernema monticolum, Steinernema feltiae, Toxocara canis*.

1 50

A. lumbricoides NLP-95 (1) --------------------------------------------------

A. simplex NLP-95 (1) --------------------------------------------------

A. suum NLP-95 (1) --------------------------------------------------

B. xylophilus NLP-95 (1) --------------------------------------------------

D. destructor NLP-95 (1) --------------------------------------------------

G. pallida NLP-95 (1) ------MIRRNSSPISAHFLANFGTSMAAFLLIVVVACSLASHPFTTGTY

G. rostochiensis NLP-95 (1) ------MNRRNSSPISAHFLANFGTSITAFFLIVVVACSMASHPFTTGTY

M. arenaria NLP-95 (1) MSTKQQQQKSFSSSSINYSTQIFFYFRIIFIYYFLAIQKLDAYPNFYNNE

M. floridensis NLP-95 (1) MSTKQQQQKAFSSSSINYSTQIFFYFRIIFIYYFLTIQKLDAYPNFYNNE

M. hapla NLP-95 (1) --------------------------------------------------

M. incognita NLP-95 (1) MSTKQQQQKSFSSSSINYSTQIFFYFRIIFIYYFLAIQKLDAYPNFYNNE

M. javanica NLP-95 (1) MSTKQQQQ-FSSSSSIKYSTQIFFYFRIIFIYYFLAIQKLDAYPNFYNNE

P. equorum NLP-95 (1) --------------------------------------------------

P. redivivus NLP-95 (1) --------------------------------------------------

Rhabditophanes sp. NLP-95 (1) --------------------------------------------------

S. monticolum NLP-95 (1) --------------------------------------------------

S. feltiae NLP-95 (1) --------------------------------------------------

T. canis NLP-95 (1) --------------------------------------------------

51 100

A. lumbricoides NLP-95 (1) ----------------------------------------------MFQQ

A. simplex NLP-95 (1) --------------------------------------------------

A. suum NLP-95 (1) --------------------------------------------------

B. xylophilus NLP-95 (1) ----------------------------------MLTYCRFVILIVGVNV

D. destructor NLP-95 (1) --------------------------------------------------

G. pallida NLP-95 (45) RYVPKHNFGALYDGG------------RPHLPRSASTFFASSQIPDRTVS

G. rostochiensis NLP-95 (45) RYVPKHNFGALYDGG------------RPHLPRSASTFFASSQTPYRTVN

M. arenaria NLP-95 (51) LLPMQNMRKFLSQGGGEEKNEGENNGNLNNLKEENKLNDGNHPVFFGRKI

M. floridensis NLP-95 (51) LP-IQNVRKFLSQGG-EEKNEGENNGNFNNLKEENKLNDGNHPVFFGRKI

M. hapla NLP-95 (1) -----------------------------------------------XPI

M. incognita NLP-95 (51) LLPMQNMRKFLSQGGGEEKNEGENNGNLNNLKEENKLNDGNHPVFFGRKI

M. javanica NLP-95 (50) LLPIQNVRKFLSQGG-EEKNEGENNGNLNNLKEENKLNDGNHPVFFGRKV

P. equorum NLP-95 (1) --------------------------------------------------

P. redivivus NLP-95 (1) -------------------------------MQQYVLLIAAAFIFVVASE

Rhabditophanes sp. NLP-95 (1) ----------------------MLCGIKMIVTMMFFSLMGSALTINHSRL

S. monticolum NLP-95 (1) --------------MSPNQLVQFALVLVGFVCLTTLAQIDESDSSRVAID

S. feltiae NLP-95 (1) --------------------------------------------------

T. canis NLP-95 (1) -----------------------------MLYHAAVMLPLIASLLSLSEQ

101 150

A. lumbricoides NLP-95 (5) INFDKCIKRAKKEAILPVISCSNLYKKRTGLQNEINRFQ----------D

A. simplex NLP-95 (1) ------------------------------MGMSEWDDK----------N

A. suum NLP-95 (1) --------------------------------GALIPFQ----------N

B. xylophilus NLP-95 (17) ISLSAQSRAATWKSALNFGPDFEKDWIQMGESSEKREPN-----APRPKP

D. destructor NLP-95 (1) -------------------------------------------------M

G. pallida NLP-95 (83) SLFGAVPDYLRHAGGVSPSDLSGVQLFVPETIAADEMP--------QIGG

G. rostochiensis NLP-95 (83) SLFGAVPDYLRHAGSVSPSDLSGVQLFVPETIPADEMP--------QIGG

M. arenaria NLP-95 (101) SSLGDLQQTFTEGLLETPSSGFVRSAANKLIKKDKEEN----------GI

M. floridensis NLP-95 (99) SSLGDLQQTFTE--LETPSSGFVRSAANKLIKKDKEEN----------GI

M. hapla NLP-95 (4) SSLGDLQQTFTEGLLETPSSGFVRSAANKLMKKAEKEE---KENKKENEI

M. incognita NLP-95 (101) SSLGDLQQTFTEGLLETPSSGFVRSAANKLIKKDKEEN----------GI

M. javanica NLP-95 (99) SSLGDLQQTFTE--LETPSSTFVRSAANKLIKKDKEEIE-EKENKKENGI

P. equorum NLP-95 (1) --------------------------------------------------

P. redivivus NLP-95 (20) SVQNSPVDFRPYRRFNDLESQFEENLLAPLVKRPFERRDGAVIGARRPSF

Rhabditophanes sp. NLP-95 (29) NRMHIRGIIEQEARIEQLRDSLSNNYLSQYNSNSAPRSDP--PKRTMSLD

S. monticolum NLP-95 (37) PAALARYRIYAYGNLLKRSDEGSLG-TSKLSFFTDKRRA----------D

S. feltiae NLP-95 (1) --RHQNPLTNDYRNLLKRSAEGQLGSPAKLSLFTDKRRA----------D

T. canis NLP-95 (22) KSIAMTEIRNSEPLFNSLSYLSSLYNKRTGIQSEASRVQ----------Q

151 200

A. lumbricoides NLP-95 (45) ILRGSKRKDNEGEIVEESSRLGNKNCFFSLVQCSFYYR------------

A. simplex NLP-95 (11) GRCIAHISENDAEIMESSSRFGNKNCFFSLVQCSFYY-------------

A. suum NLP-95 (9) ILQ-EMFSDNEGEIVEESSRLGNKNCFFSLVQCSFYYR------------

B. xylophilus NLP-95 (62) EIAKESLRGMMSDEPIGSGKLASKNCFFSPVQCSFFYKRNYDPFPYSRFV

D. destructor NLP-95 (2) PEFIARDSFLRDETIKPTSRLARKNCFMSPLQCSFYYKRSNSFF------

G. pallida NLP-95 (125) AELKQNDLPSTDLHGDQKGQLARKNCFFSPLQCSFYYKKRSHHGRNAPGG

G. rostochiensis NLP-95 (125) AELKQNDLPLTDLHGDQKGQLARKNCFFSPLQCSFYYKKRSHHRRSAPGG

M. arenaria NLP-95 (141) INTIITNNRQPRAN-NYNSKLARKNCFFSPLQCSFYYKSNSNFANMLMRG

M. floridensis NLP-95 (137) INTLITNNRQPRANSNYNSKLARKNCFFSPLQCSFYYKSNSNFANMLMRG

M. hapla NLP-95 (51) INTIITNNRQPRAN---NAKLARKNCFFSPLQCSFYYKSNSNFANMLIRG

M. incognita NLP-95 (141) INTIITNNRQPRAN-NYNSKLARKNCFFSPLQCSFYYKSNSNFANMLMRG

M. javanica NLP-95 (146) INTIITNNRQPRAN-NYNSKLARKNCFFSPLQCSFYYKSNSNFANMLMRG

P. equorum NLP-95 (1) ----EMFSDNEGEIADDSSRLGSKNCFFSLVQCSFYYR------------

P. redivivus NLP-95 (70) VKSGIVRDIVNDEAGKSSSRFARKNCFFSPVQCSFYYKRSVNLF------

Rhabditophanes sp. NLP-95 (77) AFSAYHDLSQGPYVQPSGSKYSTKNCFLSPVQCSFFYYRK----------

S. monticolum NLP-95 (76) GRSSAGLQPGSIRSFSGTPKFSSKNCFFSPVQCSFYYRR-----------

S. feltiae NLP-95 (39) GRGGAGLQPGSIRSFSGTPKFSSKNCFFSPVQCSFYYRR-----------

T. canis NLP-95 (62) ILSGAKRKDSESEIVEGRTRFGNKNCFFSLVQCSFYYR------------

201 214

A. lumbricoides NLP-95 (83) --------------

A. simplex NLP-95 (48) --------------

A. suum NLP-95 (46) --------------

B. xylophilus NLP-95 (112) --------------

D. destructor NLP-95 (46) --------------

G. pallida NLP-95 (175) AIFAPGTVIRSDLF

G. rostochiensis NLP-95 (175) AIFTPGTVISSNLF

M. arenaria NLP-95 (190) DE------------

M. floridensis NLP-95 (187) DE------------

M. hapla NLP-95 (98) DE------------

M. incognita NLP-95 (190) DE------------

M. javanica NLP-95 (195) DE------------

P. equorum NLP-95 (35) --------------

P. redivivus NLP-95 (114) --------------

Rhabditophanes sp. NLP-95 (117) --------------

S. monticolum NLP-95 (115) --------------

S. feltiae NLP-95 (78) --------------

T. canis NLP-95 (100) --------------

NLP-96 Pan-phylum

Note: additional species *Ancylostoma caninum, Angiostrongylus cantonensis, Ancylostoma ceylanicum, Angiostrongylus costaricensis, Ancylostoma duodenale, Dictyocaulus viviparus, Heterorhabditis bacteriophora, Haemonchus placei, Heligmosomoides polygyrus, Nippostrongylus brasiliensis, Oesophagostomum dentatum, Strongylus vulgaris, Teladorsagia circumcincta.*

1 50

A. caninum NLP-96 (1) --------------------------------------------------

A. cantonensis NLP-96 (1) --------------------------------------------------

A. ceylanicum NLP-96 (1) --------------------------------------------------

A. costaricensis NLP-96 (1) --------------------------------------------------

A. duodenale NLP-96 (1) --------------------------------------------------

D. viviparus NLP-96 (1) --------------------------------------------------

H. bacteriophora NLP-96 (1) --------------------------------------------------

H. contortus NLP-96 (1) --------------------------------------------------

H. placei NLP-96 (1) --------------------------------------------------

H. polygyrus NLP-96 (1) --------------------------------------------------

N. brasiliensis NLP-96 (1) MEARTEKTVVGSREEFRNNSDIGEAKPSLDHNVVYFVVVQSTWRIRRSSI

O. dentatum NLP-96 (1) -------------------------GSKIHKIKILLELQLPFCVYRRSPC

S. vulgaris NLP-96 (1) --------------------------------------------------

T. circumcincta NLP-96 (1) --------------------------------------------------

51 100

A. caninum NLP-96 (1) -------------MLVVPLILLFCVFSQLPARVVVGDANYYYKVLRLANP

A. cantonensis NLP-96 (1) ---------------------------------------RNWKLFRLASS

A. ceylanicum NLP-96 (1) -------------MLVVPLIVLLCVFSQLHARVVVGDANYYYKVLRLANP

A. costaricensis NLP-96 (1) -------------------------------------PLVRNDYALGYCS

A. duodenale NLP-96 (1) ----------------------ALKEQRVGLHRAQFNNFNDSTRFRLANI

D. viviparus NLP-96 (1) -----------------------------------------YVCFRLTSP

H. bacteriophora NLP-96 (1) ---------------------------------------NLIYLFRMHSP

H. contortus NLP-96 (1) -------------MRVVQAIAFFCLVSQLYSRVVVGDANYYFKVLRLNSP

H. placei NLP-96 (1) -------------------------------------------FSRLNSP

H. polygyrus NLP-96 (1) -------------MKFVQLIALLCFLSPLYSRVIQGDANYYYKVLRMTNP

N. brasiliensis NLP-96 (51) EDGNSLELQHTKVMSIAQVVLLFCLVSQLSSRVIQGDANYYFKVLRMANP

O. dentatum NLP-96 (26) SSETQALLETLRRFNIRQYIRIGHRSKLVCAVRFKCLVAYLILHFRMPSP

S. vulgaris NLP-96 (1) -------------------------------LILSCAQINEFLFFRLADS

T. circumcincta NLP-96 (1) ---------------------------------------NVRFYRLADPT

101 147

A. caninum NLP-96 (38) G---ASHSIASRTPSLDRNCFFSPVQCMLPASGTIPQAI--------

A. cantonensis NLP-96 (12) N---TPHFIESRNPSLDRNCFFSPVQCMLVN----RSP---------

A. ceylanicum NLP-96 (38) G---ASHSIASRTPSLDRNCFFSPVQCMLPASGTVPQAI--------

A. costaricensis NLP-96 (14) N---TPHFIESRNPSLDRNCFFSPVQCMLTPPQKHKQPWVIDSGRRL

A. duodenale NLP-96 (29) G---ASHSIASRTPSLDRNCFFSPVQCMLPASGPIQAI---------

D. viviparus NLP-96 (10) N---VSHLVASRNPSLDRNCFFSPVQCMFSMSRTP------------

H. bacteriophora NLP-96 (12) G---KAHTIASRTPKLDRNCFFSPVQCMLSSNNALNSF---------

H. contortus NLP-96 (38) PSPPSHSEPASRLLSLDRNCFFSPVQCMLPHNSMQQTI---------

H. placei NLP-96 (8) PSPPSHSEAASRLLSLDRNCFFSPVQCMLPHNSMQQTI---------

H. polygyrus NLP-96 (38) G---ASHSIASRAPSLDRNCFFSPVQCMLPVTMQHPI----------

N. brasiliensis NLP-96 (101) G---ASHSIASRAPSLDRNCFFSPVQCMLPLNSMQQPL---------

O. dentatum NLP-96 (76) S---TSHLIASRAPSFDRNCFFSPVQCMLPSNNRLQQAV--------

S. vulgaris NLP-96 (20) G---LSHYVASRAPSFDRNCFFSPVQCMLPFNRIHRAE---------

T. circumcincta NLP-96 (12) T---QAHYVASRAPSLDRNCFFSPVQCMLSNGKQAQEI---------

NLP-97

1 50

As-NLP-97 (1) -----------MCESGCSGKHHVKAYLARATPLVMFGVYVSK------RS

Bm-NLP-97 (1) ----------------------MCHYHIISYHFIIISIIIIP------TI

Di-NLP-97 (1) -------MLYLMNPNIGNVKMDRYRYSIPFYSIIIISTIIIA------TT

Na-NLP-97 (1) ---------------------------*MSSLSFLFVLLLVYT------IS*

Hc-NLP-97 (1) ---------------------------*MSPLSLLCALLVVYT------AL*

Bx-NLP-97 (1) MEHVNHPRVERRGEARGWATPFGGNGGLFARSLIVNCYLDKGRQVGNYST

Gp-NLP-97 (1) -------MSSFTFTSSTTGFIFSALFVSASFAFPPFVGQKAEQDKRGFPV

51 100

As-NLP-97 (34) RNPYSWMNEEKRMDNPYAWTNVNENMLGLD----LDELEKRARNPYSWMN

Bm-NLP-97 (23) ANPAIGNIYEKPS-KYFTYKNEPDNYGFMP----EEYFNTAKRSRNSYSW

Di-NLP-97 (38) ASPTN-DIYDKSSNHFALYKNEPNNDDSML----DEYFVADKRFQN----

Na-NLP-97 (18) *ADA*VN----------EIQGTNEDENAIDIN----EEKRVHS-RNPYSWMS

Hc-NLP-97 (18) *A*DVND----------EAEQHDRDLLQLNDD----GISSVKRARNPYSWMV

Bx-NLP-97 (51) RDSVNGFGDMALLRPSIVVALTLVVVYGLDSFETTKRRLGRPMNPYSWQN

Gp-NLP-97 (44) RNPYSWMAQLQKRSVPSGMDNTSPSDGELPNLLLEEPSKRFPSPAASFGG

101 150

As-NLP-97 (80) YKEKRALRNPYSWMAFG-----------EKRAPLNPYSWMYTLQKRAPFG

Bm-NLP-97 (68) MSNSAIKSQPRNMLIVL-----------EKPAPLNPYSWQYMMKKKNQI-

Di-NLP-97 (79) --DKGIKLPP--VLIAF-----------EKPVPMNPYSWQYMVKKKSGK-

Na-NLP-97 (53) --QEKRARNPYSWMVS------------EGKRSRNPYSWMAMEKEKYYLN

Hc-NLP-97 (54) VDQSKRSRNPYSWMVHSKGKRSLRYFDDAYRQPRNPYSWLYTMFDKRAR-

Bx-NLP-97 (101) -GGYKIKRSLENELETETGALPE---EKRAPMPRNPYSWLARSQKEGPYQ

Gp-NLP-97 (94) ----RRPQNPYSWMSFGDADQSNMDWLFTRLRPFSELVSKRLNALGMGMG

151 198

As-NLP-97 (119) --YFRNPYSWLNSPASW-------------------------------

Bm-NLP-97 (106) --ILRNPYSWMNNHNN--------------------------------

Di-NLP-97 (113) --IARNPYSWMNNIY---------------------------------

Na-NLP-97 (89) --YRLLSGQTCASFFR--------------------------------

Hc-NLP-97 (103) ----N-PYSWMNE-----------------------------------

Bx-NLP-97 (147) QKRTRDAYFWNNQDFLLDSLFRSETKKSPYVGFNGRRARNPYSWMNFV

Gp-NLP-97 (140) FRRPKNPYLWAMNRQLLAE-----------------------------

NLP-97 Pan-phylum

Note: additional species *Ancylostoma caninum, Ancylostoma ceylanicum, Angiostrongylus costaricensis, Ancylostoma duodenale, Ascaris lumbricoides, Anisakis simplex, Ditylenchus destructor, Dracunculus medinensis, Dictyocaulus viviparus, Enterobius vermicularis, Heterorhabditis bacteriophora, Haemonchus placei, Heligmosomoides polygyrus, Oesophagostomum dentatum, Pristionchus pacificus, Panagrellus redivivus, Steinernema carpocapsae, Steinernema feltiae, Steinernema monticolum, Syphacia muris, Steinernema scapterisci, Strongylus vulgaris, Toxocara canis, Teladorsagia circumcincta.*

1 50

A. caninum NLP-97 (1) --------------------------------------------------

A. ceylanicum NLP-97 (1) --------------------------------------------------

A. costaricensis NLP-97 (1) --------------------------------------------------

A. duodenale NLP-97 (1) --------------------------------------------------

A. lumbricoides NLP-97 (1) --------------------------------------------------

A. simplex NLP-97 (1) --------------------------------------------------

A. suum NLP-97 (1) --------------------------------------------------

B. malayi NLP-97 (1) --------------------------------------------------

B. xylophilus NLP-97 (1) --------------------------------------------------

D. destructor NLP-97 (1) --------------------------------------------------

D. immitis NLP-97 (1) --------------------------------------------------

D. medinensis NLP-97 (1) --------------------------------------------------

D. viviparus NLP-97 (1) --------------------------------------------------

E. vermicularis NLP-97 (1) --------------------------------------------------

G. pallida NLP-97 (1) --------------------------------------------------

H. bacteriophora NLP-97 (1) MLHASPSNSDTTRVDSPIVGYEASTRHMDDAFDHHIPIHAVGPSRPHIQP

H. contortus NLP-97 (1) --------------------------------------------------

H. placei NLP-97 (1) --------------------------------------------------

H. polygyrus NLP-97 (1) --------------------------------------------------

N. americanus NLP-97 (1) --------------------------------------------------

O. dentatum NLP-97 (1) --------------------------------------------------

P. pacificus NLP-97 (1) --------------------------------------------------

P. redivivus NLP-97 (1) --------------------------------------------------

S. carpocapsae NLP-97 (1) --------------------------------------------------

S. feltiae NLP-97 (1) --------------------------------------------------

S. monticolum NLP-97 (1) --------------------------------------------------

S. muris NLP-97 (1) --------------------------------------------------

S. scapterisci NLP-97 (1) -------------------MLKGYVCSKPVLSMRHLSDYVHVELSFSTFF

S. vulgaris NLP-97 (1) --------------------------------------------------

T. canis NLP-97 (1) --------------------------------------------------

T. circumcincta NLP-97 (1) --------------------------------------------------

51 100

A. caninum NLP-97 (1) --------------------------------------------------

A. ceylanicum NLP-97 (1) --------------------------------------------------

A. costaricensis NLP-97 (1) --------------------------------------------------

A. duodenale NLP-97 (1) --------------------------------------------------

A. lumbricoides NLP-97 (1) --------------------------------------------------

A. simplex NLP-97 (1) --------------------------------------------------

A. suum NLP-97 (1) --------------------------------------------------

B. malayi NLP-97 (1) --------------------------------------------------

B. xylophilus NLP-97 (1) -----------------------------------------------MEH

D. destructor NLP-97 (1) -------------------------------------------------M

D. immitis NLP-97 (1) --------------------------------------------------

D. medinensis NLP-97 (1) --------------------------------------------------

D. viviparus NLP-97 (1) --------------------------------------------------

E. vermicularis NLP-97 (1) --------------------------------------------------

G. pallida NLP-97 (1) --------------------------------------------------

H. bacteriophora NLP-97 (51) SKNVRMTMISRDEESVLFAAMKFANTVRRRQYRGQCEDDLLIFLSTKDKV

H. contortus NLP-97 (1) --------------------------------------------------

H. placei NLP-97 (1) --------------------------------------------------

H. polygyrus NLP-97 (1) --------------------------------------------------

N. americanus NLP-97 (1) --------------------------------------------------

O. dentatum NLP-97 (1) --------------------------------------------------

P. pacificus NLP-97 (1) --------------------------------------------------

P. redivivus NLP-97 (1) --------------------------------------------------

S. carpocapsae NLP-97 (1) --------------------------------------------------

S. feltiae NLP-97 (1) ----------------------MSAEHRANPPRLESRTDPRDSAAHSISL

S. monticolum NLP-97 (1) --------------------------------------------------

S. muris NLP-97 (1) --------------------------------------------------

S. scapterisci NLP-97 (32) IFVSSTFGHKLHLMTNFLLFARSKLDIRDEEGPKEPEEATTFHTRFPRHS

S. vulgaris NLP-97 (1) --------------------------------------------------

T. canis NLP-97 (1) --------------------------------------------------

T. circumcincta NLP-97 (1) --------------------------------------------------

101 150

A. caninum NLP-97 (1) ---------------------MSPLSILCVLLLVYTISAEVVSEIRGTNE

A. ceylanicum NLP-97 (1) --------------------------------------------------

A. costaricensis NLP-97 (1) -------------------------------MFHISYFYVLLLVISAAAE

A. duodenale NLP-97 (1) -----------------------------------------------MEW

A. lumbricoides NLP-97 (1) ------MLAKFIVVTVALLEFGYSGGNADLSADTVDVAKRSRNPYSWMNE

A. simplex NLP-97 (1) ------MIQKLVVLFFTMIIVDFTQCSVDESVDESDVSKRSRNPYSWMNN

A. suum NLP-97 (1) --------MCESGCSGKHHVKAYLARATPLVMFGVYVSKRSRNPYSWMNE

B. malayi NLP-97 (1) -------------------------------MCHYHIISYHFIIISIIII

B. xylophilus NLP-97 (4) VNHPRVERRGEARGWATPFGGNGGLFARSLIVNCYLDKGRQVGNYSTRDS

D. destructor NLP-97 (2) GYSQSSIFRFSTVFVEMFFCIVSIGLLACGGINAAPGSGVREVDKTEVDT

D. immitis NLP-97 (1) ----------------MLYLMNPNIGNVKMDRYRYSIPFYSIIIISTIII

D. medinensis NLP-97 (1) --------MMGNILLLYFLLVTRMFYANAKSDVIGDDDKRSRNPYSWMNY

D. viviparus NLP-97 (1) ------------------------------------MLLLVCIVSVTVNS

E. vermicularis NLP-97 (1) -----------------------------------------------MEE

G. pallida NLP-97 (1) ----MSSFTFTSSTTGFIFSALFVSASFAFPPFVGQKAEQDKRGFPVRNP

H. bacteriophora NLP-97 (101) VWTSVGKVTQRYLSTQMINSITISSETHFRNGEYTEGLQYMVDSYTSILN

H. contortus NLP-97 (1) -----------------------------MSPLSLLCALLVVYTALADVN

H. placei NLP-97 (1) -----------MLGRDCGCPNRVTAADNGIFDKDLLLTEKQANFLLNELG

H. polygyrus NLP-97 (1) --------------------------------------------------

N. americanus NLP-97 (1) ---------------------MSSLSFLFVLLLVYTISADAVNEIQGTNE

O. dentatum NLP-97 (1) --------------------------------------------------

P. pacificus NLP-97 (1) ---------------------------------------MARSLFSSLLL

P. redivivus NLP-97 (1) -------------------MLSQPWILFIFASAIGGAAVIADVPVDETDK

S. carpocapsae NLP-97 (1) -------MSRPLICCLLALGVAHVLAFEDSPP--SEMIKRPMNPYSWMNK

S. feltiae NLP-97 (29) ARSIVAQMTRPLICCLLALGVA--LAYDDASSSVSEMAKRPQNPYSWMNK

S. monticolum NLP-97 (1) -------MTRHLICCLLALSVAQVLSFDDSRSAGADLVKRPQNPYSWMNK

S. muris NLP-97 (1) -----MVVTSAVLQTGVVIVNLFLIISALEVSGPKTDSKRARNPYSWLEE

S. scapterisci NLP-97 (82) TTYHLAEMTRPLICCLLALGVANVLAFEDSPPSASEMVKRPLNPYSWMNK

S. vulgaris NLP-97 (1) --------------------------------------------------

T. canis NLP-97 (1) -----------------------------------------------MNE

T. circumcincta NLP-97 (1) -----------------------------MSPLSLLCALLVVYTVLADVN

151 200

A. caninum NLP-97 (30) DENSMDIT----------EEKRMHARNPYSWMK-VKRAR-NPYSWMLSEQ

A. ceylanicum NLP-97 (1) -------------------MVTYEDSKLTAPGLEISSFT-YRAVMNRSRK

A. costaricensis NLP-97 (20) SQLQHPN----------EHVMNEDVAKILPFWTDVKRAR-NPYSWMAVEQ

A. duodenale NLP-97 (4) KGVRDACQ----------ESVLVDESETSTKSLLLDAQR-ARQEGKKPIQ

A. lumbricoides NLP-97 (45) EKRIDN-------PYAWTNVNEN--MLGLDLDELEKRAR-NPYSWMNYKE

A. simplex NLP-97 (45) LKRTDA-------LYQWLPNSENSNFNEIEAETSDKRAIKNPYSWQNYKV

A. suum NLP-97 (43) EKRMDN-------PYAWTNVNEN--MLGLDLDELEKRAR-NPYSWMNYKE

B. malayi NLP-97 (20) PTIANPAIG---------NIYEKPS-KYFTYKNEPDNYGFMPEEYFNTAK

B. xylophilus NLP-97 (54) VNGFGDMALLRPSIVVALTLVVVYGLDSFETTKRRLGRPMNPYSWQNGGY

D. destructor NLP-97 (52) KKPESPDTQFLVQSGVEAAAFRKRAYELASMFDQYYGSLQNEQSPLTEEH

D. immitis NLP-97 (35) ATTASPTN----------DIYDKSSNHFALYKNEPNNDDSMLDEYFVADK

D. medinensis NLP-97 (43) ARVSRSP--AQWSEQADENSIFPWGREFMDAGRLEKKAR-NPYSWMNTMY

D. viviparus NLP-97 (15) QLQQSDDE----------QMNEERLAKASPIWMNAKRAR-NPYSWMAAEE

E. vermicularis NLP-97 (4) KRSGSR--VANKADMLAAPLPEEYENMFEESSEMQKRFR-NPYSWMNDLD

G. pallida NLP-97 (47) YSWMAQLQ--------KRSVPSGMDNTSPSDGELPNLLLEEPSKRFPSPA

H. bacteriophora NLP-97 (151) GEHLEVIS----------NAVSKRARNPYSWME-TKRAR-NPYSWMTEQG

H. contortus NLP-97 (22) DEAEQHDR----------DLLQLNDDGISS----VKRAR-NPYSWMVVDQ

H. placei NLP-97 (40) KAGEGADV----------PPPGNGAKKFKSMSFLHCNCH-PASVNIVLET

H. polygyrus NLP-97 (1) ------------------------------------MAR-KRGRHMPGQC

N. americanus NLP-97 (30) DENAIDIN----------EEKRVHSRNPYSWMSQEKRAR-NPYSWMVSE-

O. dentatum NLP-97 (1) -------------------------------MM-DKRAR-NPYSWMATEQ

P. pacificus NLP-97 (12) LAILAS---------FIISILALDDIEE---EKRGRNP----YSWQNVDK

P. redivivus NLP-97 (32) TEVSTPYQGPAWLKRIPDNLNAFAPDELPLYKRPVGHVSRNPYSWMATNT

S. carpocapsae NLP-97 (42) YRVKKS---------HPVDMVNEADEGIM-EDEMEKRAR-NPYAWTETIK

S. feltiae NLP-97 (77) YRVKKS---------HPDADDIILDGVGGDVEDVEKRAR-NPYAWTVTDE

S. monticolum NLP-97 (44) YRVKKS---------HPLALDEDVEVLG--EDDIEKRAR-NPYAWTVTDE

S. muris NLP-97 (46) KRGGFRSEVPNKFDLTAPALPEEYDTLLENTFEIPKRAR-NPYSWMNDLE

S. scapterisci NLP-97 (132) YRVKKS---------HPVDMANEADEGIM-EEEMEKRAR-NPYAWTETMK

S. vulgaris NLP-97 (1) -------------------------------MK-VKRTR-NPYSWMASEQ

T. canis NLP-97 (4) DKRSG--------LYEWLQGNDN--LNDFDIDEMPKRAR-NPYSWMNYME

T. circumcincta NLP-97 (22) DEVQQQDR----------ELLQLNDDGISSGSA-IKRAR-NPYSWMVTDQ

201 250

A. caninum NLP-97 (68) G----------------------KRARNPYSWMVME-------------K

A. ceylanicum NLP-97 (31) G----------------------DSSLASK---DEL-------------Q

A. costaricensis NLP-97 (59) G----------------------MGGSNPYSWMTQMSKTNFAQIFLYEFK

A. duodenale NLP-97 (43) L----------------------DGYGEGENCCLDW-------------K

A. lumbricoides NLP-97 (85) K----------------------RALRNPYSWMA----------------

A. simplex NLP-97 (88) VNF------------------GFLAPREPYWWMG----------------

A. suum NLP-97 (83) K----------------------RALRNPYSWMA----------------

B. malayi NLP-97 (60) R----------------------SRNSYSWMSNSAI-------------K

B. xylophilus NLP-97 (104) KIKRSLENELETETGALPEEKRAPMPRNPYSWLARS--------------

D. destructor NLP-97 (102) KRA------------------GSVPSRNPYSWMAVE-------------E

D. immitis NLP-97 (75) R----------------------FQN------DKGI-------------K

D. medinensis NLP-97 (90) G------------------------ERDPYGWMAFG-------------G

D. viviparus NLP-97 (54) V----------------------NSARNPYYWMGQT-------------K

E. vermicularis NLP-97 (51) K-----------------------RSRNPYSWMN----------------

G. pallida NLP-97 (89) ASFG------------------GRRPQNPYSWMSFG--------------

H. bacteriophora NLP-97 (189) -----------------------KRARNPYSWLAKE-------------K

H. contortus NLP-97 (57) S----------------------KRSRNPYSWMVHS-------------K

H. placei NLP-97 (79) S----------------------ENHGLVWAVSIET-------------Q

H. polygyrus NLP-97 (14) A----------------------VGPEGGHRSEDES-------------R

N. americanus NLP-97 (68) G----------------------KRSRNPYSWMAME-------------K

O. dentatum NLP-97 (18) D----------------------KRARNPYSWMASE-------------K

P. pacificus NLP-97 (46) R------------------------GRNPYSWMS-G-------------T

P. redivivus NLP-97 (82) P---------------------SKRSRNPYAWLALA-------------E

S. carpocapsae NLP-97 (81) ---------------------GKRGARNPYSWLN---------------V

S. feltiae NLP-97 (117) RFM------------------GKRGARNPYSWMNNN-------------A

S. monticolum NLP-97 (82) RFM------------------GKRGARNPYSWMN-D-------------A

S. muris NLP-97 (95) K-----------------------RSRNPYSWMN----------------

S. scapterisci NLP-97 (171) ---------------------GKRGARNPYSWLN---------------V

S. vulgaris NLP-97 (18) Y----------------------KRARNPYSWMVSE-------------K

T. canis NLP-97 (43) K----------------------RSPRNPYSWMA----------------

T. circumcincta NLP-97 (60) G----------------------KRSRNPYSWMAHP-------------K

251 300

A. caninum NLP-97 (83) -DKR---TLRFSDDVYRQPRNPYSWMYTMF--------------------

A. ceylanicum NLP-97 (43) -DKR---ALRFSDDVYRQPRNPYSWMYTMF--------------------

A. costaricensis NLP-97 (87) LGGSAEKTARNIEEVWGESNAGVSAVQTWFRKFQSG--------------

A. duodenale NLP-97 (58) -DKR---ALRFSDDVYRQPRNPYSWMYTMF--------------------

A. lumbricoides NLP-97 (97) ------------FGEKRAPLNPYSWMYTLQ--------------------

A. simplex NLP-97 (104) ------------YGEKRAPLNPYSWMYTLQ--------------------

A. suum NLP-97 (95) ------------FGEKRAPLNPYSWMYTLQ--------------------

B. malayi NLP-97 (75) SQPR---N-MLIVLEKPAPLNPYSWQYMMKK-------------------

B. xylophilus NLP-97 (140) --------QKEGPYQQKRTRDAYFWNNQDFLLDSLFRSETKKSP------

D. destructor NLP-97 (121) KRSRNPYSWLAKNDDEKRSRNPYSWLATNDGSIDRTKTWGDAPPPPKRLA

D. immitis NLP-97 (84) LPP------VLIAFEKPVPMNPYSWQYMVKK-------------------

D. medinensis NLP-97 (103) ---------------KRAPMNPYSWQYMLA--------------------

D. viviparus NLP-97 (69) -GKR---LIRYADQIYSHPRNPYSWMYTMF--------------------

E. vermicularis NLP-97 (62) ------------FGFKRAPMNPYSWQNA----------------------

G. pallida NLP-97 (107) ------DADQSNMDWLFTRLRPFSELVSKRLNALG---------------

H. bacteriophora NLP-97 (203) GTKR---SPRFAEDVLRQPRNPYSWMYSIF--------------------

H. contortus NLP-97 (72) -GKR---SLRYFDDAYRQPRNPYSWLYTMF--------------------

H. placei NLP-97 (94) -GKR---SLRYFDDAYRQPRNPYSWLYTMF--------------------

H. polygyrus NLP-97 (29) -EKR---AVRFADDIYRQPRNPYSWMYTMF--------------------

N. americanus NLP-97 (83) -EKY---YLNYRLLSGQTCASFFR--------------------------

O. dentatum NLP-97 (33) -TKR---SVRFSEEFR-QPRNPYSWMYTMF--------------------

P. pacificus NLP-97 (58) KRAR---NPYSWMEFQKRARNPYSWME-----------------------

P. redivivus NLP-97 (98) DSAS---KRAYYPTFDKKSRNPYSWMSDVK--------------------

S. carpocapsae NLP-97 (95) KPKR---SPYAYFSEQRAPLNPYSWMSSMV--------------------

S. feltiae NLP-97 (136) KPKR---G-YAFFAEQRMPLNPYSWMSSMV--------------------

S. monticolum NLP-97 (100) KPKR---S-YAFFAEQRMPLNPYSWMSSMV--------------------

S. muris NLP-97 (106) ------------FGSKRAPMNPYSWQYA----------------------

S. scapterisci NLP-97 (185) KPKR---S-YSYFSEQRAPLNPYSWMSSMV--------------------

S. vulgaris NLP-97 (33) -NKR---SLRFADDGFRQPRNPYSWMYTML--------------------

T. canis NLP-97 (55) ------------YGEKRAPLNPYSWMYTLQ--------------------

T. circumcincta NLP-97 (75) -GKR---SLRFNDDGFRQPRNPYSWLYTMF--------------------

301 340

A. caninum NLP-97 (109) -----------DKRARNPYSWMNE----------------

A. ceylanicum NLP-97 (69) -----------DKRARNPYSWMNE----------------

A. costaricensis NLP-97 (123) ------DFDLEDEEGRGCPNELNDDEFDCLQHVMFSKMPS

A. duodenale NLP-97 (84) -----------DKRARNPYSWMNE----------------

A. lumbricoides NLP-97 (115) -----------KRAPFGYFRNPYSWLNSPASW--------

A. simplex NLP-97 (122) -----------KRSAFGRARNPYSWLNTSR----------

A. suum NLP-97 (113) -----------KRAPFGYFRNPYSWLNSPASW--------

B. malayi NLP-97 (102) ---------KNQIILRNPYSWMNNHNN-------------

B. xylophilus NLP-97 (176) ------YVGFNGRRARNPYSWMNFV---------------

D. destructor NLP-97 (171) RVGPMSWAEGLKRGAKNPYSWMNYA---------------

D. immitis NLP-97 (109) ---------KSGKIARNPYSWMNNIY--------------

D. medinensis NLP-97 (118) -----------KRALRNPYSWQMAD---------------

D. viviparus NLP-97 (95) -----------DKRTNTPYSWMNE----------------

E. vermicularis NLP-97 (78) ----------------------------------------

G. pallida NLP-97 (136) -------MGMGFRRPKNPYLWAMNRQLLAE----------

H. bacteriophora NLP-97 (230) -----------EKRTRNPYSWMNE----------------

H. contortus NLP-97 (98) -----------DKRARNPYSWMNE----------------

H. placei NLP-97 (120) -----------DKRARNPYSWMNE----------------

H. polygyrus NLP-97 (55) -----------EKRARNPYSWMNTDA--------------

N. americanus NLP-97 (103) ----------------------------------------

O. dentatum NLP-97 (58) -----------DKRARNPYSWMNYE---------------

P. pacificus NLP-97 (82) ----------------------------------------

P. redivivus NLP-97 (125) -------------RARNPYAWVNYV---------------

S. carpocapsae NLP-97 (122) -----------KKSRNPS-GYFYVDERSPVRNPYSWLN--

S. feltiae NLP-97 (162) -----------KKSRSPSPQYFFVDERSPIRNPYSWQN--

S. monticolum NLP-97 (126) -----------KKSRTPS-QYFFVDERSPIRNPYSWQN--

S. muris NLP-97 (122) ----------------------------------------

S. scapterisci NLP-97 (211) -----------KKSRNPS-GYFYVDERSPVRNPYSWLN--

S. vulgaris NLP-97 (59) -----------EKRTRNPYSWMNYE---------------

T. canis NLP-97 (73) -----------KRVPVRYFRNPYSWQSSSASW--------

T. circumcincta NLP-97 (101) -----------DKRARNPYSWMNE----------------

NLP-98 Pan-phylum

1 50

G. pallida NLP-98.1 (1) --------------------------------------------------

G. rostochiensis NLP-98.1 (1) ----------------------------------------MIDAASIPLQ

G. pallida NLP-98.2 (1) ----------------------------------------MIVVALIPLQ

G. rostochiensis NLP-98.2 (1) MIVIALIPLQLMAIAVASALKPFFTLTTLCRLQLCKPSKAMIVIALIPLQ

51 100

G. pallida NLP-98.1 (1) --------------------------------------------------

G. rostochiensis NLP-98.1 (11) LMAIVGAAVLVIIAQCCGCGKK--NDKAEVPPTPAPAEQPDAASKKSQAP

G. pallida NLP-98.2 (11) LMAIVVAAVLVIITQCCGGGKKNTEEPPQPPQASAVAEQPTTTAAGQSQN

G. rostochiensis NLP-98.2 (51) LMAIAVASALVIITQCCGGGKKNTEEPPPPPQMSAVAEQPTTTAAGQSQN

101 150

G. pallida NLP-98.1 (1) --------------------------------------------------

G. rostochiensis NLP-98.1 (59) TPTAVDQLG---TASKNGPPIATTTGYSTGG-------------------

G. pallida NLP-98.2 (61) APTSVAEPQ-GATVSKNAPALGATTNYAAPGSTENIFSQSPSTSAAPGAA

G. rostochiensis NLP-98.2 (101) PPTSAVAEQPSPTASKNAPALGATTNYAAPGSTENIFSKMS---------

151 200

G. pallida NLP-98.1 (1) -----------DPGGVSKKSLAP---STSAADPGGVSKKSLAPSTSAADP

G. rostochiensis NLP-98.1 (87) G--------GADMNIFSKMSTAGEPKPPAADLGAVSKKSAIGPTAAAADL

G. pallida NLP-98.2 (110) SKKSVAPTTSASPGAVSKKSVAP---TTSASPGAVSQKS--VAPTTSASP

G. rostochiensis NLP-98.2 (142) ---AQSPTTSAAPGAVSKKSVAP---TTSAAPGAVSKKS--VAPTTSAAP

201 248

G. pallida NLP-98.1 (37) GGVSKKSLAPSTSAGVTKAQNNQSIALGAPGARSTRI-----------

G. rostochiensis NLP-98.1 (129) GAVSKKSLAPTTSAADLGATVCNSIYIKSSGDESVWMKNLFV------

G. pallida NLP-98.2 (155) GAVSKKSVAPTTSAS-PAAVSQKSAAPTTSAADPGGVSKK---SSAAP

G. rostochiensis NLP-98.2 (184) DAVSKKSVAPTTSAADPAGVSKKSAAPTTSAADPGGVSKKSAAGSAAP
