# Supplementary figures and images for: In silico analyses of neuropeptide-like protein (NLP) profiles in parasitic nematodes
Source: Int J Parasitol. 2022 Jan;52(1):77–85. doi: 10.1016/j.ijpara.2021.07.002 (PMC8764417; doi:10.1016/j.ijpara.2021.07.002)

**A**


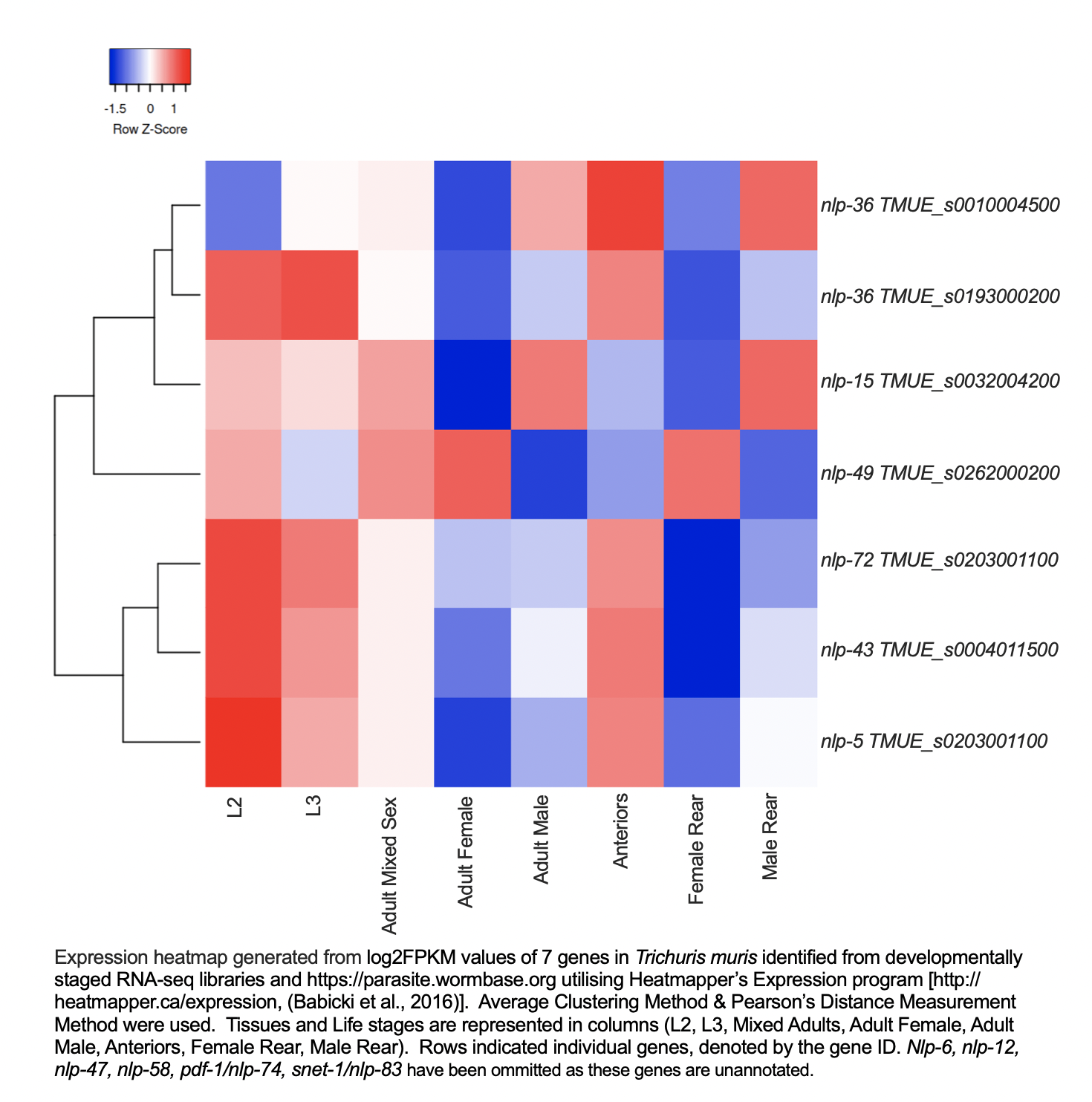


**B**


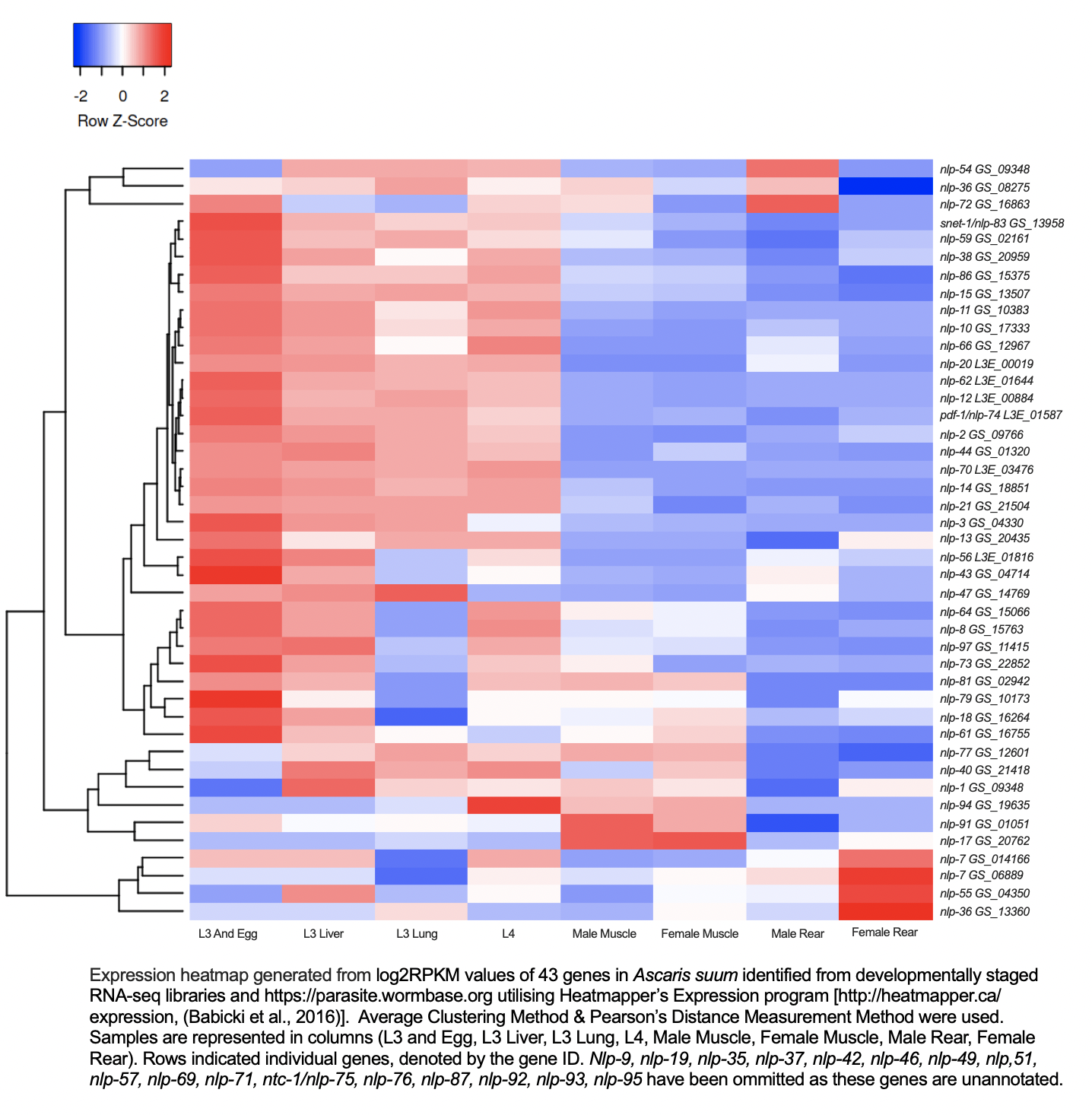


**C**


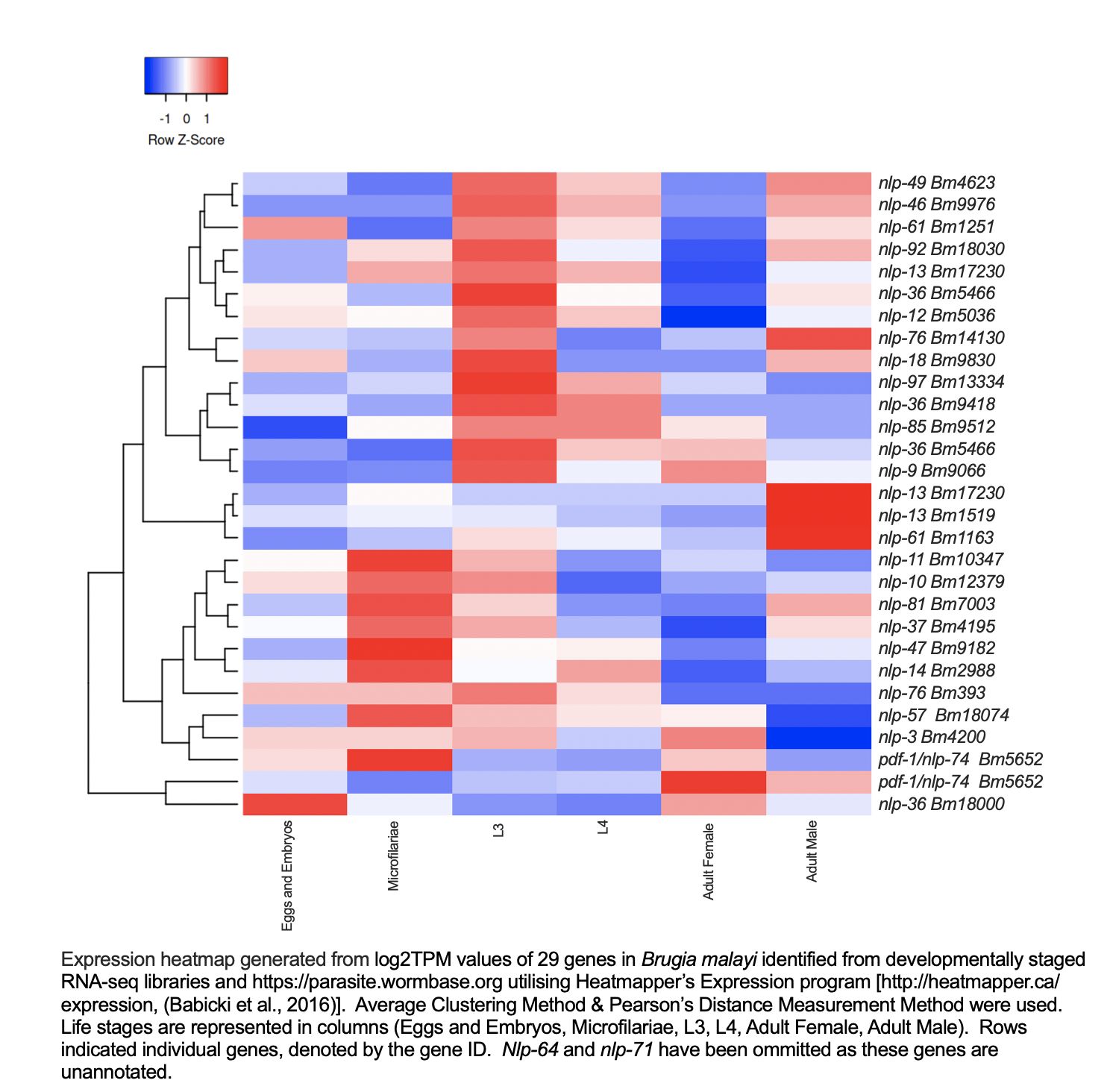


**D**

*
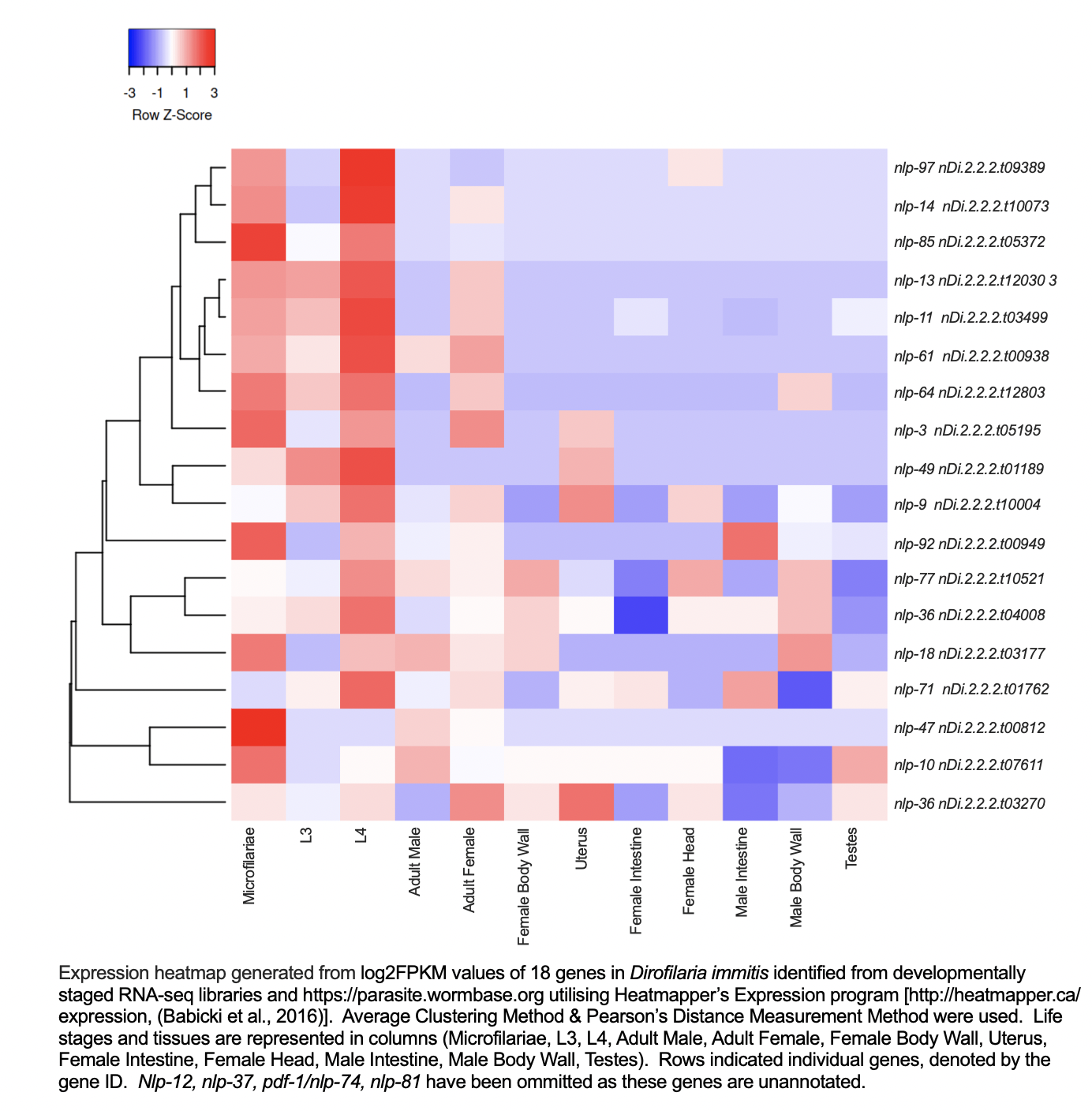
*

**E**


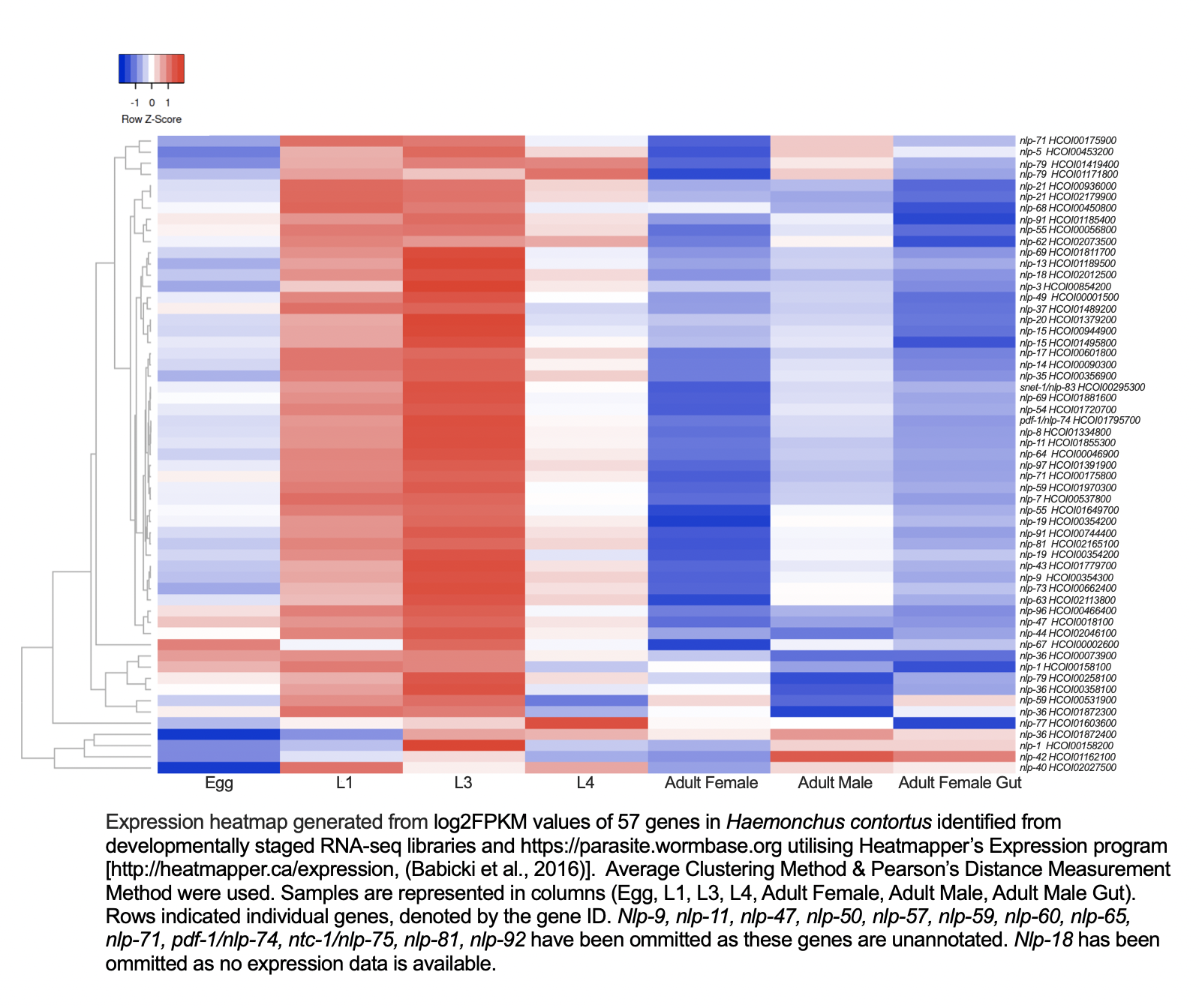


**F**


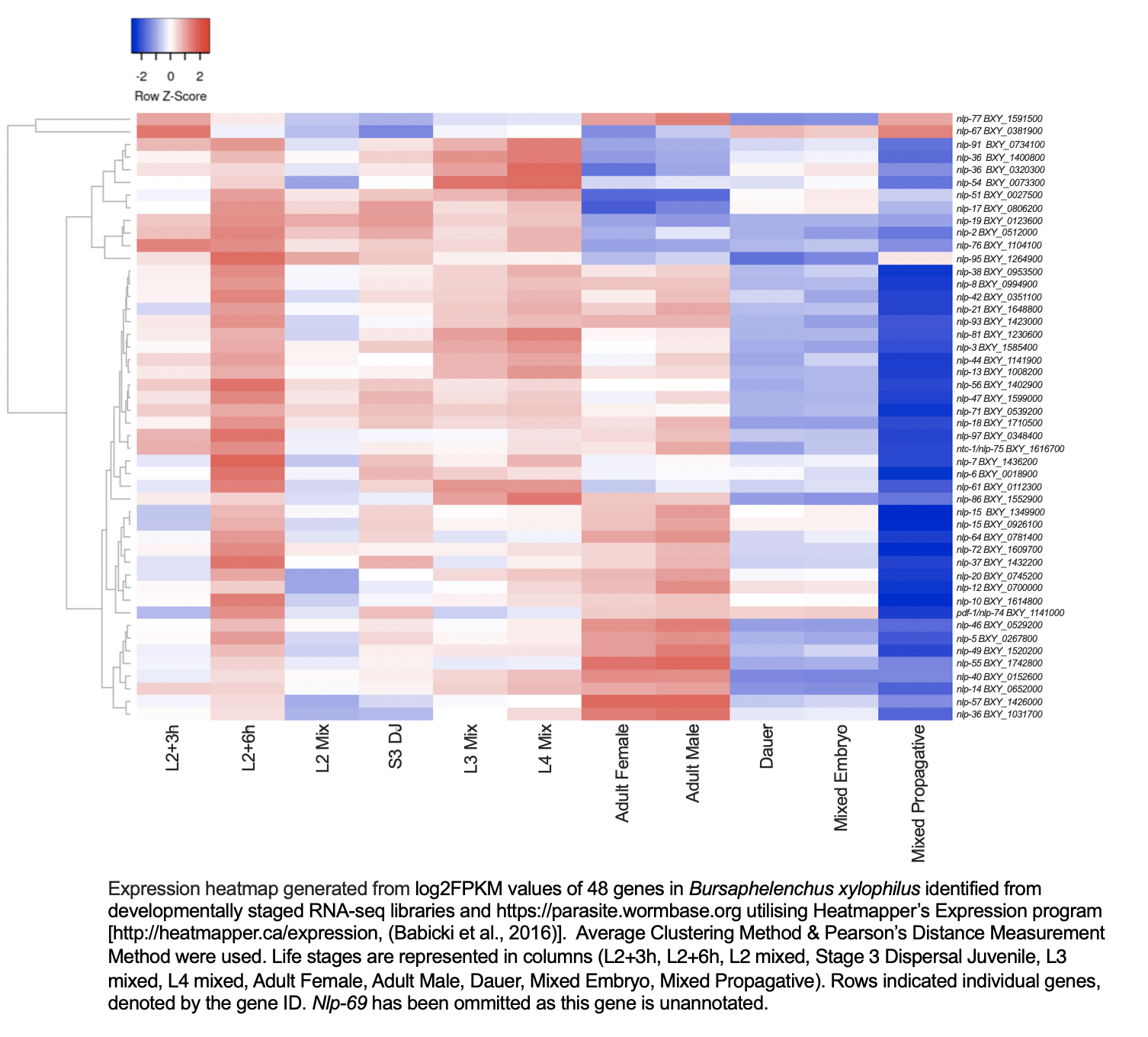


**G**


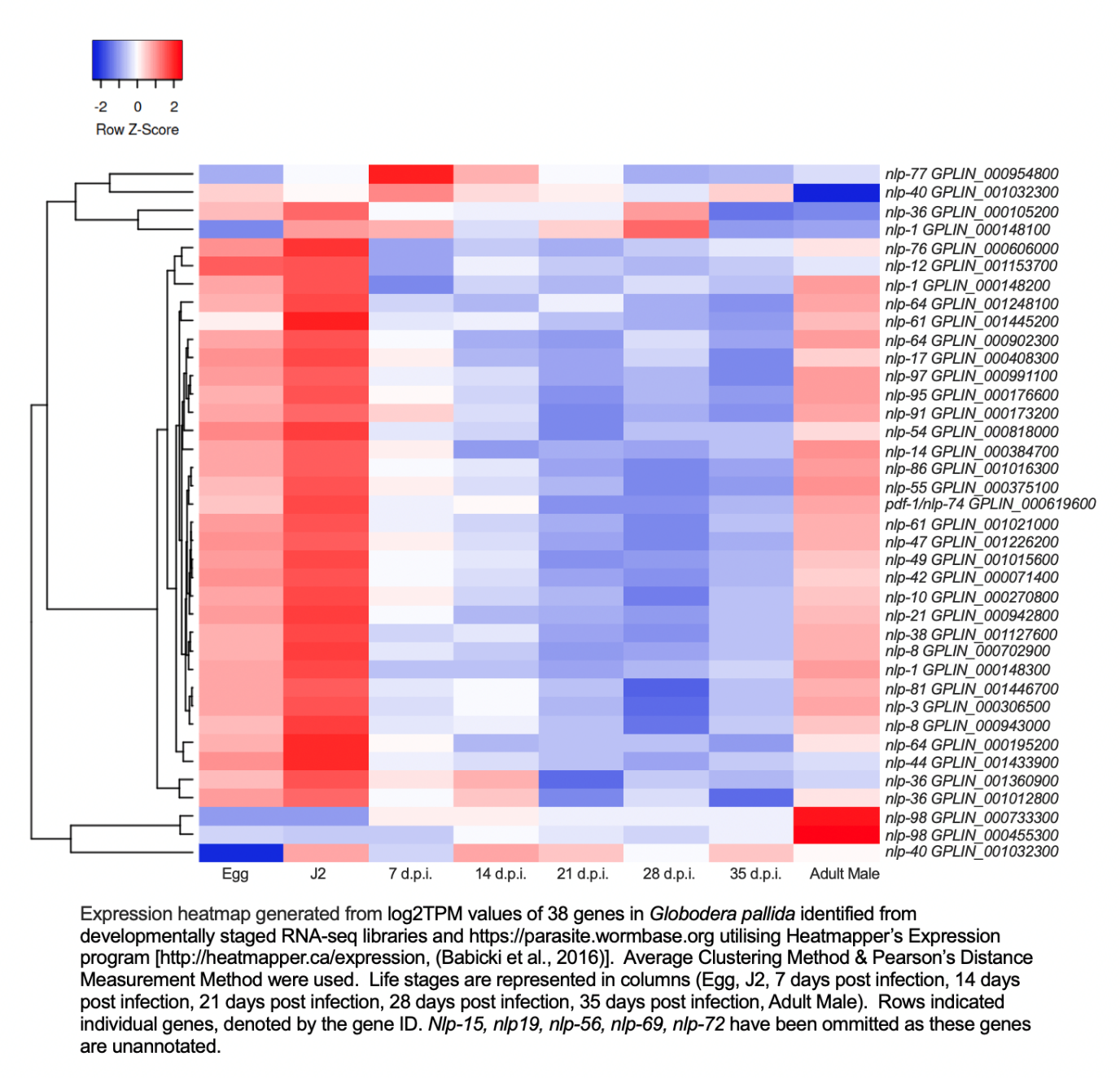

Supplement: Supplementary Fig. S2 — Expression Heatmaps for nematode neuropeptide-like protein (NLP) genes expressed in lifecycle stages of (A) Trichuris muris, (B) Ascaris suum, (C) Brugia malayi, (D) Dirofilaria immitis, (E) Haemonchus contortus, (F) Bursaphelenchus xylophilus, (G) Globodera pallida. (A) Expression heatmap generated from log2FPKM values of seven genes in T. muris identified from developmentally staged RNA-seq libraries and https://parasite.wormbase.org utilising the Expression program in Heatmapper (http://heatmapper.ca/expression; Babicki et al. (2016)). Average Clustering Method and Pearson’s Distance Measurement Method were used. Tissues and Life stages are represented in columns (L2, L3, Mixed Adults, Adult Female, Adult Male, Anterior, Female Rear, Male Rear). Rows indicate individual genes, denoted by the gene ID. nlp-6, nlp-12, nlp-47, nlp-58, pdf-1/nlp-74, snet-1/nlp-83 have been omitted as these genes are unannotated. (B) Expression heatmap generated from log2RPKM values of 43 genes in A. suum identified from developmentally staged RNA-seq libraries and https://parasite.wormbase.org utilising the Expression program in Heatmapper (http://heatmapper.ca/expression; Babicki et al. (2016)). Average Clustering Method and Pearson’s Distance Measurement Method were used. Samples are represented in columns (L3 and Egg, L3 Liver, L3 Lung, L4, Male Muscle, Female Muscle, Male Rear, Female Rear). Rows indicate individual genes, denoted by the gene ID. nlp-9, nlp-19, nlp-35, nlp-37, nlp-42, nlp-46, nlp-49, nlp-51, nlp-57, nlp-69, nlp-71, ntc-1/nlp-75, nlp-76, nlp-87, nlp-92, nlp-93, nlp-95 have been omitted as these genes are unannotated. (C) Expression heatmap generated from log2TPM values of 29 genes in B. malayi identified from developmentally staged RNA-seq libraries and https://parasite.wormbase.org utilising the Expression program in Heatmapper (http://heatmapper.ca/expression; Babicki et al. (2016)). Average Clustering Method and Pearson’s Distance Measurement Method were used. L [file mmc2.docx]
