# Supplementary material for: In silico analyses of neuropeptide-like protein (NLP) profiles in parasitic nematodes
Source: Int J Parasitol. 2022 Jan;52(1):77–85. doi: 10.1016/j.ijpara.2021.07.002 (PMC8764417; doi:10.1016/j.ijpara.2021.07.002)
Supplement: Supplementary Table S6 [file mmc8.docx]

**Supplementary Table S6.** The effects of *Ascaris suum* neuropeptide-like protein (*As*-NLP) peptides on the ovijector of *A. suum.* Contraction frequency and amplitude recorded 2 min prior to each timepoint, presented as a percentage of time 0. Change in tension is presented relative to time 0, where muscle relaxation caused an increase in circular muscle tension (+mg/mm; shortening of the tissue) and circular muscle contraction caused a decrease in tension (-mg/mm; lengthening of tissue).

| **Peptide tested**  [10µM] | **Time**  (min p.a.) | **Contraction frequency**  (% 0 min, 2 min  prior to min p.a.) | **Contraction amplitude**  (% 0 min, 2 min  prior to min p.a.) | **Change in tension**  (mg/mm) |
| --- | --- | --- | --- | --- |
| **NLP-12A1** (DLTPTRFDRQDRDYRPLQF-NH_2_)  *n*≥3 | N/A | No effect | No effect | No effect |
| **NLP-12B** (DGYRPLQF-NH_2_)  *n*≥3 | N/A | No effect | No effect | No effect |
| **NLP-12C** (SYRPLQF-NH_2_)  *n*≥3 | N/A | No effect | No effect | No effect |
| **NLP-12D** (AYDFSEYTPY)  *n*≥3 | N/A | No effect | No effect | No effect |
| **NLP-36A1** (DDRTVFEKTE)  *n*≥3 | N/A | No effect | No effect | No effect |
| **NLP-36A2** (DDRTVFE)  *n*≥3 | N/A | No effect | No effect | No effect |
| **NLP-36B1** (TERTHTEAAWGL)  *n*=6 | 50secs  2  5  10  **overall** | N/A  ↓ 89.34±13.27 🗴  ↑ 119.40±7.37 🗴  ↑ 108.20±8.12 🗴  ***P*=0.0268, F=4.065** | N/A  ↓ 82.21±8.05 🗸  ↓ 72.95±5.94 🗸  ↓ 77.28±6.97 🗸  ***P*=0.0049, F=6.514** | Lengthening -0.046±0.026 🗴  Shortening +0.002±0.024 🗴  Shortening +0.018±0.029 🗴  Shortening +0.029±0.024 🗴  **NS** |
| **NLP-36B2** (THTEAAWGL)  *n*=6 | 50 secs  2  5  10  **overall** | N/A  ↑ 147.50±45.15 🗴  ↑ 105.20±19.85 🗴  ↑ 137.90±24.66 🗴  **NS** | N/A  ↓ 77.31±5.14 🗴  ↓ 83.59±12.10 🗴  ↓ 71.44±5.47 🗸  ***P*=0.0379, F=3.623** | Shortening +0.011±0.008 🗴  Shortening +0.017±0.015 🗴  Shortening +0.031±0.008 🗸  Shortening +0.019±0.012 🗴  **NS** |
| **NLP-36C1** (HPDAGFLLDSSENF)  *n*≥3 | N/A | No effect | No effect | No effect |
| **NLP-36C2** (HPDAGFLLDSSENFRVIGFI)  *n*≥3 | N/A | No effect | No effect | No effect |
| **NLP-47A** (pQITFTDQWT)  *n*=6 | 50 secs  2  2.5  5  10  **overall** | N/A  ↑ 114.70±6.73 🗴  N/A  ↑ 131.40±11.58 🗸  ↑ 132.10±10.78 🗸  **P=0.0219, F=4.328** | N/A  ↑ 102.20±7.71 🗴  N/A  ↓ 94.73±8.59 🗴  ↓ 86.03±11.12 🗴  **NS** | Lengthening -0.043±0.034 🗴  Lengthening -0.032±0.023 🗴  Lengthening -0.065±0.051 🗴  Lengthening -0.010±0.019 🗴  Shortening +0.006±0.020 🗴  **NS** |
| **NLP-49** (ASCYSVSTFWLSHIFIAASMGLSLAEYMASPQGQDNFHFIPS-NH_2_)  *n*=6 | 2  5  10  **overall** | ↑ 122.60±29.90 🗴  ↑ 105.30±49.97 🗴  ↓ 93.02±36.82 🗴  **NS** | ↓ 79.30±7.09 🗴  ↓ 71.78±13.33 🗸  ↓ 72.81±14.98 🗸  **NS** | Shortening +0.067±0.015 🗸  Shortening +0.068±0.016 🗸  Shortening +0.068±0.013 🗸  **P=0.0008, F=7.308** |
| **PDF-1A** (SNAELINGLIGMDLNKLSAI-NH_2_)  *n*=6 | 2  5  10  **overall** | ↑ 110.50±6.74 🗴  ↑ 123.70±15.62 🗴  ↑ 136.80±16.30 🗸  **NS** | ↓ 88.52±15.94 🗴  ↓ 93.64±21.53 🗴  ↓ 85.72±21.16 🗴  **NS** | Shortening +0.025±0.019 🗴  Shortening +0.027±0.014 🗴  Shortening +0.031±0.012 🗴  **NS** |
| **PDF-1B** (SNAELINGLLGMNLNRLSSA-NH_2_)  *n*=6 | 2  5  10  **overall** | ↑ 158.50±36.26 🗴  ↑ 148.20±42.84 🗴  ↑ 149.00±48.25 🗴  **NS** | ↓ 60.61±7.33 🗸  ↓ 75.48±12.82 🗴  ↓ 80.97±18.36 🗴  ***P*=0.0320, F=3.836** | Shortening +0.005±0.005 🗴  Lengthening -0.019±0.025 🗴  Lengthening -0.018±0.026 🗴  **NS** |
| **PDF-2C** (NNAEVVNHILKNFGALDRLGDV-NH_2_)  *n*≥3 | N/A | No effect | No effect | No effect |

p.a., post addition; overall, indicates the significance of the Repeated Measures ANOVA test comparing pre- and post-addition; NS, results not statistically significant; *P*, *P* value (significant when *P*< 0.05); F, F ratio. ↑, increase in contraction frequency/amplitude; ↓, decrease in contraction frequency/amplitude. 🗸, significant Dunnet’s post test; 🗴, non-significant Dunnet’s post test.
